# Solvent-controlled Rh-catalysed hydrodehalogenation and construction of phenanthridinone skeleton from a common precursor in one-step sequence and the antitumor activity of its derivatives

**DOI:** 10.1080/14756366.2026.2704432

**Published:** 2026-07-28

**Authors:** De-Xuan Hu, Chao Qin, Xiang Gao, Ling Tang, Yaxin Zheng, Rui Yin

**Affiliations:** ^a^ Key Laboratory of Structure-Specific Small Molecule Drugs at Chengdu Medical College of Sichuan Province, School of Pharmacy, Chengdu Medical College, Chengdu, China; ^b^ Sichuan Clinical Research Center for Radiation and Therapy, The Second Affiliated Hospital of Chengdu Medical College, Nuclear Industry 416 Hospital, Chengdu, China; ^c^ Guangdong Institute for Drug Control, Guangzhou, China; ^d^ School of Pharmaceutical Sciences, Sun Yat-sen University, Guangzhou, China

**Keywords:** Rhodium-catalysed, solvent-controlled, phenanthridinone skeleton, hydrodehalogenation, anticancer

## Abstract

Herein, we report a solvent-controlled, operationally convenient and highly efficient rhodium(II)-catalysed protocol enabling hydrodehalogenation and phenanthridinone skeleton construction from 2-halobenzamide. This methodology facilitates the hydrodehalogenation of diverse 2-halobenzamides using isopropanol, providing quantitative yields without further purification. Furthermore, this strategy allows the direct and efficient conversion of 2‑halobenzamides into phenanthridinones by aprotic solvent. Additionally, a series of phenanthridinone derivatives were synthesised and evaluated for their inhibitory activities against tyrosyl-DNA phosphodiesterase 1 (TDP1) and topoisomerase IB (TOP1), as well as their cytotoxicity. Compound **3a** showed potent TDP1 inhibitory activity (IC_50_ = 4.5 ± 0.4 μM) and synergistic effect with topotecan and radiosensitising effect in HCT116 cells by stabilising cellular TDP1 cleavage complexes (TDP1cc). Compound **3c** exhibited strong TOP1 inhibition (+++) and induced the formation of cellular TOP1 cleavage complexes (TOP1cc) and DNA damage, and consequently triggered apoptosis. *In vivo* studies indicated that **3c** exhibits antitumor efficacy in HCT116 xenograft model.

## GRAPHICAL ABSTRACT

**Figure:**
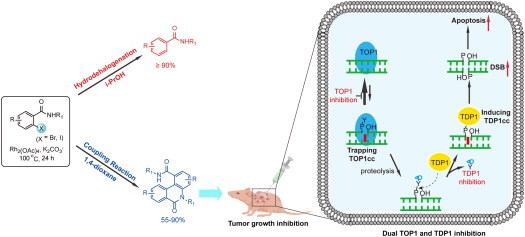

## Introduction

**Scheme 1.**
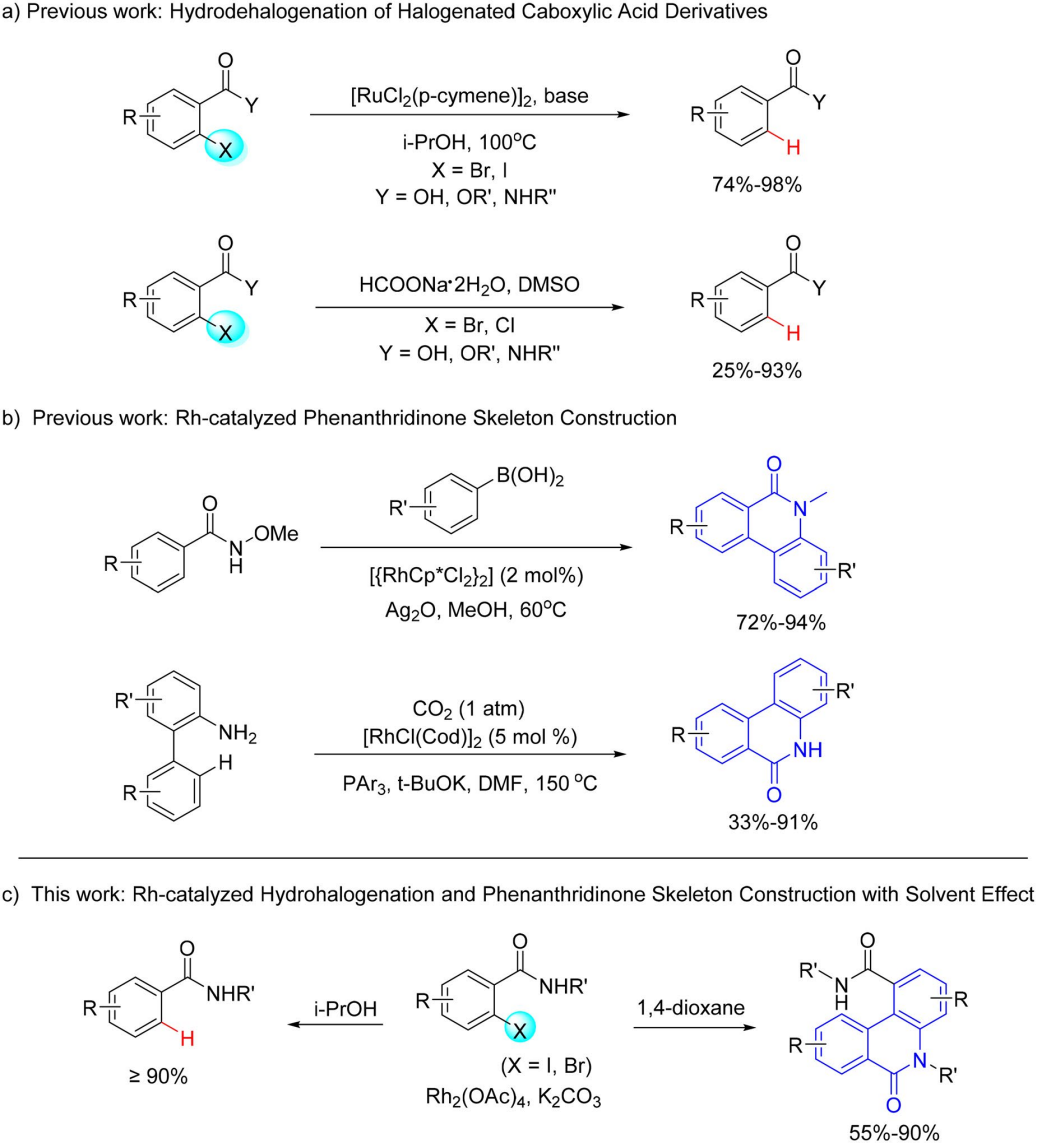
Approaches of hydrodehalogenation and phenanthridinone skeleton construction.

Reductive hydrodehalogenation has been recognised as a fundamental chemical transformation in medicinal chemistry, organic synthesis, and environmental science^1–4^. It is valuable for the late-stage modification of pharmaceuticals and fine chemical intermediates. Over the past few decades, substantial advances have been achieved by homogeneous catalytic systems of palladium^5^^,^^6^, copper^7^, ruthenium^8^^,^^9^, and other transition metals^10^^,^^11^. For instance, Xia and co-workers reported a facile and efficient Ru(II)-catalysed transfer hydrodehalogenation of organic halides in high yields^9^. Nevertheless, a major limitation of the existing methods is the common requirement for phosphine or other auxiliary ligands, which are typically toxic, air-sensitive, and synthetically challenging. Very few hydrodehalogenation methods proceeding under photo-, electrochemical, or metal-free conditions have been reported^3^^,^^12–15^. Wu and co-workers developed a practical hydrodehalogenation of halogenated carboxylic acid derivatives using a DMSO/HCOONa·2H_2_O system, albeit with moderate conversion efficiency (Scheme 1(a))^16^. Accordingly, the development of a novel, highly efficient hydrodehalogenation catalytic system without external ligands remains highly desirable.

The phenanthridinone skeleton represents a privileged structural motif present in numerous biologically active alkaloids, including phenaglydon^17^, HCK20^18^, oxynitidine^19^, ARC-111^20^, and PJ38^21^, which serve as promising candidates for anticancer, antibacterial, and antiviral applications (Figure S1). Although many classic synthetic strategies have been established for constructing the phenanthridinone skeleton, the exploration of more efficient and novel synthetic methods continues to attract considerable attention in synthetic chemistry^22–25^. Nevertheless, examples of Rh-catalysed coupling reactions for phenanthridinone skeleton construction remain rare. In 2012, Cheng and co-workers first reported a Rh(III)-catalysed oxidative C–H coupling of *N*-methoxybenzamides with aryl boronic acids to give phenanthridinones^26^. Another protocol catalysed by Rh(I) with CO_2_ had been reported subsequently (Scheme 1(b))^27^. Unfortunately, these two methods suffer from notable drawbacks, including pre-synthesised reagents, essential ligands, and the requirement for a controlled external atmosphere.

Herein, we report a solvent-controlled, ligand-free, and highly efficient method for Rh-catalysed hydrodehalogenation and phenanthridinone skeleton construction starting from 2-halobenzamides (Scheme 1(c)). In addition, we synthesised several phenanthridinone derivatives and evaluated their anticancer activities *in vitro* and *in vivo*.

## Results and discussion

### Chemistry

2-Bromobenzamide is a readily available aryl bromide derivative and important synthetic building block. Benzamide is a core parent structure of numerous bioactive molecules and organic functional materials. It is useful for the transformation of 2-bromobenzamide into benzamide for environmental science and the late-stage modification of pharmaceuticals^4^^,^^6^. Our investigation commenced with the conditions optimisation for transformation of 2-bromobenzamide (**1a**) into benzamide (**2a**) (Table 1). The reaction of **1a** was initially conducted using 5 mol% of Ni(dppe)_2_ as the catalyst in the presence of 1 mL isopropanol as both the hydrogen donor and solvent, and K_2_CO_3_ as the base at 100 °C for 24 h. Disappointingly, the yield of the desired product **2a** was extremely low (Table 1, entry 1). Replacement of the catalyst with palladium-based complexes, including Pd(OAc)_2_, Pd(pph_3_)_4_, and Pd(pph_3_)_2_Cl_2_, promoted the formation of **2a** (Table 1, entries 2–4), with Pd(pph_3_)_2_Cl_2_ showing the highest reactivity in 89% yield (Table 1, entry 4). To our surprise, an excellent yield of 99% was obtained using Rh_2_(OAc)_4_ as the catalyst (Table 1, entry 5). Apart from K_2_CO_3_, other common bases, such as Na_2_CO_3_, Cs_2_CO_3_, Et_3_N, and t-BuOK, were evaluated and found to be less effective for the desired transformation (Table 1, entries 6–9). The use of aprotic solvents including MeCN, THF, and 1,4-dioxane proved to be highly detrimental (Table 1, entries 10–12). The aprotic solvents had no additional hydrogen source which just played a role of solvent only. The trace water existing in commercial solvents might act as the main hydrogen sources resulting in low conversion. Results from entries 5 and 13 showed that the yield decreased dramatically when the reaction was conducted at a lower temperature. Finally, the effect of the reaction time was examined with 24 h being more suitable for the developed protocol (Table 1, entry 14). Accordingly, 5 mol% of Rh_2_(OAc)_4_ and 1.5 equiv. of K_2_CO_3_ in isopropanol solvent at 100 °C were established as the optimal conditions for the reduction of 2-bromobenzamide derivatives.

**Table 1.** Screening of the reaction conditions^a^.

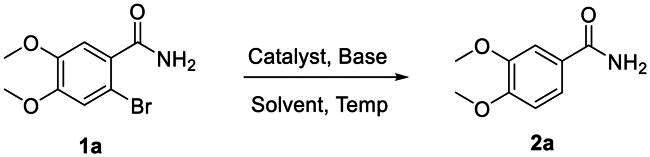						
Entry	Catalyst (5 mol%)	Base	Solvent	Temp. (°C)	Time (h)	Yield^b^ (%)
1	Ni(dppe)_2_	K_2_CO_3_	i-PrOH	100	24	5
2	Pd(OAc)_2_	K_2_CO_3_	i-PrOH	100	24	65
3	Pd(pph_3_)_4_	K_2_CO_3_	i-PrOH	100	24	67
4	Pd(pph_3_)_2_Cl_2_	K_2_CO_3_	i-PrOH	100	24	89
5	Rh_2_(OAc)_4_	K_2_CO_3_	i-PrOH	100	24	99
6	Rh_2_(OAc)_4_	Na_2_CO_3_	i-PrOH	100	24	97
7	Rh_2_(OAc)_4_	Cs_2_CO_3_	i-PrOH	100	24	92
8	Rh_2_(OAc)_4_	Et_3_N	i-PrOH	100	24	90
9	Rh_2_(OAc)_4_	t-BuOK	i-PrOH	100	24	85
10	Rh_2_(OAc)_4_	K_2_CO_3_	MeCN	100	24	5
11	Rh_2_(OAc)_4_	K_2_CO_3_	THF	100	24	10
12	Rh_2_(OAc)_4_	K_2_CO_3_	1,4-Dioxane	100	24	<5
13	Rh_2_(OAc)_4_	K_2_CO_3_	i-PrOH	rt	24	20
14	Rh_2_(OAc)_4_	K_2_CO_3_	i-PrOH	100	12	90

^a^ Reaction conditions: **1a** (0.2 mmol), catalyst (5 mol%), and base (1.5 equiv.) in solvent (1 mL).

^b^ Isolated yields.

**Figure 1.**
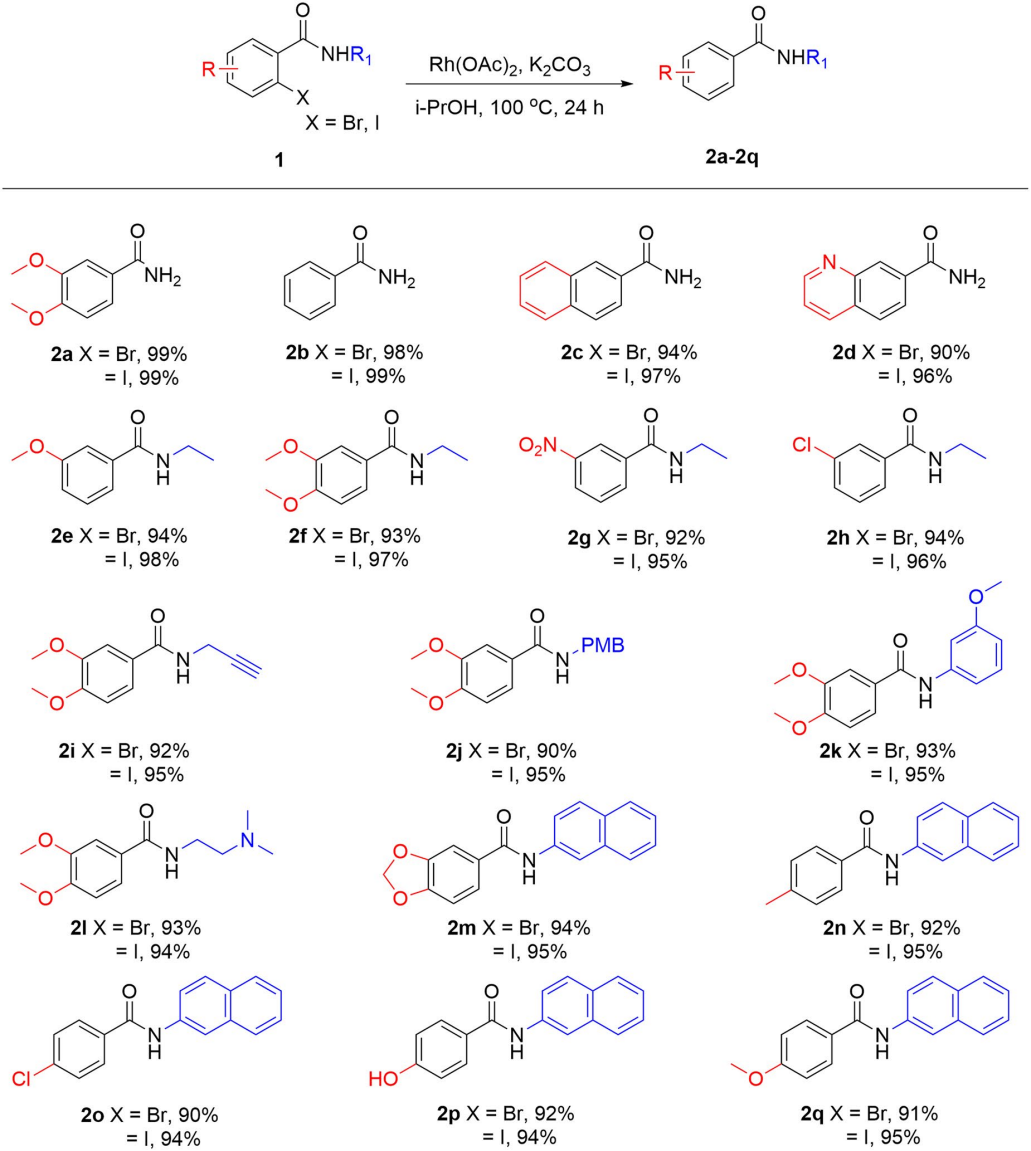
Scope of the hydrodehalogenation. Reaction conditions: **1** (1 equiv.), Rh_2_(OAc)_4_ (5 mol%), and K_2_CO_3_ (1.5 equiv.). Isopropanol (1 mL) stirred at 100 °C for 24 h.

Following the identification of optimal reaction conditions, the scope of the Rh-catalysed hydrodehalogenation was explored using a series of aryl halides with various electronic and steric properties (Figure 1). First, we surveyed the scope of 2-bromobenzamides bearing a wide range of functional groups on the phenyl ring. The introduction of electron-donating groups (–H, –OMe, –diOMe) or electron-withdrawing group (–NO_2_, –Cl), as well as replacement of the benzene ring with naphthalene or quinoline ring, was well tolerated, affording the corresponding products **2a–2h** in high yields ranging from 90% to 99%. Significantly, the yields of substitution with electron-withdrawing groups on the benzene ring were slightly lower than that of substitution with electron-donating groups. Next, the steric effect of the amide substituent on the transformation was investigated. Compared to the ethyl-substituted derivative, the bulky-substituents (alkyl chains and aromatic groups) including –PMB, –PhOMe, –CH_2_CH_2_N(CH_3_)_2_, and –naphthyl led to a slight reduction efficiency, affording products **2i–2q** in 90–95% yields. Finally, as we expected, the reduction of C–I bonds proceeded more efficiently than that of C–Br bonds for substrates **2a–2q**, providing 2-iodobenzamide derivatives in 94–99% yields.

**Scheme 2.**
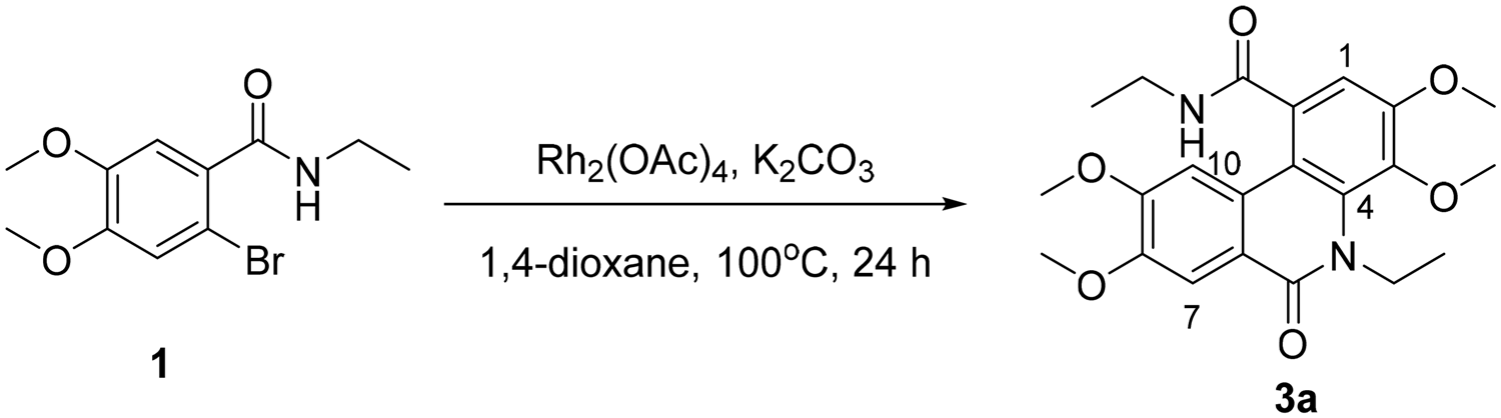
Rh-catalysed phenanthridinone construction with 2-halobenzamides (**1**)^a^. ^a^Reaction conditions: **1** (1 equiv.), Rh_2_(OAc)_4_ (5 mol%), and K_2_CO_3_ (1.5 equiv.). 1,4-Diooxane (1 mL) stirred at 100 °C for 24 h.

However, performing the reaction under the previously optimised hydrodehalogenation conditions in aprotic solvents failed to generate the expected products (Table 1). Instead, a phenanthridinone derivative (**3a**) was obtained under these conditions (Scheme 2).

**Figure 2.**
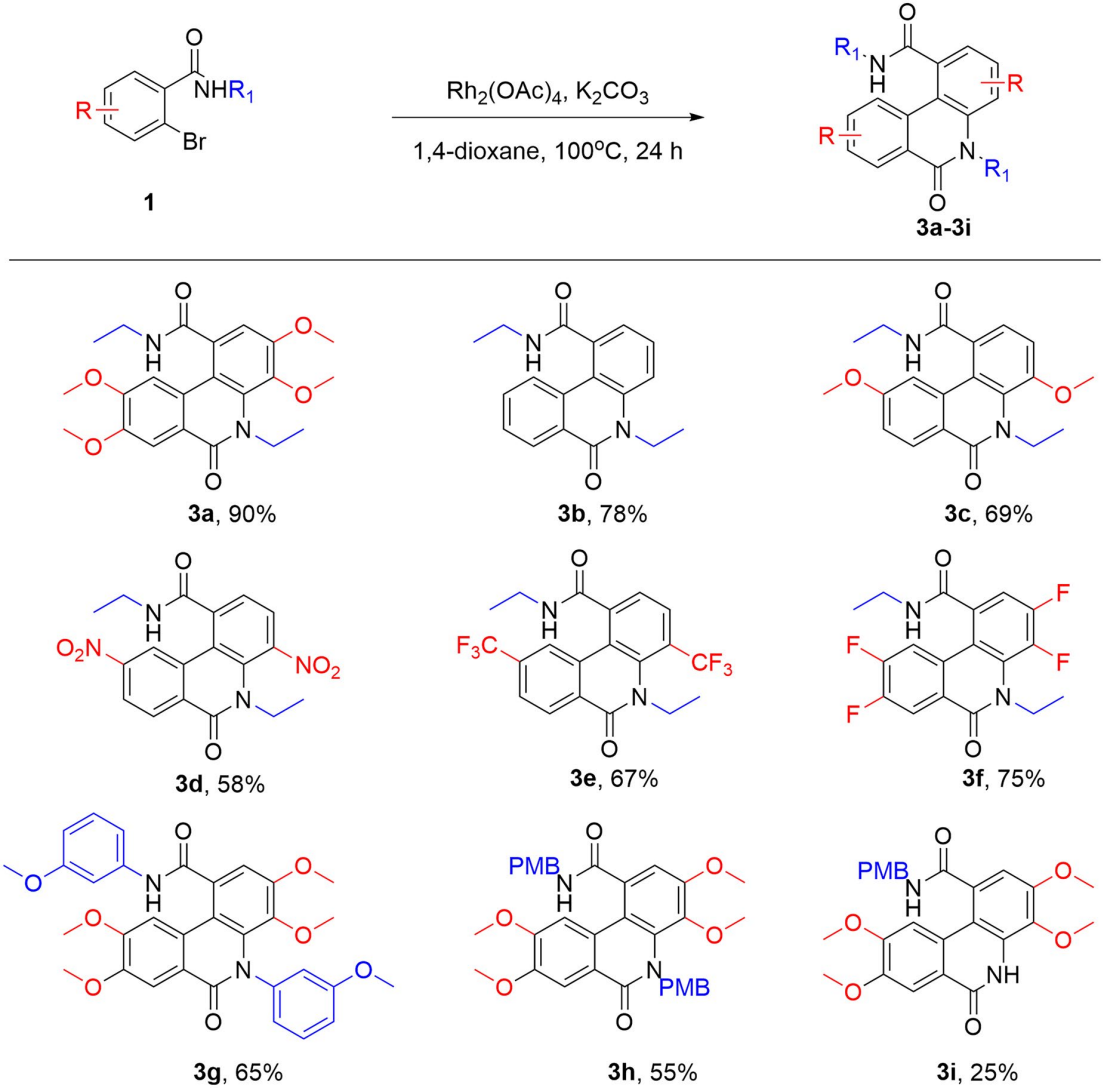
Scope of the coupling reactions. Reaction conditions: **1** (1 equiv.), Rh_2_(OAc)_4_ (5 mol%), and K_2_CO_3_ (1.5 equiv.). 1,4-Diooxane (1 mL) stirred at 100 °C for 24 h.

Inspired by the coupling reaction catalysed by Rh_2_(OAc)_4_, we subsequently focused our efforts on the Rh-catalysed construction of phenanthridinone skeletons from 2-halobenzamides. Thus, we further investigated the synthetic generality of the established protocol using 2-bromobenzamides **1** bearing various substituents (Figure 2). On one hand, reactions of 2-bromobenzamides substituted with electron-donating groups (–H, –OMe, –diOMe) and electron-withdrawing groups (–NO_2_, –CF_3_, –diF) on the aromatic ring all provided the desired annulation products **3a–3f** in moderate to high yields (58–90%). On the other hand, the scope of compatible *N*-substitutes was also evaluated. Notably, the yields of the target phenanthridinones decreased significantly when bulky aromatic substituents (–PMB, –PhOMe) were introduced (Figure 2, 3g**–3h**, 25–65%). That might be caused by the steric hindrance effect of the substituents, which interferes with the oxidative addition of the rhodium catalyst.

**Figure 3.**
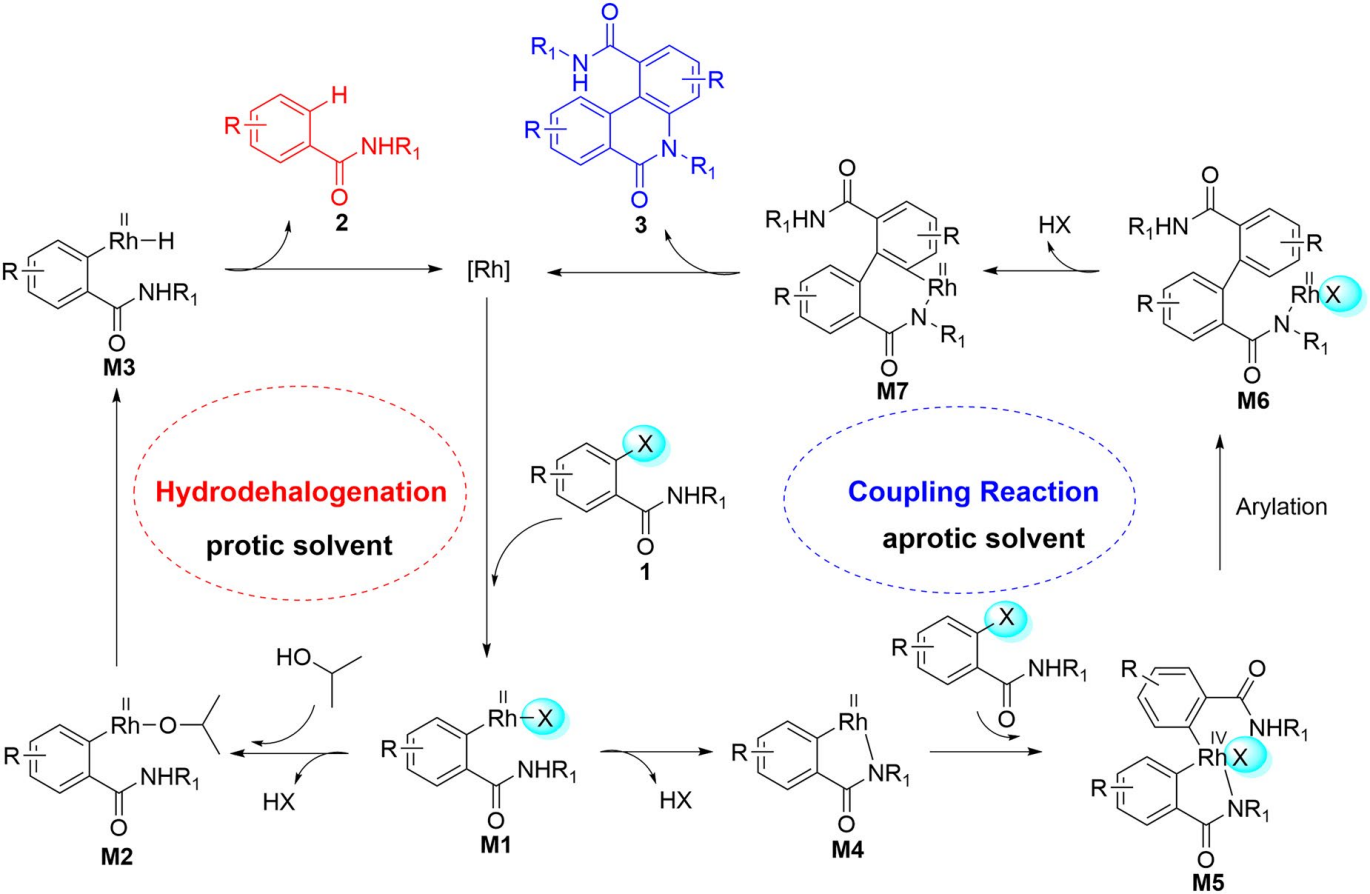
Proposed mechanism.

Based on the above experimental results and literature precedents^9^^,^^28^, a plausible mechanism for the Rh-catalysed hydrodehalogenation and coupling reaction is proposed (Figure 3). Both pathways proceed via a rhodium complex intermediate **M1** formed by oxidative addition of substrate **1**. Initially, *in situ* generated Rh(0) undergoes insertion into the C–X bond to give intermediate **M1**. Then, in the protic solvent such as isopropanol, an isopropyloxide ligand is introduced into intermediate **M2** under the mediation of a base additive. From the isopropyloxide intermediate **M2**, β-hydride elimination generates acetone and the Rh(II)–hydride complex **M3**, which releases the hydrodehalogenation product **2** and the active Rh(0) species after reductive elimination.

In the aprotic solvent, a five-membered Rh(II) rhodium cycle **M4** is generated from **M1** under basic conditions, followed by insertion into a second molecule of 2-bromobenzamide to form the Rh(IV) complex **M5**. This intermediate then undergoes biaryl bond formation to afford the Rh(II) complex **M6**. Finally, *N*-arylation proceeds with reductive elimination to release the coupling product **3**.

**Scheme 3.**
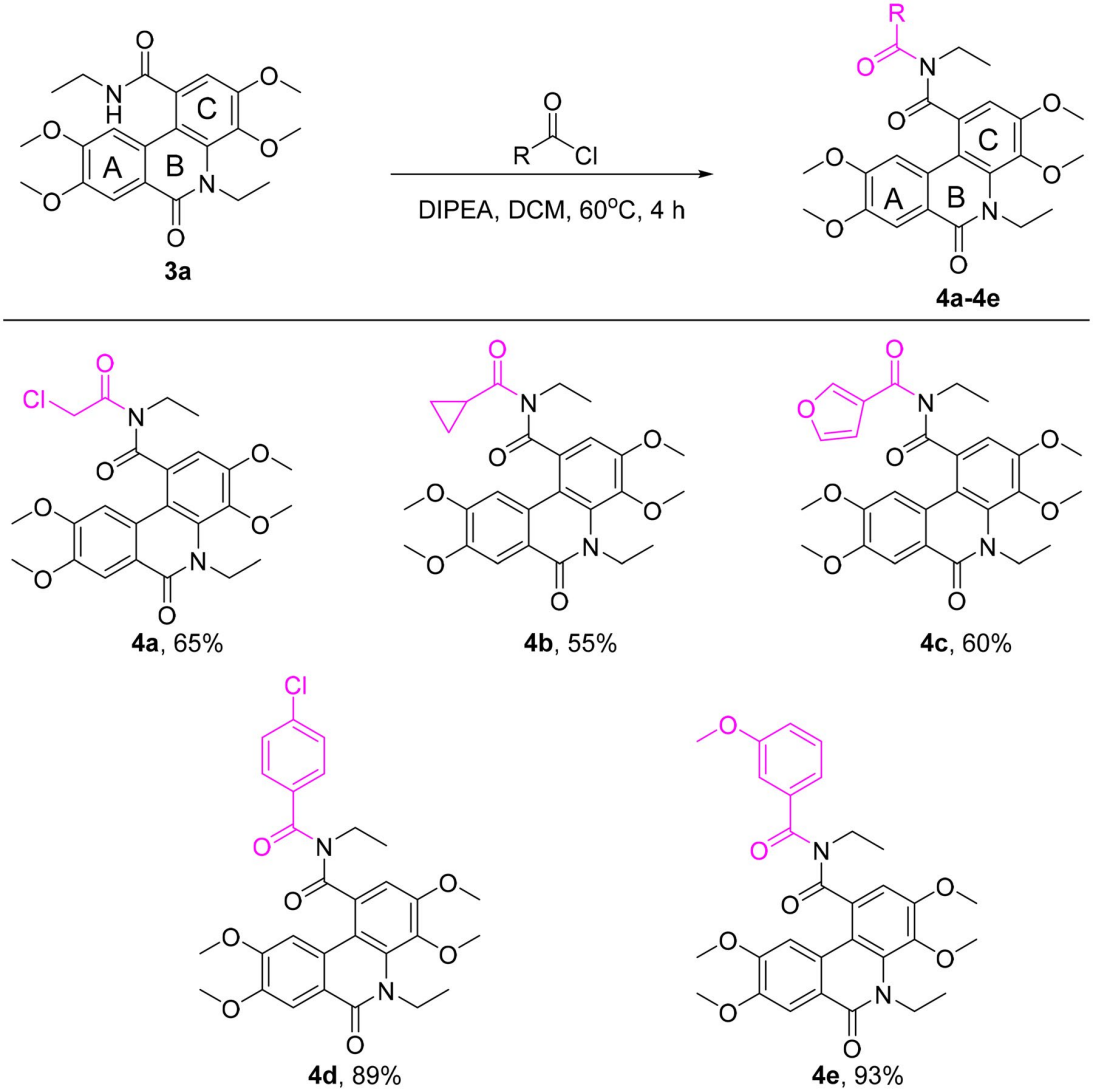
Synthesis of **4a**–**4e**. Reaction conditions: **3a** (1 equiv.), RCOCl (1.5 equiv.), and DIPEA (3 equiv.). DCM (3 mL) stirred at 60 °C for 4 h.

Next, we synthesised several phenanthridinone derivatives **4a–4e** from compound **3a** via nucleophilic substitution at the amide moiety of the C ring (Scheme 3). All synthesised compounds were characterised through nuclear magnetic resonance (NMR) spectra and high-resolution mass spectra (HRMS).

### TOP1 and TDP1 inhibition

As described previously, phenanthridinone skeletons represent an important class of heterocycles with potential as anticancer agents^29–33^. Such as oxynitidine, ARC-111 and PJ38 (Figure S1), which are used as inhibitors of topoisomerase IB (TOP1), tyrosyl-DNA phosphodiesterase 1 (TDP1), and poly(ADP-ribose) polymerase 1 (PARP1), respectively^19^^,^^34–38^. TOP1 regulates DNA topology, relieves DNA supercoiling during replication and transcription, and its inhibitors prevent the religation of the broken DNA stand, thereby inducing DNA double-strand breaks and cell death^39–41^. TDP1 specifically hydrolyses the 3′-phosphotyrosyl bond between nicked DNA and TOP1-derived peptides and various TDP1 inhibitors showed synergistic activities with topotecan (TPT) *in vivo*^42–46^. Therefore, we first tested the TOP1 and TDP1 inhibitory activities of all synthesised compounds. The TOP1 inhibition was assessed by a TOP1-mediated relaxation assay and the TDP1 inhibition was evaluated through a fluorescence assay as described previously^19^^,^^47^. As shown in Table 2, compound **3a**, a phenanthridinone scaffold with 2,3,8,9-tetraOMe substituent, exhibited potent TDP1 inhibitory activity but weak TOP1 inhibition (+; Figure S2). Conversely, compound **3c**, a phenanthridinone scaffold with 3,9-diOMe substituent, showed excellent TOP1 inhibitory activity of +++ and limited TDP1 inhibition (Table 2, Figure S2). Compound **3b**, with no methoxy substituted, showed low TOP1 inhibitory activity of + and no TDP1 inhibition. These results suggest that the number of methoxy substituents plays an important role in TOP1 and TDP1 inhibition. Compounds with 3,9-diNO_2_ (**3d**), 3,9-diCF_3_ (**3e**), and 2,3,8,9-tetraF (**3f**) groups, exhibited low or no TOP1/TDP1 inhibition. Similarly, bigger substituents at amides were unfavourable for TOP1 and TDP1 potency. Compounds with –PhOMe (**3g**) and –PMB (**3h** and **3i**) showed weak TOP1 and TDP1 inhibition. Even with the introduction of various amino chains at the amide position, the TOP1 and TDP1 inhibitory activity of derivatives (**4a–4e**) did not show any significant improvement.

**Table 2.** The TOP1 and TDP1 inhibition, and cytotoxicity of **3a–3i** and **4a**–**4e**.

Cpd	TOP1 inhibition^a^	TDP1 inhibition^b^	GI_50_ ± SD (μM)^c^			
			HCT-116	DU145	MCF-7	A549
**TPT**	++++	ND^d^	0.050 ± 0.016	0.21 ± 0.07	0.35 ± 0.04	0.31 ± 0.03
**NTD-212**	0	7.0 ± 1.4	31.06 ± 6.08	2.63 ± 0.16	5.98 ± 1.24	1.53 ± 0.07
**3a**	+	4.5 ± 0.2	11.27 ± 0.13	25.21 ± 0.06	14.49 ± 1.33	10.98 ± 0.95
**3b**	+	0	3.22 ± 0.19	5.39 ± 0.24	3.26 ± 0.42	2.61 ± 0.39
**3c**	+++	76.3 ± 1.4	0.24 ± 0.05	0.58 ± 0.18	1.01 ± 0.11	1.06 ± 0.08
**3d**	+	43.5 ± 3.5	0.92 ± 0.28	1.98 ± 0.25	1.20 ± 0.61	0.96 ± 0.08
**3e**	+	32.1 ± 3.7	1.06 ± 0.24	2.15 ± 0.66	1.67 ± 0.61	1.95 ± 0.84
**3f**	0	0	4.05 ± 1.02	3.53 ± 1.97	11.30 ± 5.94	15.14 ± 1.90
**3g**	+	0	12.18 ± 1.27	20.27 ± 0.96	14.67 ± 4.87	12.18 ± 1.54
**3h**	0	0	0.78 ± 0.11	15.57 ± 0.90	13.56 ± 1.88	2.75 ± 0.11
**3i**	0	0	2.18 ± 0.13	3.77 ± 0.02	9.71 ± 2.44	0.94 ± 0.06
**4a**	0	0	2.01 ± 0.11	9.94 ± 0.65	8.85 ± 1.15	0.79 ± 0.08
**4b**	0	23%	6.34 ± 0.88	17.38 ± 0.10	10.98 ± 0.71	12.31 ± 0.73
**4c**	0	0	4.77 ± 0.57	6.10 ± 1.74	5.20 ± 1.65	13.07 ± 2.82
**4d**	0	37%	2.90 ± 0.63	0.97 ± 0.52	1.61 ± 0.20	8.58 ± 0.55
**4e**	0	0	11.10 ± 0.27	2.04 ± 0.59	11.31 ± 2.65	5.53 ± 1.45

^a^ TOP1 inhibitory activity of the compounds was semi-quantitatively expressed relative to **TPT** at 100 μM as follows: 0, no inhibition; +, less than 29%; ++, between 30% and 59% activity; +++, between 60% and 89% activity; ++++, > 95% activity. **TPT** as the control compound.

^b^ TDP1 inhibition, determined by using a fluorescence assay, was expressed as the percentage inhibition (%) of the compounds at 100 μM concentration and the IC_50_ value (µM, mean ± SD). NTD-212^19^ as the positive control compound. Every experiment was repeated at least three times independently.

^c^ The cytotoxicity GI_50_ values were defined as the concentrations corresponding to 50% cell growth inhibition and obtained from MTT assay.

^d^ “ND” mean “not determined”.

**Figure 4.**
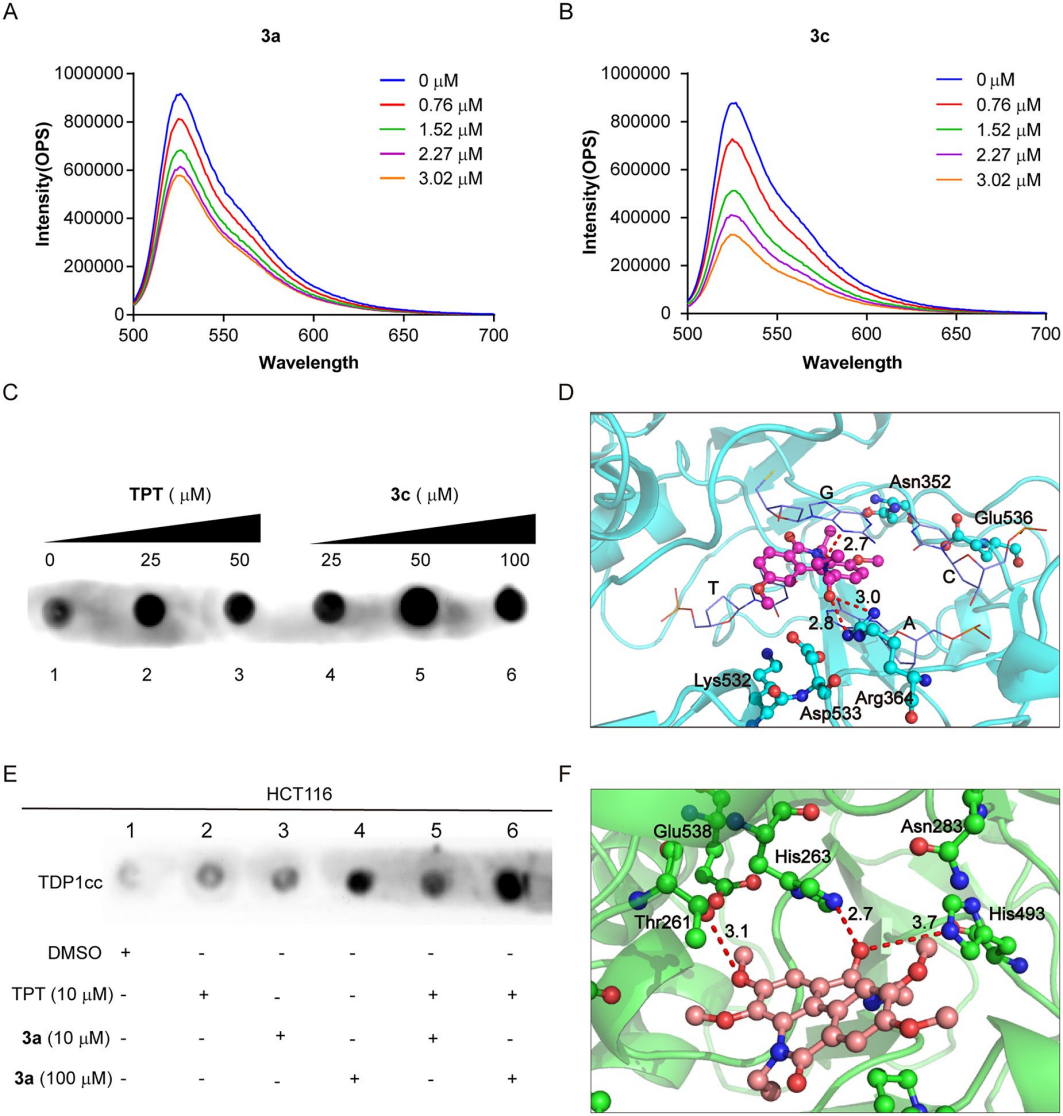
**3a** and **3c** interact respectively with TOP1 and TDP1. The interaction of **3a** and **3c** with DNA (A and B). (C) Stabilisation of TOP1 − DNA covalent cleavage complexes by ICE assay in HCT116 cells. Lane 1, untreated control; lanes 2 and 3, cells treated with TPT at 25 and 50 μM, respectively; lanes 4–6, cells treated with **3c** at 25, 50, and 100 μM, respectively. (D) Hypothetical binding mode of **3c** in the ternary TOP1 − DNA − drug cleavage complex (PDB: 1K4T). (E) Stabilisation of TDP1-DNA covalent complex by ICE assay in HCT116 cells. Lane 1, untreated control; lanes 2–4, cells treated with TPT (10 μM), and **3a** at 10 and 100 μM, respectively; lanes 5 and 6, cells co-treated with TPT (10 μM) and **3a** (10 and 100 μM), respectively. (F) Hypothetical binding mode of **3a** with TDP1 (PDB: 1RFF)^49^.

Subsequently, we hypothesised that the TOP1/TDP1 inhibition of the lead compounds (**3a** and **3c**) are associated with DNA interaction. To investigate this hypothesis, the interaction of **3a/c** with DNA was evaluated using the thiazole orange (TO) fluorescent probe. TO exhibits strong fluorescence at *λ*_527nm_ upon binding to DNA. Once interacting with DNA, compounds displace the TO probe from it, resulting in the decay of fluorescence^48^. As shown in Figure 4(A), compound **3a** reduced fluorescence intensity but exhibited no substantial further decrease with increasing concentration, indicating limited DNA binding capacity. In contrast, compound **3c** continuously reduced fluorescence intensity, implying stronger DNA binding affinity (Figure 4(B)).

Next, an immunocomplex of enzyme to DNA (ICE) assay was conducted in HCT116 cells to evaluate the ability of **3c** to stabilise cellular TOP1 cleavage complexes (TOP1cc). As shown in Figure 4(C), 3c induced cellular TOP1cc in a dose-dependent manner, similar to the positive control TPT, suggesting that TOP1cc is the cellular target of **3c**. A hypothetical binding mode was built by using *in silico* docking to evaluate the rationale for TOP1 inhibition^50^. The phenanthridinone scaffold of **3c** intercalated into the DNA break and formed a hydrogen bond (2.7 Å) between the amide nitrogen atom and guanine. Additionally, two strong hydrogen bonds (2.8 Å and 3.0 Å) between the amide oxygen atom and the side chain nitrogen of Arg 364 were observed, contributing significantly to TOP1 inhibition (Figure 4(D)). The limited space around Arg364 likely accounts for the decreased TOP1 inhibition observed for compounds with bulky amides substituent, consistent with the structure–activity relationship.

To assess whether **3a** stabilises induces the formation of cellular TDP1 cleavage complexes (TDP1cc), an ICE assay was also conducted in HCT116 cells according to our reported method^34^. As shown in Figure 4(E), TDP1cc was barely detectable in cells following incubation with TPT and **3a** at a low concentration (10 µM), but was significantly elevated upon co-incubated with TPT and **3a** at a high concentration (100 µM). These results imply that **3a** can target and stabilise cellular TDP1cc. Molecular modelling (Figure 4(F)) revealed that the skeleton of **3a** inserts into a large hydrophobic pocket adjacent to the DNA binding groove, with its 11-side chain extending towards the narrow catalytic centre of TDP1 and forming two hydrogen bonds with His263 (2.7 Å) and His493 (3.7 Å). A hydrogen bond was also observed between the oxygen atom of 2-methoxy group and Thr261 (3.1 Å), which may contribute to TDP1 inhibition.

### Cytotoxicity assays

The cytotoxicity of the synthesised compounds was evaluated using the MTT (3-(4,5-dimethylthiazol-2-yl)-2,5-diphenyl tetrazolium bromide) assay against four human tumour cell lines: colon cancer (HCT116), prostate cancer (DU-145), breast cancer (MCF-7), and non-small cell lung cancer (A549). Cells were treated for 72 h in a five-dose assay with concentrations ranging from 0.01 to 100 μM. As shown in Table 2 and Figure S3, compounds with low TOP1 inhibitory activity showed cytotoxicity with GI_50_ values in the low micromolar range, except for compound **3d** and **3h** with high cytotoxicity at sub-micromolar level (**3d**: 0.92 μM for HCT116 and **3h**: 0.78 μM for HCT116). Compound **3a**, which exhibited potent TDP1 inhibitory activity, showed low cytotoxicity against the four cancer cell lines. Conversely, compound **3c** displayed marked cytotoxicity against the four cell lines, consistent with its strong TOP1 inhibition potency.

**Figure 5.**
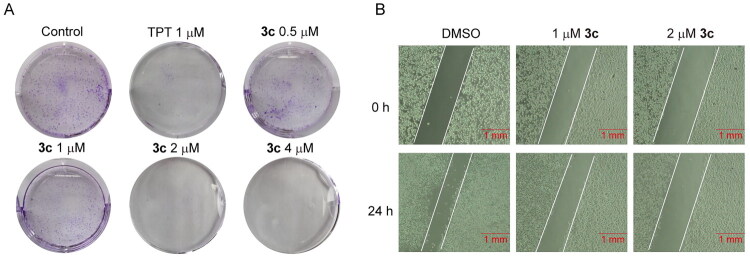
**3c** inhibits the proliferation and migration of colorectal cancer cells. (A) The representative images of colony formation assay of TPT (1 μM) and **3c** (0.5, 1, 2, and 4 μM) in HCT116 cells. (B) The results of wound healing assay treated with **3c** (1 and 2 μM) in HCT116 cells.

To further evaluate the antitumor activity of **3c**, the cell proliferation and migration were tested by colony formation and wound healing assays in HCT116 cells, with TPT as a positive control^51^. Similar to TPT, **3c** inhibited the cell proliferation in a dose-dependent manner (Figure 5(A)). Wound healing assay results also indicated that **3c** effectively suppressed the migration of HCT116 cells (Figure 5(B)).

The cytotoxicity of **3c** was further evaluated in drug-resistant HCT116 cells (SiTOP1-HCT116) as reported previously^19^. The SiTOP1-HCT116 cell line was developed by transfection of colon cancer parental cell line (HCT116) with short hairpin RNA vectors expressing siRNA for TOP1. As shown in Table 3 and Figure S4, compared with the parental HCT116 cell line, SiTOP1-HCT116 cells showed significant resistance to TPT (10.4-fold) and **NTD-96**^19^ (14.2-fold), as well as to **3c** (12.2-fold), indicating that TOP1 is a major cellular target of **3c**. However, a slight proliferative stimulation effect of compound **3c** in SiTOP1-HCT116 cells was observed at low concentrations. It might be attributed to the hormetic effect which is characterised by a mild growth-promoting response at low doses and a significant cytotoxic effect at elevated concentrations.

**Table 3.** Cytotoxicity of **3c** in drug-resistant HCT116 cells.

Cpd	GI_50_ ± SD (μM)^a^		
	HCT116	SiTOP1-HCT116	Resistance index^b^
**TPT**	0.05 ± 0.02	0.52 ± 0.05	10.4
**NTD-96**	0.14 ± 0.02	2.01 ± 0.26	14.2
**3c**	0.24 ± 0.05	2.92 ± 0.71	12.2

^a^ GI_50_ values (means ± SD) were defined as the concentrations of compounds that resulted in 50% cell growth inhibition and obtained from MTT assay. Every experiment was repeated at least three times.

^b^ Resistance index was calculated by dividing the GI_50_ of SiTOP1-HCT116 cells by the GI_50_ of HCT116 cells.

### Compound 3a enhances chemoradiotherapy in HCT116 cells

**Figure 6.**
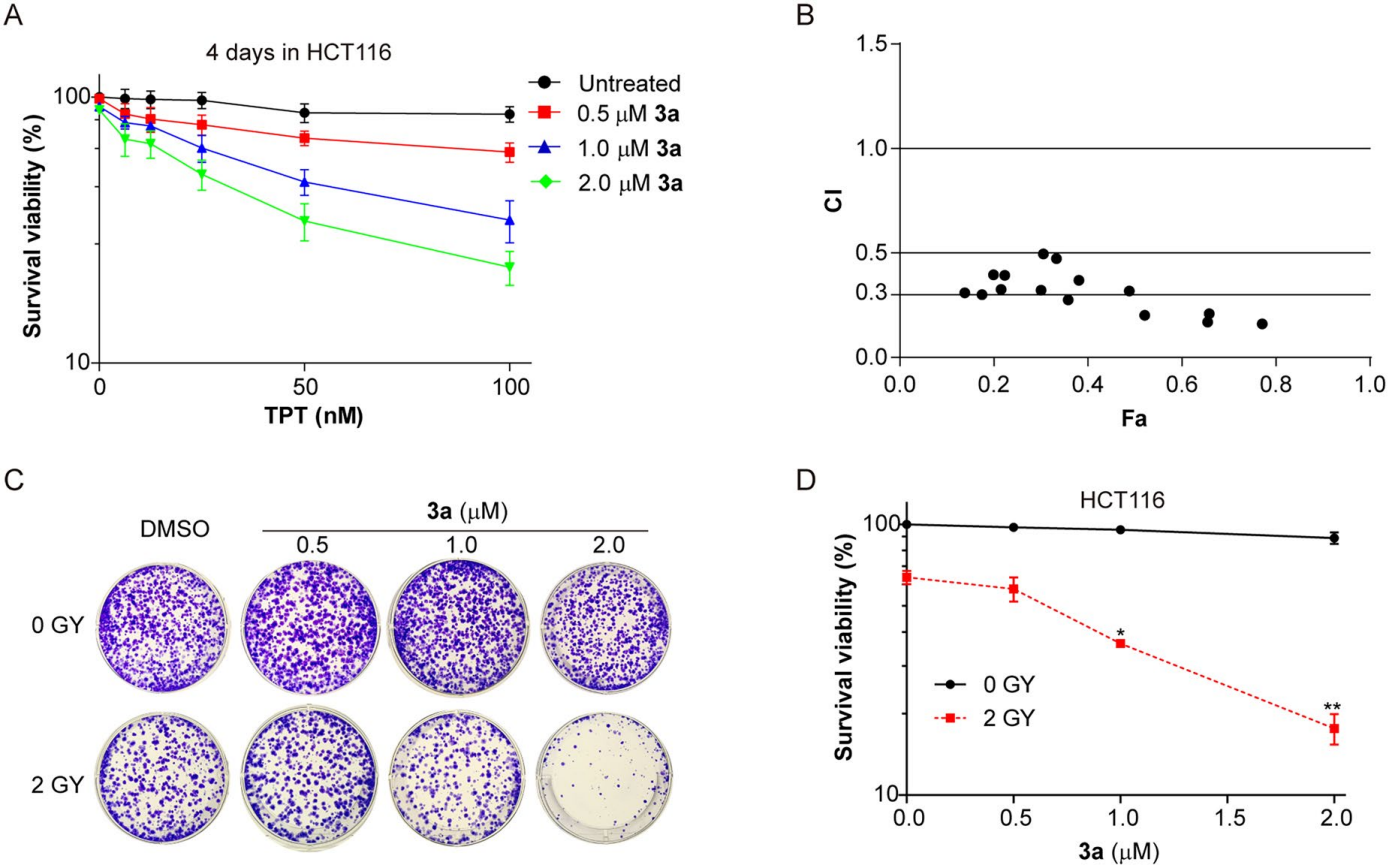
**3a** enhances chemoradiotherapy in HCT116 cells. Synergistic effect (A) and combination index (B) of **3a** with TPT in HCT116 cells. The cells were co-incubated for 96 h. (C) The representative images (C) and results (D) of colony formation assay of **3a** combined with IR (2 Gy) in HCT116 cells. Data are presented as the mean ± SD (*n* = 3). *p < 0.05, **p < 0.01.

TDP1 inhibitors were reported to exhibit synergistic effects with TOP1 inhibitors and ionising radiation (IR) in tumour cells^19^^,^^34^^,^^52^. To assess whether our TDP1 inhibitor **3a** could act synergistically with TOP1 inhibitors and IR, the combined effects of **3a** with TPT and IR were tested in HCT116 cells. Following 96 h of incubation at 37 °C, the cytotoxicity of TPT against HCT116 cells was significantly enhanced by co-incubation with **3a** in a dose-dependent manner (Figure 6(A)**)**. At a concentration of 100 nM, approximately, 14% of cells were killed by TPT alone and about 77% of cells were killed by co-incubation with 2 μM **3a**. Combination index (CI) value is calculated based on the median effect principle, where a CI value <1 indicates a synergistic effect, CI = 1 indicates an additive effect, and CI >1 indicates an antagonistic effect. As shown in Figure 6(B), the CI values for the combination of TPT and **3a** fell within the range of 0.1–0.5, with five doses showing strong synergistic effects (CI < 0.3). Further studies demonstrated that **3a** also exerted a potent radiosensitising effect in HCT116 cells in a dose-dependent manner. About 32% of cells were killed by IR (2 Gy) alone, whereas the combination of IR (2 Gy) with 2.0 μM **3a** resulted in about 92% cell death (Figure 6(C,D)). Collectively, these results suggested that the phenanthridinone derivative **3a** showed synergistic effect with TOP1 inhibitor TPT and radiosensitising effect in HCT116 cells.

### DNA damage and apoptosis induced by compound 3c

**Figure 7.**
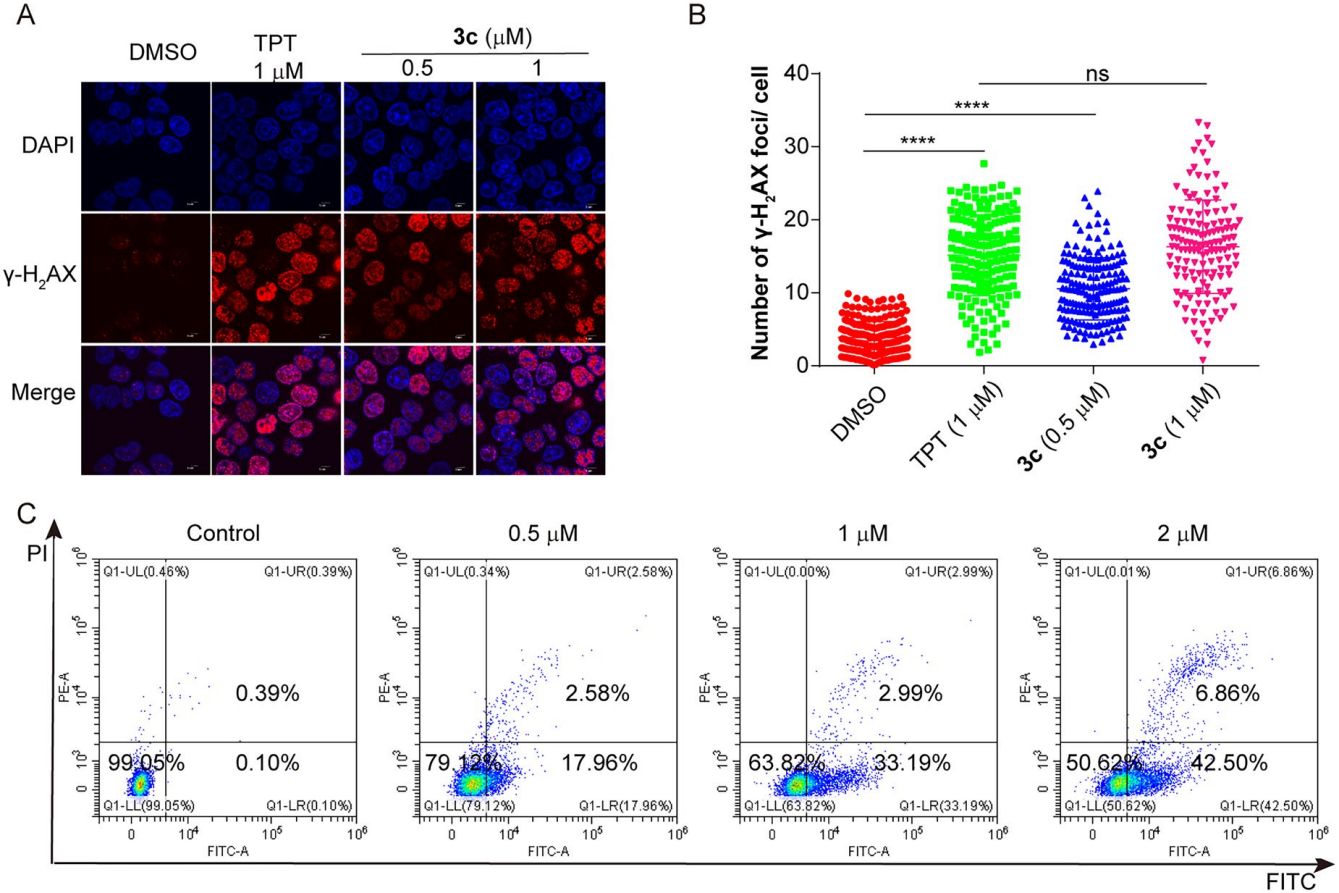
DNA damage and apoptosis induced by **3c**. (A) γ-H_2_AX foci induced by TPT (1 μM) and **3c** (0.5 and 1 μM) in HCT116 cells. (B) Quantitative analysis for the level of γ-H_2_AX foci. (C) *In vitro* apoptosis-inducing effects in HCT116 cells of **3c** (0.5, 1, and 2 μM) by flow cytometry. ****p < 0.0001.

To evaluate DNA damage induced by the phenanthridinone derivative **3c** in cancer cells, drug-induced γ-H_2_AX (phosphorylated histone H2AX) foci formation were tested by immunofluorescence assay in HCT116 cells. Compound **3c** induced γ-H_2_AX foci in a dose-dependent manner following 3 h of incubation (Figure 7(A,B)). The number of γ-H_2_AX foci induced by 1 μM **3c** was equivalent to that induced by 1 μM TPT, indicating that **3c** effectively induces cellular DNA damage, likely attributable to its TOP1 inhibitory activity and the consequent trapping of cellular TOP1cc.

A flow cytometry assay was performed to assess the induction of cells apoptosis by **3c**. After incubation with **3c** for 24 h, apoptotic HCT116 cells were detected and **3c** induced apoptosis in a dose-dependent manner (Figure 7(C)). Approximately, 49.36% of cells (42.50% early apoptotic cells and 6.86% late apoptotic cells) were scored as apoptotic after 24 h of treatment with 2 μM **3c**.

### Antitumor activity of compound 3c *in vivo*

**Figure 8.**
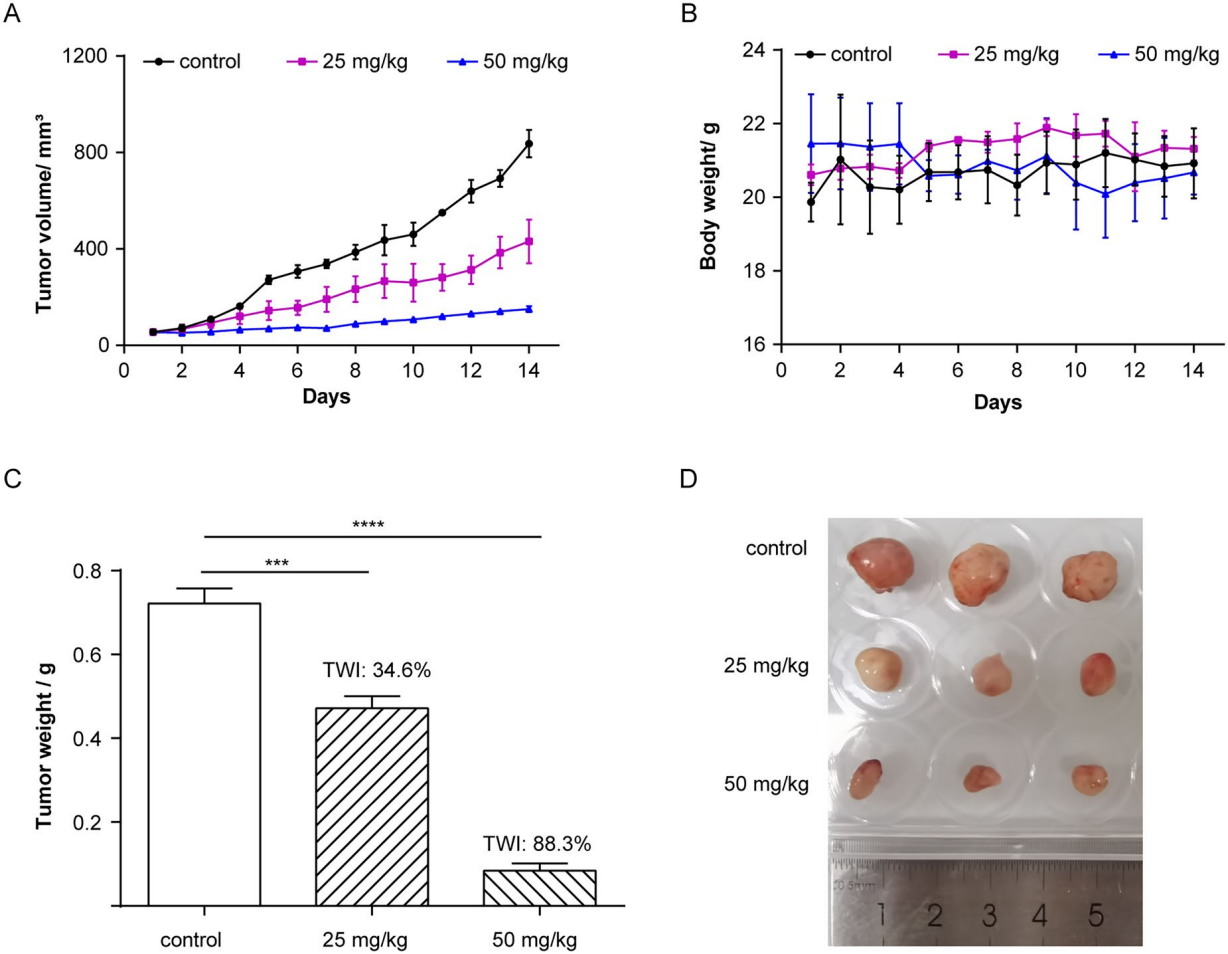
The antitumor activity of **3c** *in vivo*. (A) Tumour growth curves after the intraperitoneal injection of **3c**. The effects of **3c** on tumour volume (A), body weight (B), and tumour weight (C) at the dose of 25 and 50 mg/kg. (D) Tumour tissues excised from euthanised HCT116 tumour-bearing mice after applying drugs for 14 days. Data are presented as the mean ± SD (*n* = 3). ***p < 0.001, ****p < 0.0001.

To test the antitumor activity of **3c** *in vivo*, nude mice bearing HCT116 xenografts were randomly divided into three groups (*n* = 3) and administered daily with saline, 25 mg/kg **3c** and 50 mg/kg **3c** by intraperitoneal (ip) injection. Treatment with **3c** significantly reduced tumour volume in a dose-dependent manner (Figure 8(A)). Moreover, mice treated with **3c** exhibited no significant body weight loss compared with the control group (Figure 8(B)). Tumour weights in the groups treated with 25 mg/kg and 50 mg/kg **3c** were reduced by 34.6% and 88.3%, respectively (Figure 8(C,D)). Furthermore, no obvious organ toxicity was observed in the heart, liver, spleen, lung, or kidney among the three groups (Figure S5).

## Conclusions

In summary, we have successfully developed a simple and highly efficient strategy for the selective synthesis of either dehalogenation products or phenanthridinone skeletons from a common precursor. This protocol employs a cheap and commercially available Rh_2_(OAc)_4_ catalyst without additional ligands, enables scalable synthesis with low catalyst loading, and tolerates a wide range of 2-halobenzamide substrates. Further applications of this method in natural-product derivatives synthesis are currently underway.

A series of phenanthridinone derivatives were synthesised and evaluated for their TOP1 and TDP1 inhibitory activities, as well as cytotoxicity against four human cancer cell lines (HCT116, MCF-7, DU-145, and A549 cell lines). Enzymatic and cellular assays revealed that phenanthridinone derivative **3a** showed strong TDP1 inhibitory activity and low cytotoxicity against all four cancer cell lines. Compound **3a** induced cellular TDP1cc in ICE assays when combined with TPT and showed strong synergistic effects in combination with both TPT and IR in HCT116 cells. Conversely, compound **3c** effectively trapped TOP1cc and exhibited potent antiproliferative activity (GI_50_: 0.24 μM for HCT116, 0.58 μM for DU145, 1.01 μM for MCF-7, and 1.06 μM for A549). Compound **3c** also suppressed the migration of HCT116 cells and exhibited a decrease of antitumor activity in drug-resistant HCT116 cells. Additionally, **3c** induced DNA damage and apoptosis in HCT116 cells and showed high antitumor efficacy *in vivo*. In conclusion, these findings suggest that phenanthridinone derivatives deserve to be further investigated and potentially developed in reversing tumour resistance and radiosensitising effect.

## Experimental

### Synthesis and characterisation

All chemicals and solvents were obtained from local commercial suppliers and utilised directly without further purification unless otherwise specified. Melting points were measured using an X-5 micro melting point apparatus with samples mounted on glass slides. Nuclear magnetic resonance spectra were acquired on Bruker BioSpin GmbH spectrometer (Billerica, MA) at 400 or 500 MHz, with tetramethylsilane (TMS) as the internal reference standard. High-resolution mass spectra were recorded using a Shimadzu LCMS-IT-TOF mass spectrometer (Kyoto, Japan). All synthesised compounds subjected to biological activities testing should have a purity of more than 95% following purification by column chromatograph with silica gel (200–300 mesh, Qingdao Haiyang Chemical Co. Ltd., Qingdao, China). The purities were analysed by high-performance liquid chromatography (HPLC) and the analysis conditions were as follows: detection wavelength, 220 nm, flow rate, 1.0 mL/min, linear gradient from 55% PBS buffer (pH 3) and 45% MeOH to 15% PBS buffer (pH 3) and 85% MeOH over 30 min.

### Synthesis of compound 2 (general procedure)

Rh_2_(OAc)_4_ (5 mol%) and K_2_CO_3_ (0.3 mmol, 1.5 equiv.) were added to the solution of **1** (0.2 mmol, 1 equiv.) in isopropyl (1 mL) and then stirred at 100 °C for 24 h. After being cooled to room temperature, the resulting solution was concentrated under reduced pressure. The residue was dissolved with ethyl acetate (5 mL), washed with saturated saline (5 mL × 1), dried (MgSO_4_) and concentrated under reduced pressure to give the target compounds without any further purification.

### Synthesis of compound 3 (general procedure)

To the solution of **1** (0.2 mmol, 1 equiv.) in 1,4-dioxane (1 mL), Rh_2_(OAc)_4_ (5 mol%) and K_2_CO_3_ (0.3 mmol, 1.5 equiv.) were added, and then stirred at 100 °C for 24 h. The resulting solution was extracted with ethyl acetate (3 × 5 mL) after being cooled to room temperature. The combined organic layer was washed with saturated saline (10 mL × 1), dried (MgSO_4_) and concentrated under reduced pressure. The residue was purified by silica gel column chromatography using dichloromethane/methanol (30:1) as eluent to give the target products.

### Synthesis of compounds 4a–4e (general procedure)

To the solution of compound **3a** (0.2 mmol, 82 mg) in dry DCM (2 mL), a solution of chloride material (0.3 mmol) in dry DCM (1 mL) was added dropwise. Then, DIPEA (0.4 mmol) was added dropwise and the reaction solution was stirred at 60 °C for 4 h. The resulting solution was diluted with DCM (5 mL) and washed with saturated saline (5 mL × 2), dried (MgSO_4_) and concentrated under reduced pressure. The residue was purified by silica gel column chromatography using dichloromethane/methanol (20:1) as eluent to give the target compounds **4a–4e**.

### TOP1 inhibition assay

All compounds were evaluated for TOP1 inhibitory activity using a TOP1-mediated relaxation assay. Briefly, A 20 μL of reaction mixture containing 1 μL of supercoiled pBR322 DNA (0.5 μg/μL) prepared in relaxation buffer (10 mM Tris–HCl, pH 7.5, 15 μg/mL BSA, 5 mM MgCl_2_, 50 mM KCl, 40 μg/mL DTT) was incubated with 1 unit of calf thymus TOP1 at 37 °C for 30 min, either with or without the tested compound. Then, 4 μL of 6 × loading buffer was added and the mixture was analysed by electrophoresis on a 0.8% agarose gel in TBE buffer at 4.6 V/cm for 1.5 h. The gel was stained with 1 × gel red for 30 min and visualised under UV transillumination.

### TDP1 inhibition assay

A linear oligonucleotide labelled with FAM (6-carboxyfluorescein) and BHQ (Black Hole Quencher), 5′-FAMAGGATCTAAAAGACTT-BHQ-3′ was employed as a quenched fluorescent substrate. TDP1 solution (20 μL/well, 0.02 μL of purified TDP1 (100 nM) in 10 mM Tris–HCl, pH 7.5, 1 mM EDTA, 50 mM KCl, 1 mM DTT) was dispensed into wells of a white 384-well plate (NEST). Five microlitres of the tested compound solution in DMSO was added to assay plates and incubated at room temperature for 30 min. Meanwhile, the plates were read using a Flash multimode reader (Molecular Devices, San Jose, CA) at Ex485/Em510 nm to identify false-positive compounds with autofluorescence. The reaction was initiated by adding 25 μL of the linear oligonucleotide substrate (35 nM) into each well. The plate was immediately read five times using a kinetic read on the same Flash multimode reader. TDP1 percentage inhibition of the tested compounds was calculated by comparing the fluorescence increase rate in compound-treated wells with that in DMSO control wells over time.

### Cell culture and MTT assay

Human colorectal cancer cell lines (HCT116), prostate cancer cell line (DU145), breast cancer cell line (MCF-7), and lung cancer cell line (A549) were purchased from Laboratory Animal Center of Sun Yat-sen University (Guangzhou, China). Cancer cells were cultured in GIBCO RPMI 1640 medium (Waltham, MA) or DMEM complemented with 10% foetal bovine serum (FBS) at 37 °C with 5% CO_2_. Cells were treated with compounds and collected for various times in different experiments.

For cytotoxicity assessment, cells were treated with the tested compounds (pre-dissolved in DMSO) at a five-dose assay ranging from 0.01 to 100 μM (0.001–10 μM for TPT) concentration as reported previously^19^. After incubation for 72 h at 37 °C, MTT solution (50 μL, 1 mg/mL) in PBS (PBS without MTT as the blank) was fed to each well. The formazan crystal formed in the well was dissolved with 100 mL of DMSO for optical density reading at 570 nm after 4 h incubation. The percent inhibition of cell viability was calculated relative to the vehicle control, and the GI_50_ values were determined by nonlinear regression analysis using GraphPad Prism software (San Diego, CA).

For drug combination experiment, HCT116 cells were incubated with TPT and the tested compounds for 96 h at 37 °C, after which cell viability was assessed by MTT assay.

For colony formation assay, HCT116 cells were treated with the tested compounds alone or with IR using an X-ray linear accelerator (Rad Source, RS2000Lite, Buford, GA) and cultured for 10–14 days. Then, the treated cells were rinsed with PBS, fixed in methanol and stained with crystal violet. Colonies containing more than 50 cells were counted as surviving clones and normalised to the plating efficiency of sham-treated control cells.

### Fluorescence spectroscopy

The concentrations of TO and ctDNA were 2 μM and 4 μM, respectively, while compound concentrations ranged from 0 to 3.96 μM for competition binding experiments. Excitation was performed at 488 nm and emission spectra were recorded from 500 to 700 nm by Fluoromax-4 (HORIBA, Irvine, CA).

### Cell migration assay

HCT116 cells were plated 5 × 10^6^ cells per well in six-well plates. Upon formation of a confluent monolayer, a sterile 200 μL pipette tip was used to create a vertical scratch wound across the cell layer. The floating cells and debris were removed by washing with PBS, and the remaining cells were incubated with **3c** for 24 h. The wound closure was imaged at 0 and 24 h using an inverted FV3000 microscope (Olympus, Tokyo, Japan).

### Immunodetection of cellular TOP1cc and TDP1cc

The ICE assays were performed according to previously reported methods^19^^,^^34^. Briefly, after treated with the tested compounds at the indicated concentrations for 1 h, HCT116 cells were lysed with DNAzol reagent (1 mL) at 25 °C for 30 min. The genomic DNA was harvested by centrifugation and dissolved in 0.2 mL of 8 mM NaOH solution. Two micrograms of DNA was resuspended in 30 μL of 25 mM NaH_2_PO_4_ buffer and loaded onto nitrocellulose membranes. The membranes were incubated with rabbit monoclonal antibodies against human TOP1 (Abcam, Cambridge, UK, 1:1000) or TDP1 (Abcam, Cambridge, UK, 1:1000) at 4 °C overnight, followed by incubation with HRP conjugated secondary antibodies (Cell Signaling Technology, Danvers, MA, 1:3000) at room temperature for 1 h. Immunoreactive dots were detected using Immobilon Western Chemiluminescent HRP substrate (Millipore, Burlington, MA).

### Molecular modelling

Binding models of TOP1 and TDP1 with DNA and the tested compounds were generated by the Molecular Operating Environment (MOE) software following protocols described in previous studies^34^. Briefly, the X-ray crystal structures of TOP1–DNA–ligand complex (PDB ID: 1K4T)^50^ and TDP1–DNA–vanadate complex (PDB ID: 1RFF)^49^ were obtained and cleaned and inspected for errors and missing residues, hydrogens were added, and the water molecules and the ligand were deleted. The ternary complex ligand centroid coordinates for docking were defined using the ligand in the complex structure as the centre of the binding pocket. Compounds were constructed and optimised using ChemDraw and saved in SDF file formats and minimised by the conjugate gradient method using the MMFF94x force field. Induced fit was used for docking with the default parameters. The top 20 docking poses per ligand were visually inspected following docking calculations and the selected poses were further subjected to energy minimisations using the AMBER force field.

### Immunofluorescence assay

Immunofluorescence staining and confocal microscopy were performed to detect γ-H_2_AX foci formation. HCT116 cells were exposed to the tested compounds at 37 °C for 3 h, followed by fixation with 4% paraformaldehyde for 10 min. The treated cells were permeabilised with 0.5% Triton X-100 in PBS at 37 °C for 30 min and blocked with 5% goat serum in PBS at 37 °C for 3 h. After incubation with γ-H_2_AX primary antibody (Abcam, Cambridge, UK, 1:400) at 4 °C overnight, the coverslips were washed six times with blocking buffer and incubated with anti-rabbit Alexa 488-conjugated antibody (Invitrogen, Carlsbad, CA, 1: 1000) along with 2 μg/mL DAPI (4,6-diamidino-2-phenylindole) (Invitrogen, Carlsbad, CA). The digital images were recorded using an FV3000 microscope and analysed with FV31S-SW software.

### Apoptosis detection

Apoptosis was detected using the FITC Annexin V/PI Apoptosis Detection Kit (KeyGEN, Nanjing, China). HCT116 cells were planted and treated with the tested compounds for 24 h. The harvested cells were resuspended in 500 μL of binding buffer, followed by the addition of 5 μL FITC Annexin V and PI staining reagents. The samples were gently mixed and incubated at 25 °C for 15 min in the dark. Finally, the fluorescence-positive cells were quantified by flow cytometry (EPICS XL).

### Antitumor activity *in vivo*

Tumorigenesis in nude mice can recapitulate the growth process of human tumours *in vivo* and is widely used to develop and evaluate the efficacy and safety of anticancer drug candidates. Thus, we evaluated the anticancer effect of compound **3c** by xenograft models in nude mice. All animals were purchased from the Laboratory Animal Center of Sun Yat-sen University (Guangzhou, China) and treated according to the experimental procedures approved by the Institutional Animal Care and Use Committee of Sun Yat-sen University (Approval No. SYSU-IACUC-2023-B0666). All authors have adhered to the ARRIVE guidelines (https://arriveguidelines.org/). Nine male BALB/c nude mice (4–5 weeks, 15–18 g) were randomly divided into three groups (*n* = 3) for the establishment of xenograft models. Before the experiments, all mice were given a week to adjust to the clean, hygienic and specific-pathogen-free (SPF) environment. All nude mice were taken care of in stainless steel cages with 60% humidity, 25 ± 1 °C, and a 12/12 light/dark cycle. All efforts were made to minimise animal suffering: mice were housed in standard cages to reduce stress, and routine health checks were performed to monitor for signs of discomfort. Nude mice were fed with refreshing water and nourishing food daily. The nude mice were anaesthetised with 50 mg/kg pentobarbital sodium (dissolved in saline) and then tumour fragments (approximately 5 mm^3^) harvested from HCT116 xenografts were subcutaneously implanted into the right flank of the nude mice. When the tumour volume grew to nearly 100 mm^3^, the mice in the three groups were treated with saline, 25 mg/kg **3c** and 50 mg/kg **3c** (dissolved in saline), respectively, once daily by ip injection for 14 days. Tumour volume was calculated according to the following formula: *V* = length × width × width/2. The diameter of the tumour in mice did not exceed 15 mm and the tumour volume did not exceed 2000 mm^3^. Upon completion of the experiment, all mice were euthanised by cervical dislocation. Death was confirmed by non-breathing and pupil enlargement. Tumours were excised, weighed, and sliced for further pathological analysis.

## Supplementary Material

Support_information_marked_Cl.docx

## Data Availability

Data will be made available on request.
