# Supplementary material for: Solvent-controlled Rh-catalysed hydrodehalogenation and construction of phenanthridinone skeleton from a common precursor in one-step sequence and the antitumor activity of its derivatives
Source: J Enzyme Inhib Med Chem. 2026 Jul 28;41(1):2704432. doi: 10.1080/14756366.2026.2704432 (PMC13417644; doi:10.1080/14756366.2026.2704432)
Supplement: Support_information_marked_Cl.docx [file IENZ_A_2704432_SM0546.docx]

**Supporting Information**

Solvent-Controlled Rh-catalyzed Hydrodehalogenation or construction of phenanthridinone skeleton from a common precursor in one-step sequence and the Antitumor Activity of its derivatives.

**Figure S1.** Alkaloids containing phenanthridinone skeletons.


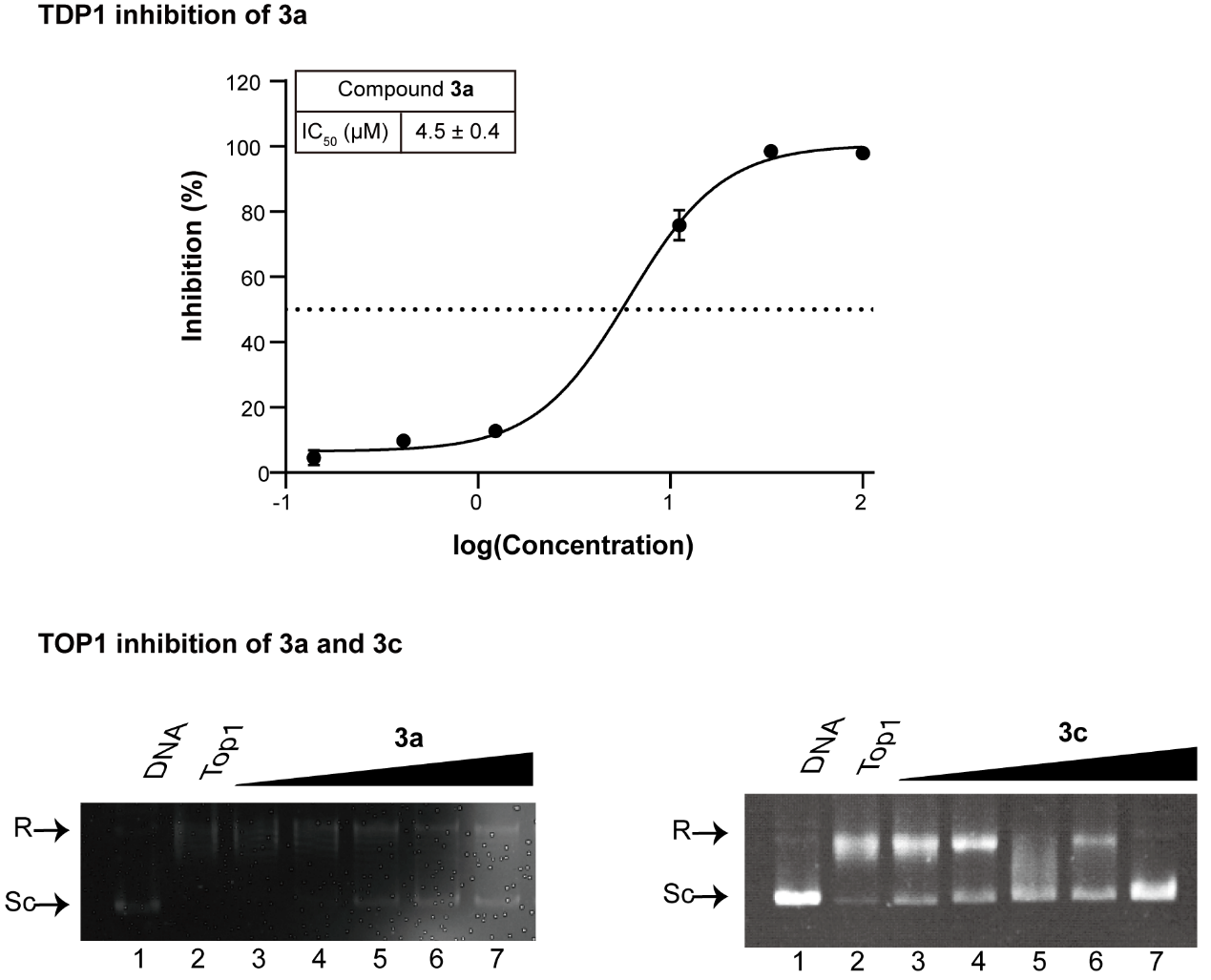


**Figure S2.** TOP1 and TDP1 inhibition of lead compounds **3a** and **3c**. Up: the TDP1 inhibition curves of compound **3a**. Down: TOP1-mediated relaxation assays. Lane 1, pBR322 DNA alone; lane 2, pBR322 DNA and TOP1; lanes 3–7, pBR322 DNA, TOP1 and **3c** at 0.2, 1, 5, 25, 125 μM concentrations, respectively.
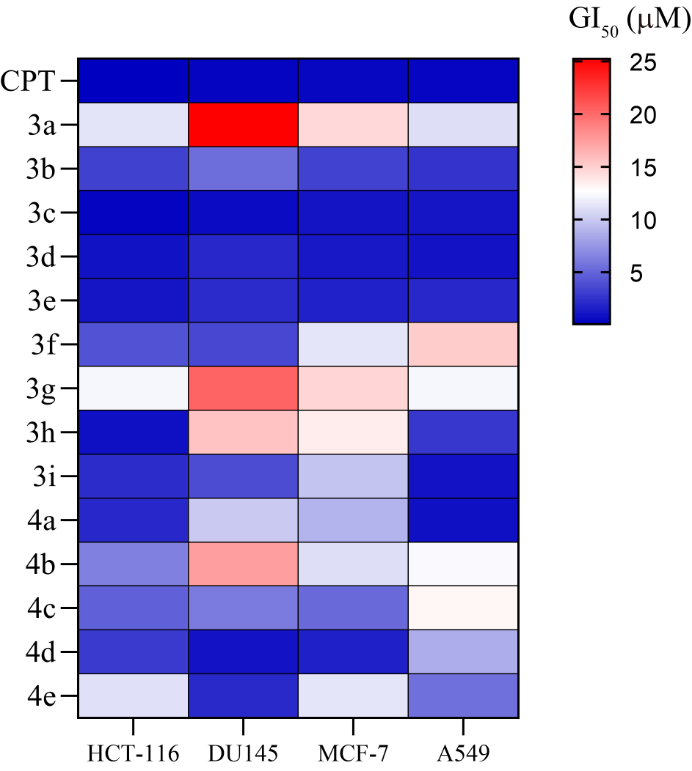


**Figure S3.** The heatmap of MTT results of all synthesized compounds.


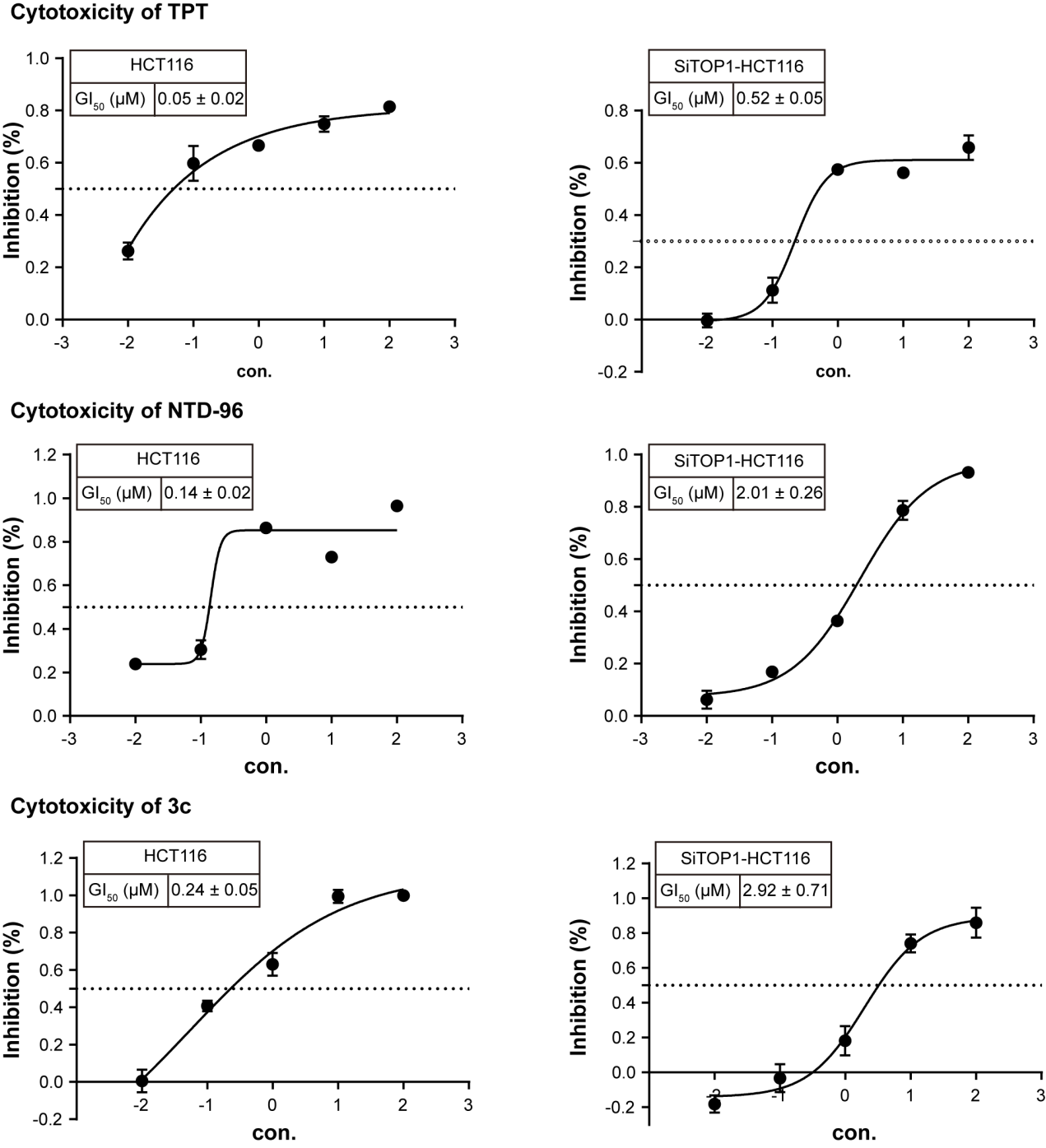


**Figure S4.** The cell proliferation inhibition curves of TOP1 inhibitors against HCT116 and SiTOP1-HCT116 cells.


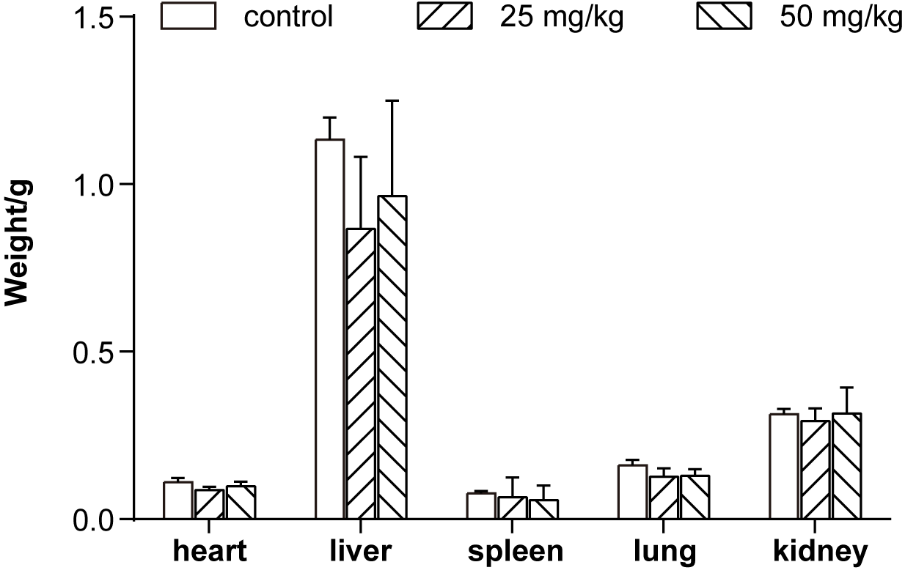


**Figure S5.** The effect to organs after treated with **3c** *in vivo*.

Characterization Data of the synthesized compounds.

**3,4-dimethoxybenzamide (2a)**: White solid, yield 99% (Br) and 99% (I). ^1^H NMR (400 MHz, CDCl_3_) δ 7.46 (d, *J* = 2.0 Hz, 1H), 7.32 (dd, *J* = 8.4, 2.0 Hz, 1H), 6.88 (d, *J* = 8.4 Hz, 1H), 5.74 (s, 2H), 3.94 (s, 3H), 3.94 (s, 3H).

**Benzamide (2b)**: White solid, yield 98% (Br) and 99% (I). ^1^H NMR (400 MHz, CDCl_3_) δ 8.10 (d, 1H), 7.70 – 7.64 (m, 1H), 7.57 – 7.52 (m, 1H), 7.50 – 7.43 (m, 2H).

**2-naphthamide (2c)**: White solid, yield 94% (Br) and 97% (I). ^1^H NMR (400 MHz, CDCl_3_) δ 7.96 – 7.93 (m, 2H), 7.88 – 7.87 (m, 2H), 7.82 – 7.78 (m, 2H), 7.24 (s, 1H), 6.29 (m, 2H).

**quinoline-7-carboxamide (2d**): White solid, yield 90% (Br) and 96% (I). ^1^H NMR (400 MHz, CDCl_3_) δ 9.59 (d, *J* = 8.3 Hz, 1H), 8.47 (d, *J* = 5.5 Hz, 1H), 7.87 – 7.79 (m, 2H), 7.76 – 7.65 (m, 2H), 4.35 – 4.28 (m, 2H).

**3-methoxybenzamide (2e)**: White solid, yield 94% (Br) and 98% (I). ^1^H NMR (400 MHz, DMSO) δ 8.44 (s, 1H), 7.44 – 7.32 (m, 3H), 7.07 (dd, *J* = 8.0, 2.4 Hz, 1H), 3.79 (s, 3H), 3.31 – 3.24 (m, 2H), 1.11 (t, *J* = 7.2 Hz, 3H).

**N-ethyl-3,4-dimethoxybenzamide (2f)**: White solid, yield 93% (Br) and 97% (I). ^1^H NMR (400 MHz, CDCl_3_) δ 7.42 (d, *J* = 2.0 Hz, 1H), 7.25 (dd, *J* = 8.4, 2.0 Hz, 1H), 6.85 (d, *J* = 8.4 Hz, 1H), 6.04 (s, 1H), 3.93 (s, 3H), 3.92 (s, 3H), 3.55 – 3.45 (m, 2H), 1.25 (t, *J* = 7.2 Hz, 3H).

**3-nitrobenzamide (2g)**: Light yellow solid, yield 92% (Br) and 95% (I). ^1^H NMR (400 MHz, CDCl_3_) δ 8.58 (s, 1H), 8.34 (d, *J* = 8.0 Hz, 1H), 8.16 (d, *J* = 8.0 Hz, 1H), 7.72 – 7.61 (m, 1H), 6.36 (s, 1H), 3.59 – 3.45 (m, 2H), 1.29 (t, *J* = 7.2 Hz, 3H).

**3-chlorobenzamide (2h):** White solid, yield 94% (Br) and 96% (I). ^1^H NMR (400 MHz, DMSO) δ 7.74 (s, 1H), 7.63 (d, *J* = 8.0 Hz, 1H), 7.45 (d, *J* = 8.0 Hz, 1H), 7.39 – 7.32 (m, 1H), 6.17 (s, 1H), 3.54 – 3.44 (m, 2H), 1.25 (t, *J* = 7.2 Hz, 3H).

**3,4-dimethoxy-N-(prop-2-yn-1-yl)benzamide (2i)**: Yellow solid, yield 92% (Br) and 95% (I). ^1^H NMR (400 MHz, CDCl_3_) δ 7.42 (d, *J* = 2.0 Hz, 1H), 7.30 (dd, *J* = 8.4, 2.0 Hz, 1H), 6.86 (d, *J* = 8.4 Hz, 1H), 6.32 (s, 1H), 4.26 – 4.20 (m, 2H), 3.92 (s, 3H), 3.91 (s, 3H), 2.27 (t, *J* = 2.8 Hz, 1H).

**3,4-dimethoxy-N-(4-methoxybenzyl)benzamide (2j)**: White solid, yield 90% (Br) and 95% (I). ^1^H NMR (400 MHz, CDCl_3_) δ 7.45 (d, *J* = 2.0 Hz, 1H), 7.30 (s, 1H), 7.29 – 7.26 (m, 2H), 6.89 (d, *J* = 8.4 Hz, 2H), 6.84 (d, *J* = 8.4 Hz, 1H), 6.26 (s, 1H), 4.57 (d, *J* = 5.6 Hz, 2H), 3.93 (s, 3H), 3.91 (s, 3H), 3.81 (s, 3H).

**3,4-dimethoxy-N-(3-methoxyphenyl)benzamide (2k)**: White solid, yield 93% (Br) and 95% (I). ^1^H NMR (400 MHz, CDCl_3_) δ 7.72 (s, 1H), 7.54 (s, 1H), 7.52 (s, 1H), 7.49 (d, *J* = 2.0 Hz, 1H), 7.38 (dd, *J* = 8.4, 2.0 Hz, 1H), 6.93 – 6.88 (m, 3H), 3.94 (s, 3H), 3.94 (s, 3H), 3.81 (s, 3H).

**N-(2-(dimethylamino)ethyl)-3,4-dimethoxybenzamide (2l)**: White solid, yield 93% (Br) and 94% (I). ^1^H NMR (400 MHz, CDCl_3_) δ 7.46 (s, 1H), 7.31 (d, *J* = 8.4 Hz, 1H), 6.86 (d, *J* = 8.4 Hz, 2H), 3.93 (s, 3H), 3.91 (s, 3H), 3.55 – 3.48 (m, 2H), 2.54 (t, *J* = 6.0 Hz, 2H), 2.29 (s, 6H).

**N-(naphthalen-1-yl)benzo[d][1,3]dioxole-5-carboxamide (2m)**: White solid, yield 94% (Br) and 95% (I). ^1^H NMR (500 MHz, CDCl_3_) δ 8.08 (s, 1H), 8.01 (d, *J* = 7.5 Hz, 1H), 7.90 (d, *J* = 7.0 Hz, 2H), 7.75 (d, *J* = 8.5 Hz, 1H), 7.57 – 7.50 (m, 4H), 7.48 (s, 1H), 6.94 (d, *J* = 8.0 Hz, 1H), 6.09 (s, 2H). ^13^C NMR (100 MHz, CDCl_3_) δ 165.7, 150.9, 148.4, 134.3, 132.5, 129.1, 128.9, 127.6, 126.5, 126.2, 125.9, 121.9, 121.4, 120.9, 108.3, 108.1, 102.0.

**3-methyl-N-(naphthalen-1-yl)benzamide (2n)**: White solid, yield 92% (Br) and 95% (I). ^1^H NMR (500 MHz, CDCl_3_) δ 8.20 (s, 1H), 8.05 (d, *J* = 7.0 Hz, 1H), 7.93 – 7.88 (m, 2H), 7.83 – 7.80 (m, 1H), 7.79 – 7.73 (m, 2H), 7.56 – 7.51 (m, 3H), 7.45 – 7.39 (m, 2H), 2.47 (s, 3H). ^13^C NMR (101 MHz, CDCl_3_) δ 165.7, 150.9, 148.3, 134.2, 132.5, 129.0, 128.9, 127.6, 126.5, 126.1, 125.9, 121.9, 121.3, 120.8, 108.3, 108.0, 102.0.

**3-chloro-N-(naphthalen-1-yl)benzamide (2o)**: White solid, yield 90% (Br) and 94% (I). ^1^H NMR (400 MHz, DMSO) δ 10.57 (s, 1H), 8.14 (s, 1H), 8.05 (d, *J* = 7.6 Hz, 1H), 8.02 – 7.96 (m, 2H), 7.88 (d, *J* = 8.0 Hz, 1H), 7.73 – 7.69 (m, 1H), 7.64 – 7.53 (m, 5H). ^13^C NMR (100 MHz, DMSO) δ 164.8, 136.4, 133.7, 133.5, 133.3, 131.5, 130.5, 129.1, 128.1, 127.6, 126.6, 126.5, 126.1, 126.0, 125.5, 123.9, 123.3.

**3-hydroxy-N-(naphthalen-1-yl)benzamide (2p)**: White solid, yield 92% (Br) and 94% (I). ^1^H NMR (400 MHz, DMSO) δ 10.35 (s, 1H), 9.76 (s, 1H), 8.00 – 7.94 (m, 2H), 7.86 (d, *J* = 8.0 Hz, 1H), 7.59 – 7.52 (m, 5H), 7.46 – 7.43 (m, 1H), 7.35 (t, *J* = 8.0 Hz, 1H), 7.03 – 6.98 (m, 1H). ^13^C NMR (100 MHz, DMSO) δ 166.2, 157.4, 135.9, 133.9, 133.8, 129.5, 129.2, 128.0, 126.2, 126.0, 125.9, 125.5, 123.9, 123.3, 118.6, 118.3, 114.7.

**3-methoxy-N-(naphthalen-1-yl)benzamide (2q)**: White solid, yield 91% (Br) and 95% (I). ^1^H NMR (400 MHz, CDCl_3_) δ 8.39 – 8.33 (m, 1H), 7.95 (d, *J* = 8.0 Hz, 1H), 7.92 – 7.86 (m, 1H), 7.75 – 7.63 (m, 2H), 7.62 – 7.53 (m, 4H), 7.48 (t, *J* = 7.2 Hz, 1H), 6.96 – 6.90 (m, 2H), 3.83 (s, 3H). ^13^C NMR (100 MHz, CDCl_3_) δ 167.5, 156.8, 134.6, 133.8, 131.2, 131.0, 130.2, 128.5, 127.4, 126.7, 125.4, 125.1, 124.8, 121.9, 114.4, 55.6.

**N,5-diethyl-3,4,8,9-tetramethoxy-6-oxo-5,6-dihydrophenanthridine-1-carboxamide** **(3a)**: White solid, yield 90%, mp = 141.3–144.2 ^o^C. ^1^H NMR (400 MHz, CDCl_3_) δ 8.75 (s, 1H), 7.88 (s, 1H), 7.23 (s, 1H), 5.87 (t, *J* = 5.2 Hz, 1H), 4.42 – 4.36 (m, 2H), 4.02 (s, 3H), 4.01 (s, 3H), 3.97 (s, 3H), 3.93 (s, 3H), 3.55 – 3.47 (m, 2H), 1.25 (t, *J* = 7.2 Hz, 3H), 1.15 (t, *J* = 7.2 Hz, 3H). ^13^C NMR (126 MHz, CDCl_3_) δ 169.29, 162.36, 152.59, 149.29, 148.35, 147.73, 129.65, 127.15, 123.02, 120.71, 117.10, 113.66, 108.80, 108.29, 60.49, 56.55, 55.99, 43.67, 35.40, 14.60, 13.81. HRMS (ESI) *m/z*: 415.1885 [M + H]^+^, calcd for C_22_H_27_N_2_O_6_ 415.1864.

**N,5-diethyl-6-oxo-5,6-dihydrophenanthridine-1-carboxamide (3b)**: White solid, yield 78%, mp = 153.7–155.7 ^o^C. ^1^H NMR (400 MHz, CDCl_3_) δ 8.52 – 8.47 (m, 1H), 8.35 – 8.30 (m, 1H), 8.23 (d, *J* = 8.4 Hz, 1H), 7.79 – 7.73 (m, 1H), 7.62 – 7.57 (m, 1H), 7.56 – 7.51 (m, 1H), 7.30 (t, *J* = 8.0 Hz, 1H), 5.86 (s, 1H), 4.52 – 4.45 (m, 2H), 3.58 – 3.49 (m, 2H), 1.29 – 1.24 (m, 6H). ^13^C NMR (101 MHz, CDCl_3_) δ 170.04, 162.80, 134.77, 133.38, 132.79, 130.58, 128.87, 128.54, 127.34, 125.84, 125.02, 122.35, 121.88, 121.82, 42.00, 35.41, 14.68, 14.06. HRMS (ESI) *m/z*: 295.1444 [M + H]^+^, calcd for C_18_H_19_N_2_O_2_ 295.1441.

**N,5-diethyl-4,9-dimethoxy-6-oxo-5,6-dihydrophenanthridine-1-carboxamide (3c)**: White solid, yield 69%, mp = 151.7–154.2 ^o^C. ^1^H NMR (400 MHz, CDCl_3_) δ 8.65 (d, *J* = 2.5 Hz, 1H), 8.45 (d, *J* = 8.8 Hz, 1H), 7.60 (d, *J* = 8.6 Hz, 1H), 7.12 (dd, *J* = 8.8, 2.5 Hz, 1H), 6.86 (d, *J* = 8.6 Hz, 1H), 5.84 – 5.75 (m, 1H), 4.43 – 4.36 (m, 2H), 4.08 (s, 3H), 3.96 (s, 3H), 3.54 – 3.46 (m, 2H), 1.26 – 1.22 (m, 3H), 1.14 (t, *J* = 6.9 Hz, 3H). ^13^C NMR (126 MHz, CDCl_3_) δ 169.40, 163.12, 162.84, 159.92, 134.74, 133.31, 130.85, 130.45, 128.76, 120.58, 119.79, 114.88, 111.62, 105.38, 77.41, 77.16, 76.91, 56.26, 55.54, 43.96, 35.31, 14.73, 13.90. HRMS (ESI) *m/z*: 355.1675 [M + H]^+^, calcd for C_20_H_23_N_2_O_4_ 355.1665.

**N,5-diethyl-4,9-dinitro-6-oxo-5,6-dihydrophenanthridine-1-carboxamide (3d)**: White solid, yield 58%, mp = 163.3–164.2 ^o^C. ^1^H NMR (400 MHz, CDCl_3_) δ 9.24 (d, *J* = 1.8 Hz, 1H), 8.35 (d, *J* = 8.5 Hz, 1H), 7.54 (dd, *J* = 8.5, 1.8 Hz, 1H), 7.47 (d, *J* = 8.1 Hz, 1H), 7.36 (d, *J* = 8.1 Hz, 1H), 6.03 (s, 1H), 4.37 (q, *J* = 6.8 Hz, 2H), 3.56 – 3.46 (m, 2H), 1.25 (t, *J* = 7.2 Hz, 3H), 1.17 (t, *J* = 6.8 Hz, 3H). ^13^C NMR (101 MHz, CDCl_3_) δ 168.38, 161.89, 138.66, 138.17, 135.64, 133.57, 130.15, 129.92, 129.28, 128.52, 126.53, 126.37, 125.16, 119.47, 43.68, 35.45, 14.66, 13.75. HRMS (ESI) *m/z*: 385.1165 [M + H]^+^, calcd for C_18_H_17_N_4_O_6_ 385.1156.

**N,5-diethyl-6-oxo-4,9-bis(trifluoromethyl)-5,6-dihydrophenanthridine-1-carboxamide (3e)**: White solid, yield 67%, mp = 181.7–182.3 ^o^C. ^1^H NMR (500 MHz, CDCl_3_) δ 8.68 (s, 1H), 8.52 (s, 1H), 8.33 (d, *J* = 8.5 Hz, 1H), 7.97 (d, *J* = 8.5 Hz, 1H), 7.80 (s, 1H), 6.22 (s, 1H), 4.48 (q, *J* = 6.5 Hz, 2H), 3.62 – 3.53 (m, 2H), 1.37 – 1.27 (m, 6H). ^13^C NMR (126 MHz, CDCl_3_) δ 168.37, 161.62, 137.80, 135.19, 131.27, 129.71, 129.41, 129.38, 128.21, 128.18, 128.05, 126.57, 126.54, 125.93, 124.99, 124.71, 123.11, 122.65, 122.62, 120.77, 42.28, 35.66, 14.60, 13.94. HRMS (ESI) *m/z*: 431.1356 [M + H]^+^, calcd for C_20_H_17_N_2_O_2_F_6_ 431.1345.

**N,5-diethyl-3,4,8,9-tetrafluoro-6-oxo-5,6-dihydrophenanthridine-1-carboxamide (3f)**: White solid, yield 75%, mp = 133.5–134.1 ^o^C. ^1^H NMR (400 MHz, CDCl_3_) δ 8.48 – 8.40 (m, 1H), 8.32 (dd, *J* = 10.4, 8.5 Hz, 1H), 7.46 (t, *J* = 9.0 Hz, 1H), 5.88 – 5.79 (m, 1H), 4.44 – 4.36 (m, 2H), 3.56 – 3.48 (m, 2H), 1.28 (t, *J* = 7.3 Hz, 3H), 1.22 (t, *J* = 7.0 Hz, 3H). ^13^C NMR (101 MHz, CDCl_3_) δ 167.43, 160.84, 147.15, 132.19, 127.74, 124.00, 123.32, 118.89, 118.69, 117.39, 116.01, 115.73, 115.52, 100.13, 43.32, 35.60, 14.58, 13.81. HRMS (ESI) *m/z*: 367.1053 [M + H]^+^, calcd for C_18_H_15_N_2_O_2_F_4_ 367.1064.

**3,4,8,9-tetramethoxy-N,5-bis(3-methoxyphenyl)-6-oxo-5,6-dihydrophenanthridine-1-carboxamide (3g)**: White solid, yield 65%, mp = 171.3–173.2 ^o^C. ^1^H NMR (400 MHz, CDCl_3_) δ 8.98 (s, 1H), 7.92 (s, 1H), 7.62 – 7.57 (m, 2H), 7.55 – 7.51 (m, 2H), 7.47 – 7.43 (m, 2H), 7.36 – 7.31 (m, 2H), 6.66 (s, 1H), 6.16 (s, 1H), 4.12 (s, 3H), 4.02 (s, 3H), 3.99 (s, 3H), 3.88 (s, 3H), 3.76 (s, 3H), 3.61 (s, 3H). ^13^C NMR (101 MHz, CDCl_3_) δ 168.08, 161.35, 153.82, 150.15, 149.42, 148.52, 145.52, 138.78, 138.27, 133.88, 132.23, 130.23, 129.22, 129.04, 128.90, 128.69, 124.47, 120.01, 119.03, 113.19, 111.80, 110.53, 109.42, 105.25, 102.44, 100.74, 56.90, 56.35, 56.24, 55.83. HRMS (ESI) *m/z*: 571.2019 [M + H]^+^, calcd for C_32_H_31_N_2_O_8_ 571.2021.

**3,4,8,9-tetramethoxy-N,5-bis(4-methoxybenzyl)-6-oxo-5,6-dihydrophenanthridine-1-carboxamide (3h)**: White solid, yield 55%, mp = 183.4–183.9 ^o^C. ^1^H NMR (400 MHz, CDCl_3_) δ 8.76 (s, 1H), 7.86 (s, 1H), 7.22 – 7.17 (m, 3H), 6.87 – 6.82 (m, 4H), 6.67 (d, *J* = 8.8 Hz, 2H), 5.94 (t, 1H), 5.43 (s, 2H), 4.32 (d, *J* = 5.2 Hz, 2H), 4.02 (s, 3H), 3.97 (s, 3H), 3.93 (s, 3H), 3.91 (s, 3H), 3.79 (s, 3H), 3.69 (s, 3H). ^13^C NMR (126 MHz, CDCl_3_) δ 168.92, 159.39, 158.56, 152.91, 149.58, 148.40, 148.04, 130.45, 129.87, 129.78, 129.59, 127.77, 127.26, 122.74, 117.10, 114.33, 113.88, 113.85, 109.31, 108.54, 60.53, 56.56, 56.17, 56.08, 55.45, 55.30, 51.15, 44.00. HRMS (ESI) *m/z*: 599.2368 [M + H]^+^, calcd for C_34_H_35_N_2_O_8_ 599.2388.

**3,4,8,9-tetramethoxy-N-(4-methoxybenzyl)-6-oxo-5,6-dihydrophenanthridine-1-carboxamide (3i)**: White solid, yield 25%, mp = 175.9–176.8 ^o^C. ^1^H NMR (400 MHz, CDCl_3_) δ 7.16 (s, 1H), 7.06 (s, 1H), 7.06 – 7.03 (m, 1H), 6.89 (d, *J* = 8.8 Hz, 2H), 6.74 (d, *J* = 8.8 Hz, 2H), 6.59 (s, 1H), 6.53 (s, 1H), 5.39 (s, 1H), 4.44 – 4.36 (m, 1H), 4.25 – 4.18 (m, 1H), 3.94 (s, 3H), 3.92 (s, 3H), 3.82 (s, 3H), 3.79 (s, 3H), 3.74 (s, 3H). ^13^C NMR (101 MHz, CDCl_3_) δ 171.29, 169.18, 158.96, 150.11, 149.84, 148.49, 148.32, 132.56, 132.13, 130.23, 129.11, 128.35, 126.91, 113.90, 113.07, 112.83, 110.62, 110.52, 56.27, 56.21, 56.15, 56.10, 55.36, 43.33. HRMS (ESI) *m/z*: 478.1795 [M + H]^+^, calcd for C_26_H_27_N_2_O_7_ 478.1789.

**N-(2-chloroacetyl)-N,5-diethyl-3,4,8,9-tetramethoxy-6-oxo-5,6-dihydrophenanthridine-1-carboxamide (4a)**: White solid, yield 65%, mp = 141.3–144.2 ^o^C. ^1^H NMR (400 MHz, CDCl_3_) δ 8.81 (s, 1H), 7.96 (s, 1H), 6.98 (s, 1H), 4.72 (s, 2H), 4.05 (s, 6H), 3.96 (s, 3H), 3.95 (s, 3H), 3.87 – 3.41 (m, 2H), 1.92 – 1.75 (m, 2H), 1.26 (t, *J* = 6.8 Hz, 4H), 1.09 (t, *J* = 7.0 Hz, 3H). ^13^C NMR (101 MHz, CDCl_3_) δ 172.41, 169.77, 161.78, 153.01, 150.03, 148.94, 148.02, 130.05, 126.94, 120.86, 119.77, 117.15, 112.45, 109.08, 108.71, 60.70, 56.74, 56.28, 56.16, 46.71, 43.20, 42.50, 13.81, 13.28. HRMS (ESI) *m/z*: 429.1521 [M + H]^+^, calcd for C_24_H_28_N_2_O_7_Cl 491.1529.

**N-(cyclopropanecarbonyl)-N,5-diethyl-3,4,8,9-tetramethoxy-6-oxo-5,6-dihydrophenanthridine-1-carboxamide (4b)**: White solid, yield 55%, mp = 133.1–134.6 ^o^C. ^1^H NMR (400 MHz, CDCl_3_) δ 8.78 (s, 1H), 7.95 (s, 1H), 6.98 (s, 1H), 4.33 – 4.16 (m, 2H), 4.04 (s, 3H), 4.04 (s, 3H), 3.94 (s, 3H), 3.92 (s, 3H), 3.92 – 3.89 (m, 2H), 1.90 – 1.83 (m, 1H), 1.28 – 1.23 (m, 6H), 1.03 – 0.98 (m, 2H), 0.82 – 0.69 (m, 2H). ^13^C NMR (126 MHz, CDCl_3_) δ 177.37, 170.98, 161.90, 152.77, 149.75, 148.45, 147.94, 130.96, 127.03, 122.20, 121.10, 117.16, 112.66, 108.94, 108.52, 60.61, 56.71, 56.21, 56.09, 44.23, 40.92, 16.27, 14.20, 13.12, 11.24. HRMS (ESI) *m/z*: 483.2122 [M + H]^+^, calcd for C_26_H_31_N_2_O_7_ 483.2126.

**N,5-diethyl-N-(furan-2-carbonyl)-3,4,8,9-tetramethoxy-6-oxo-5,6-dihydrophenanthridine-1-carboxamide (4c)**: White solid, yield 60%, mp = 135.1–137.2 ^o^C. ^1^H NMR (400 MHz, CDCl_3_) δ 8.67 (s, 1H), 7.94 (s, 1H), 7.27 (d, *J* = 3.6 Hz, 1H), 7.11 (s, 1H), 6.83 (d, *J* = 3.6 Hz, 1H), 6.27 (dd, *J* = 3.5, 1.6 Hz, 1H), 4.85 – 4.31 (m, 2H), 4.13 – 4.07 (m, 2H), 4.05 (s, 3H), 4.01 (s, 3H), 3.88 (s, 3H), 3.84 (s, 3H), 1.35 (t, *J* = 6.8 Hz, 3H), 1.20 (t, *J* = 6.8 Hz, 3H). ^13^C NMR (126 MHz, CDCl_3_) δ 170.41, 162.58, 161.82, 152.67, 149.62, 148.43, 147.79, 147.69, 145.99, 131.70, 126.92, 121.46, 121.24, 118.91, 116.96, 113.52, 112.49, 109.06, 108.36, 60.34, 56.65, 56.22, 56.08, 45.00, 41.86, 14.19, 13.17. HRMS (ESI) *m/z*: 509.1940 [M + H]^+^, calcd for C_27_H_29_N_2_O_8_ 509.1918.

**N-(4-chlorobenzoyl)-N,5-diethyl-3,4,8,9-tetramethoxy-6-oxo-5,6-dihydrophenanthridine-1-carboxamide (4d)**: White solid, yield 89%, mp = 167.1–168.2 ^o^C. ^1^H NMR (400 MHz, CDCl_3_) δ 8.50 (s, 1H), 7.97 (s, 1H), 7.14 (s, 1H), 6.93 (d, *J* = 8.8 Hz, 2H), 6.76 (d, *J* = 8.8 Hz, 2H), 4.33 – 4.16 (m, 2H), 4.08 (s, 3H), 4.02 (s, 3H), 3.91 (s, 3H), 3.75 (s, 3H), 3.71 – 3.29 (m, 2H), 1.36 (t, *J* = 7.2 Hz, 3H), 1.04 (t, *J* = 7.2 Hz, 3H). ^13^C NMR (101 MHz, CDCl_3_) δ 171.99, 170.76, 161.83, 152.85, 149.74, 148.96, 148.40, 137.87, 132.95, 132.11, 129.03, 127.76, 126.83, 121.35, 121.21, 114.98, 109.11, 108.30, 60.28, 56.79, 56.30, 56.14, 45.00, 42.23, 14.08, 13.44. HRMS (ESI) *m/z*: 553.1723 [M + H]^+^, calcd for C_29_H_30_N_2_O_7_Cl 553.1736.

**N,5-diethyl-3,4,8,9-tetramethoxy-N-(3-methoxybenzoyl)-6-oxo-5,6-dihydrophenanthridine-1-carboxamide (4e)**: White solid, yield 93%, mp = 132.1–132.9 ^o^C. ^1^H NMR (400 MHz, CDCl_3_) δ 8.55 (s, 1H), 7.99 (s, 1H), 7.08 (s, 1H), 6.88 – 6.83 (m, 1H), 6.67 – 6.61 (m, 2H), 6.49 (s, 1H), 4.29 – 4.11 (m, 2H), 4.07 (s, 3H), 4.01 (s, 3H), 3.88 (s, 3H), 3.72 (s, 3H), 3.67 – 3.47 (m, 2H), 3.32 (s, 3H), 1.36 (t, *J* = 6.9 Hz, 3H), 1.08 (t, *J* = 6.7 Hz, 3H). ^13^C NMR (101 MHz, CDCl_3_) δ 173.19, 171.24, 161.82, 159.05, 152.82, 149.68, 148.38, 148.13, 136.21, 131.90, 128.56, 126.92, 121.97, 121.33, 120.47, 118.88, 117.04, 114.94, 111.82, 109.23, 108.48, 59.67, 56.53, 56.31, 56.14, 54.89, 44.85, 42.21, 14.08, 13.36. HRMS (ESI) *m/z*: 549.2146 [M + H]^+^, calcd for C_30_H_33_N_2_O_8_ 549.2159.


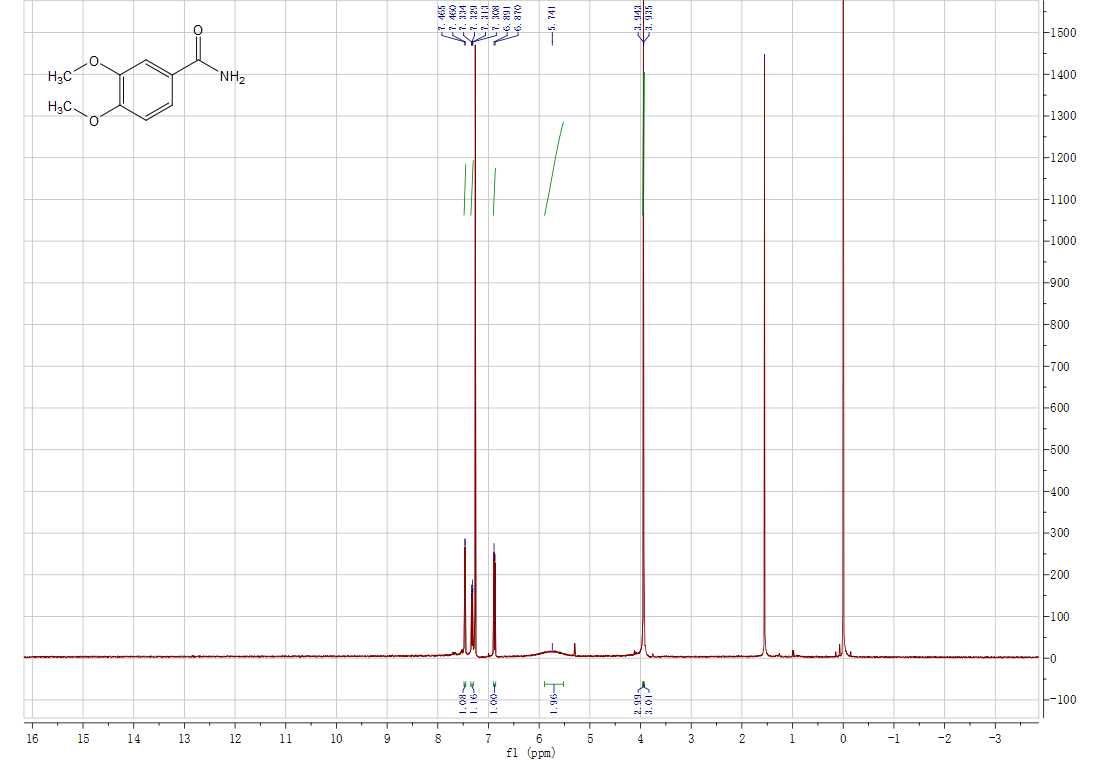


^1^H NMR spectra of compound **2a**

**
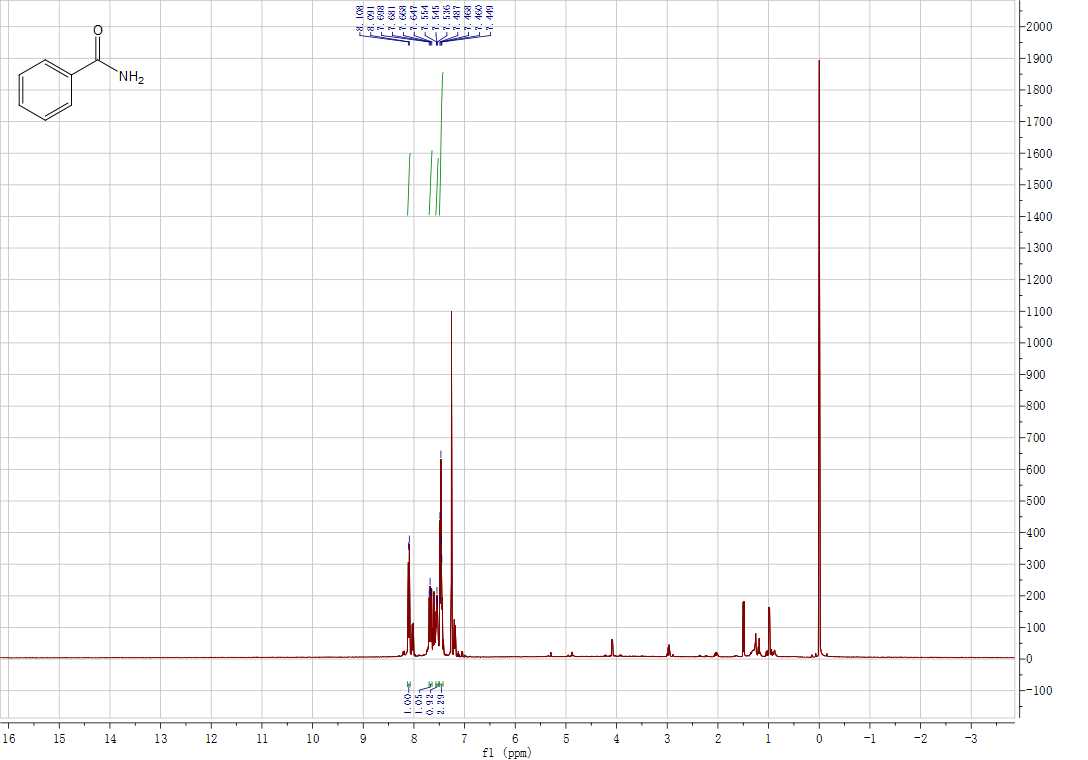
**

^1^H NMR spectra of compound **2b**


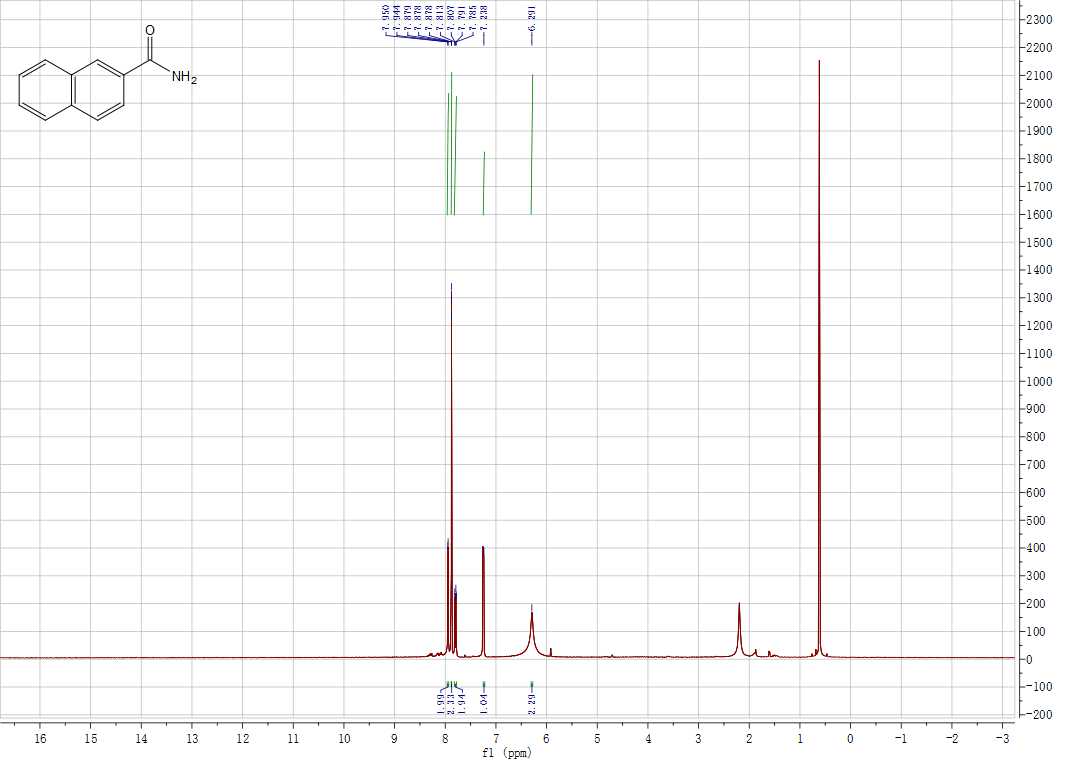


^1^H NMR spectra of compound **2c**

**
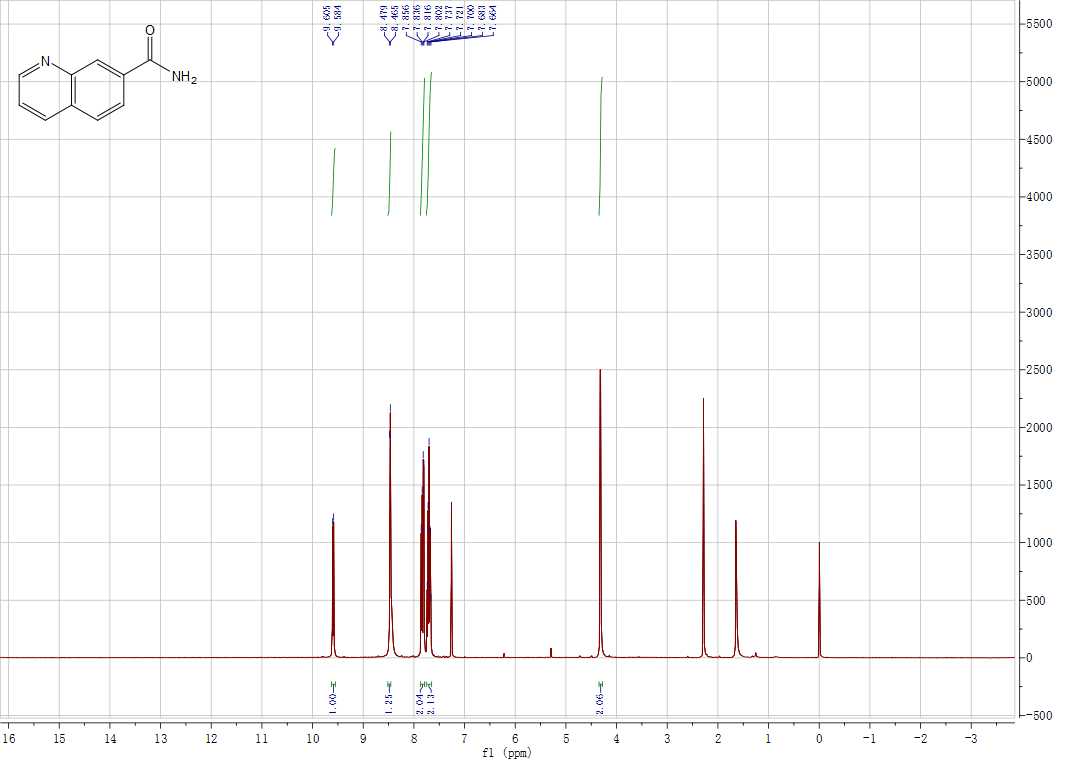
**

^1^H NMR spectra of compound **2d**

**
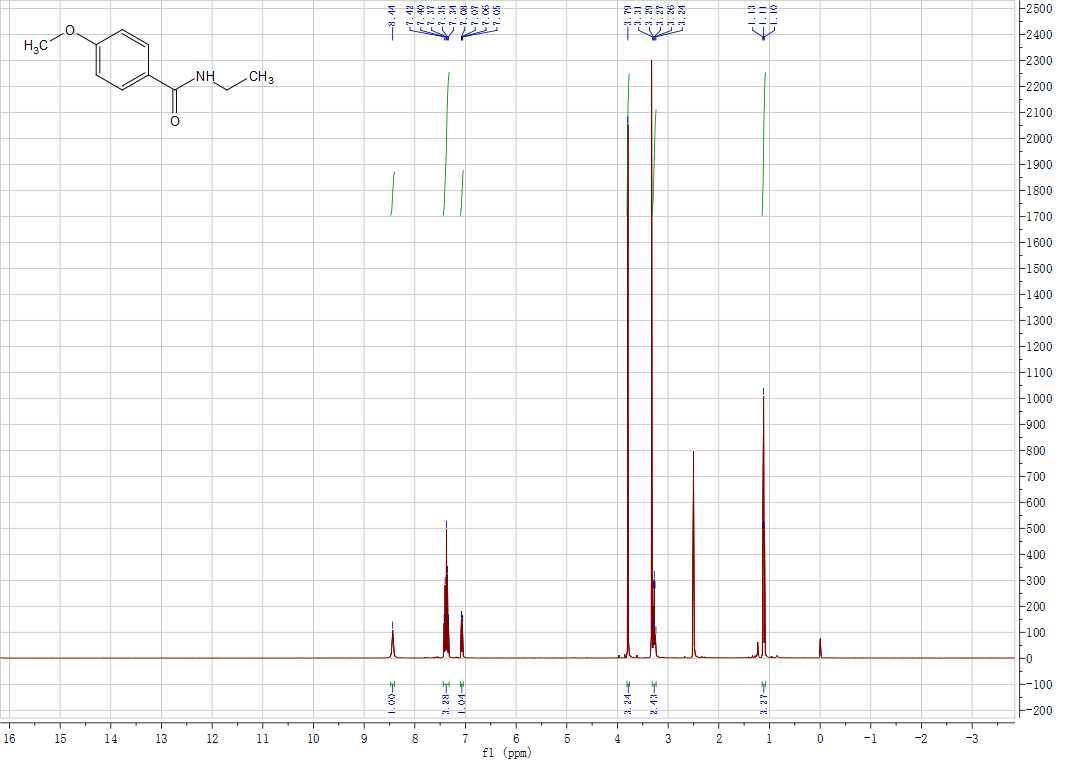
**

^1^H NMR spectra of compound **2e**

**
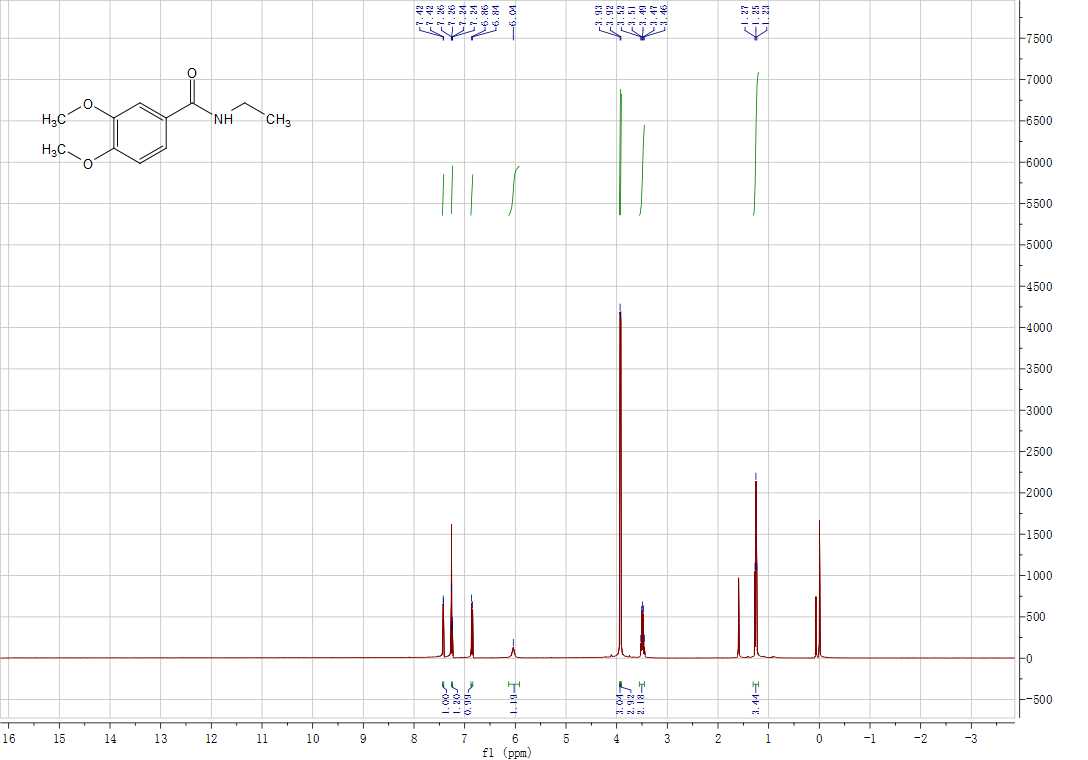
**

^1^H NMR spectra of compound **2f**


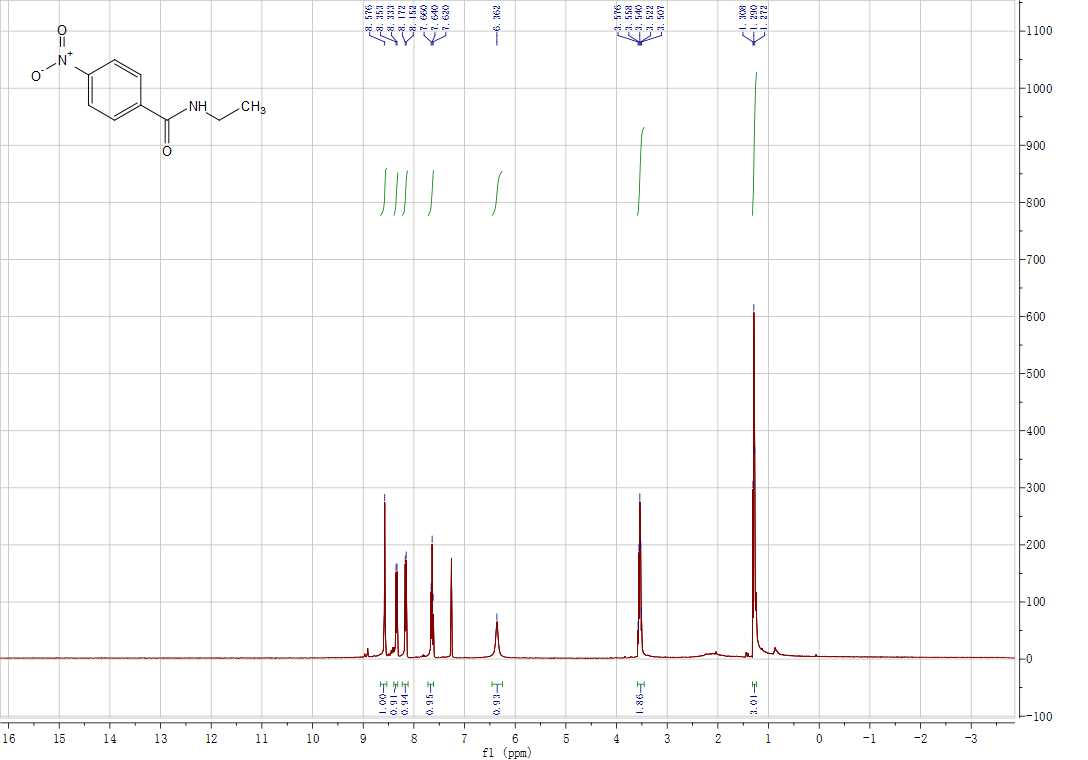


^1^H NMR spectra of compound **2g**


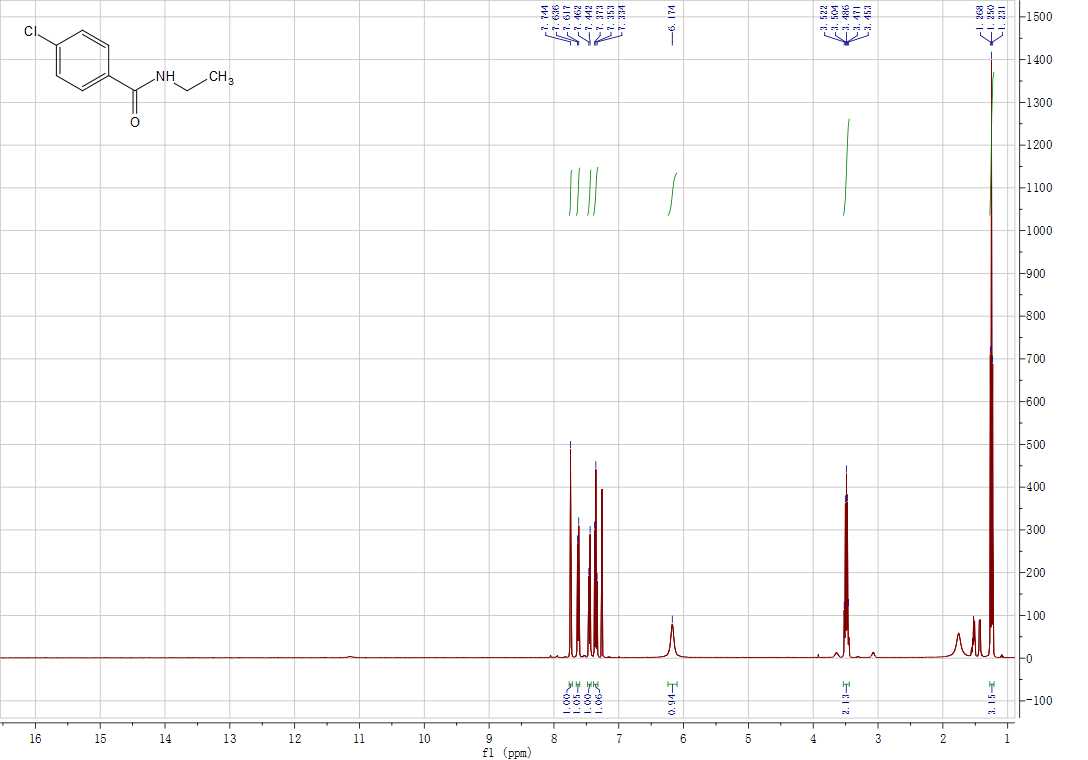


^1^H NMR spectra of compound **2h**


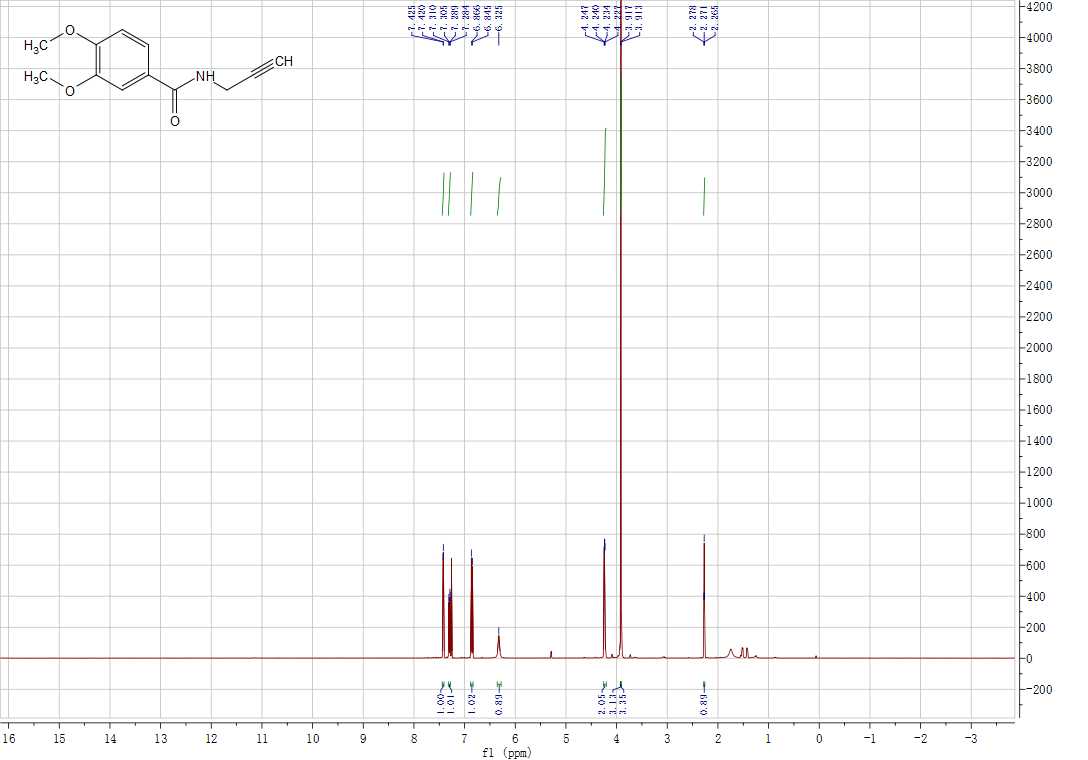


^1^H NMR spectra of compound **2i**


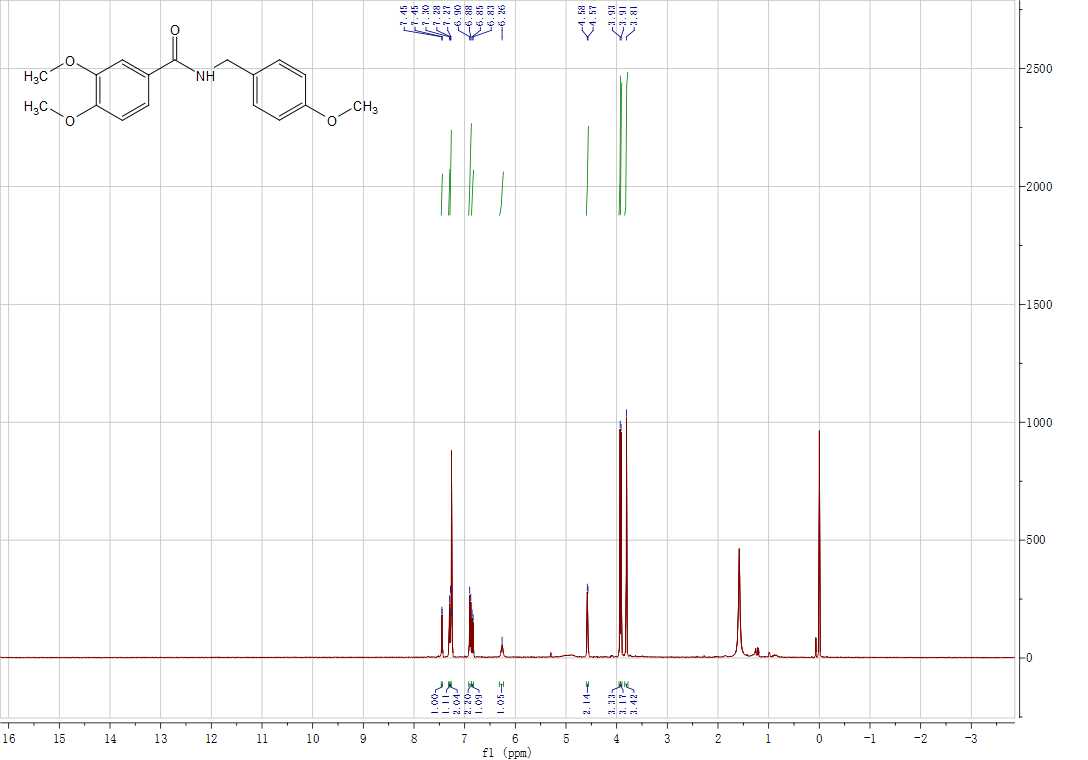


^1^H NMR spectra of compound **2j**


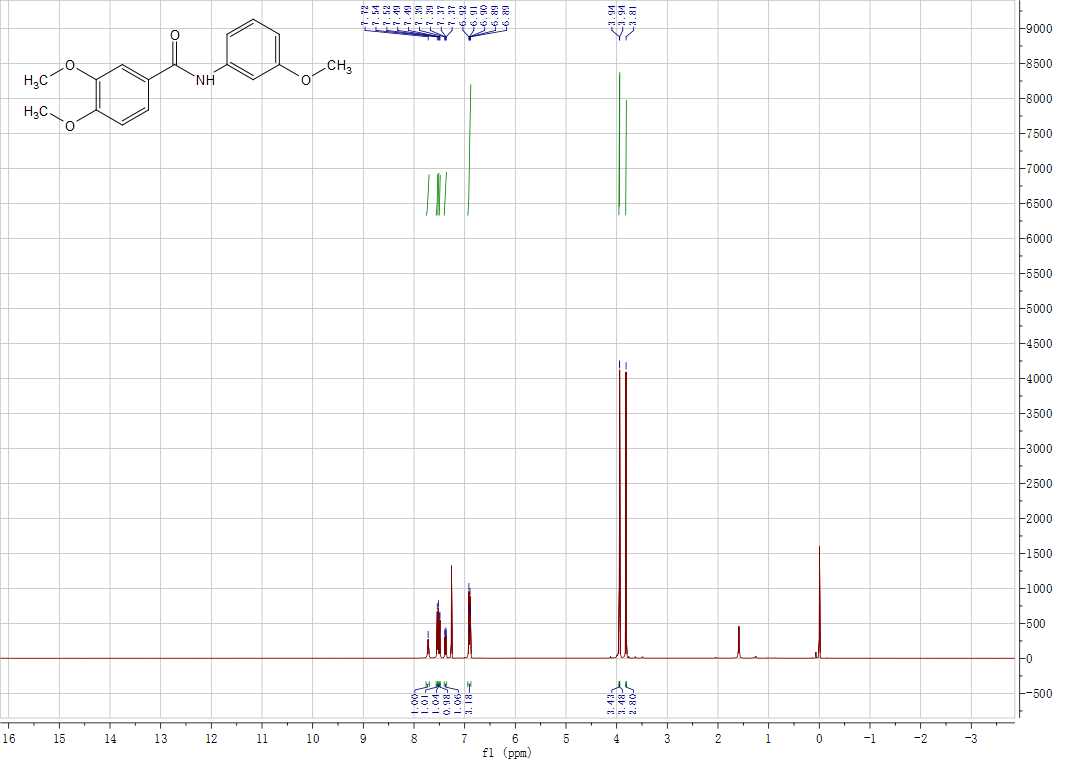


^1^H NMR spectra of compound **2k**


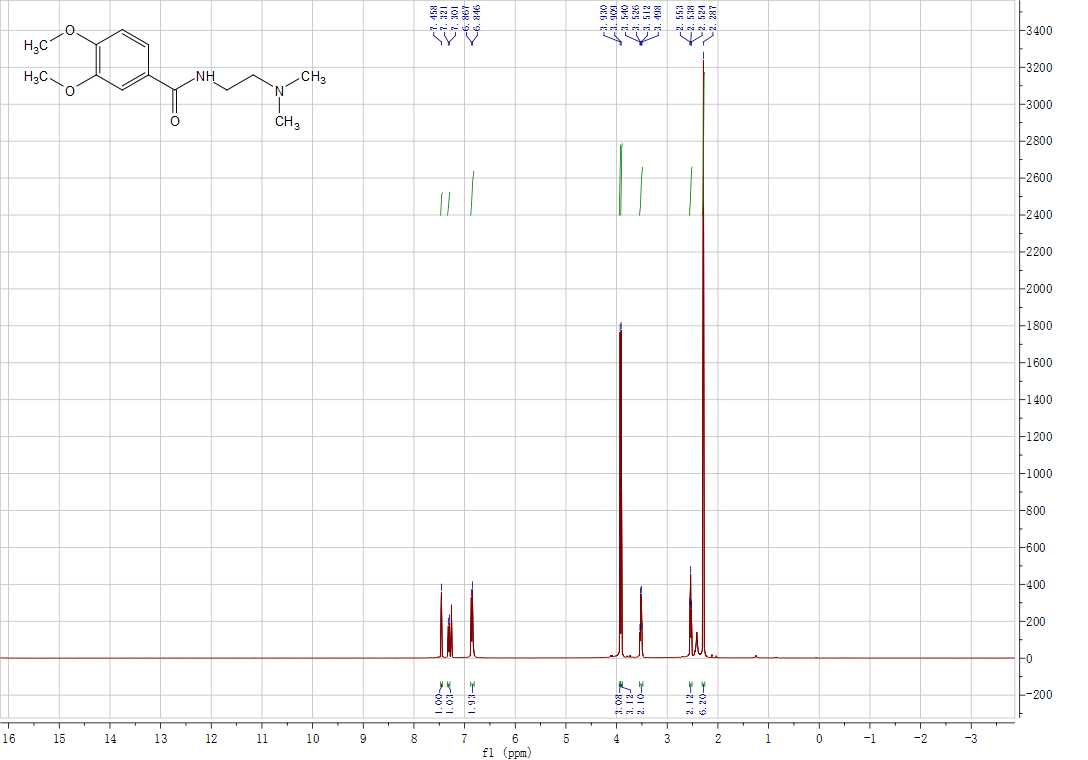


^1^H NMR spectra of compound **2l**


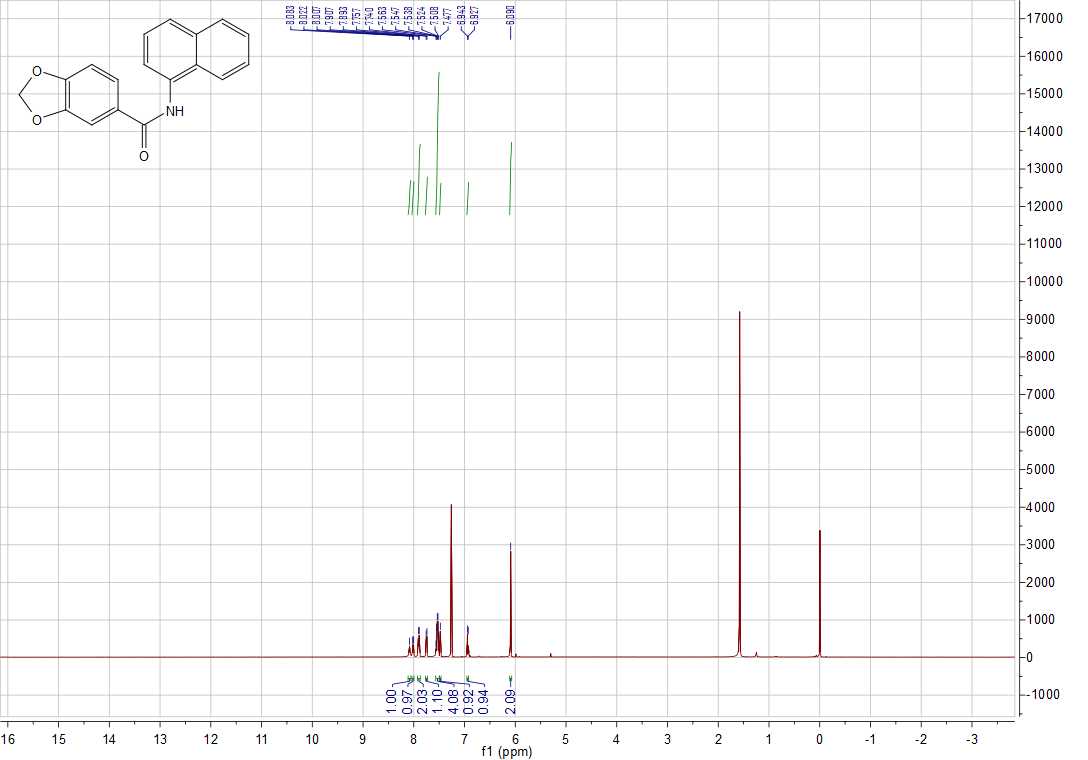


^1^H NMR spectra of compound **2m**


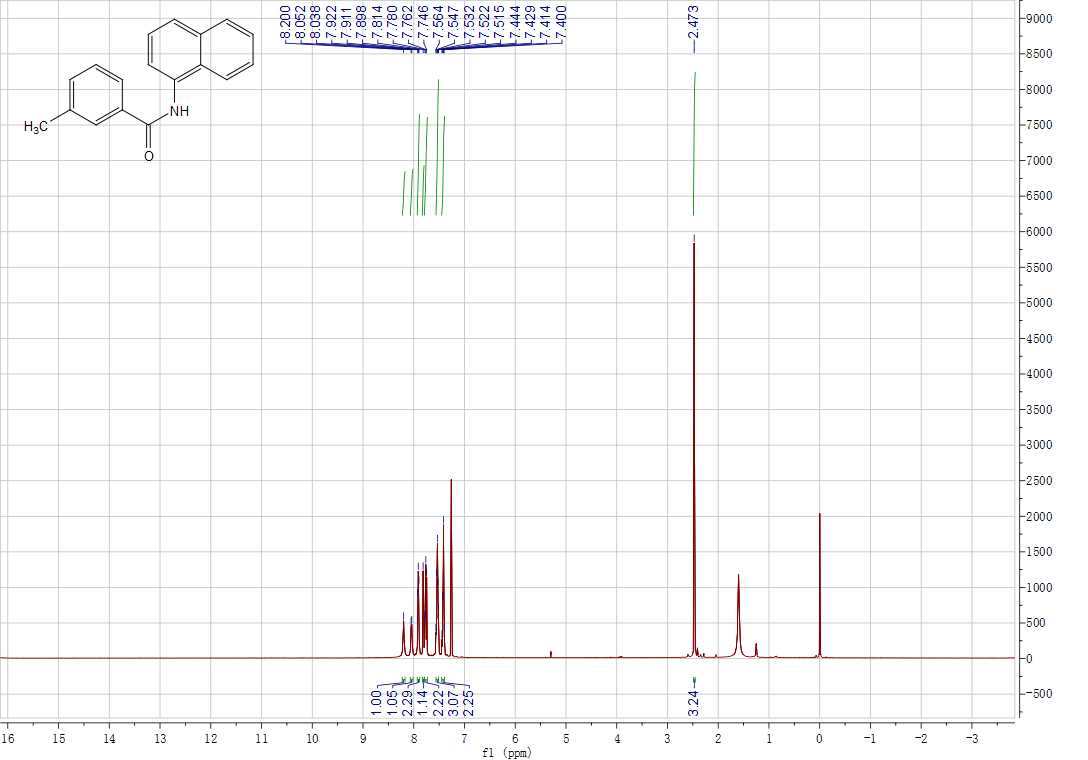


^1^H NMR spectra of compound **2n**


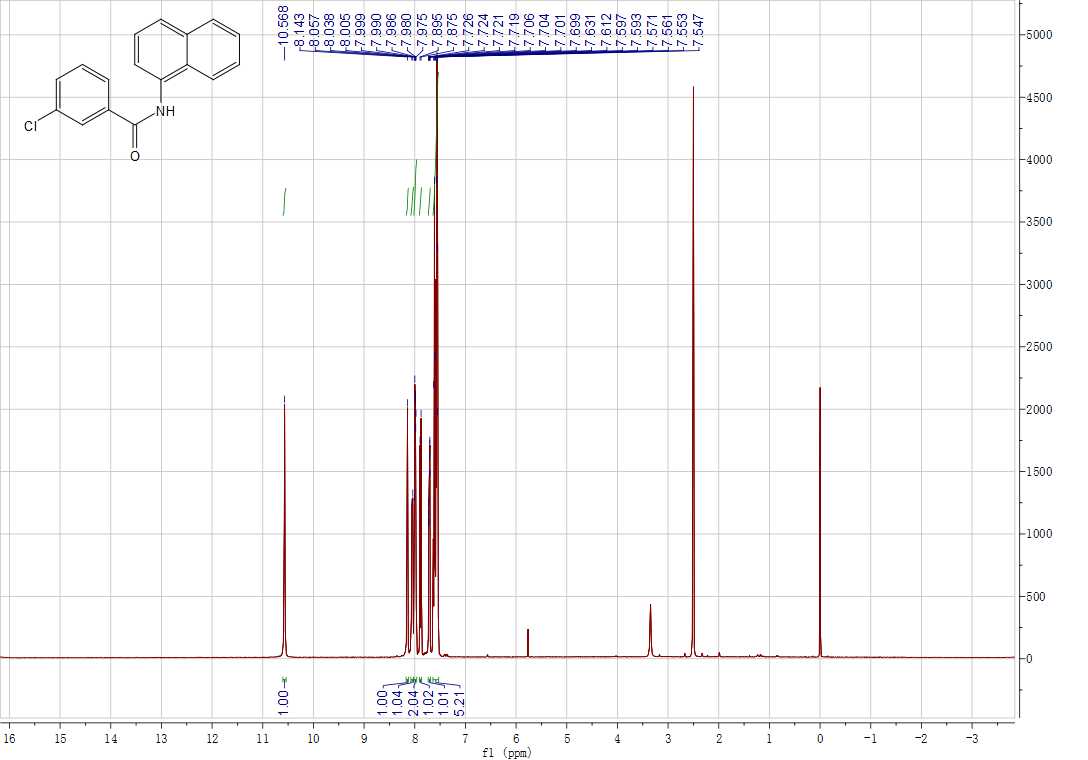


^1^H NMR spectra of compound **2o**


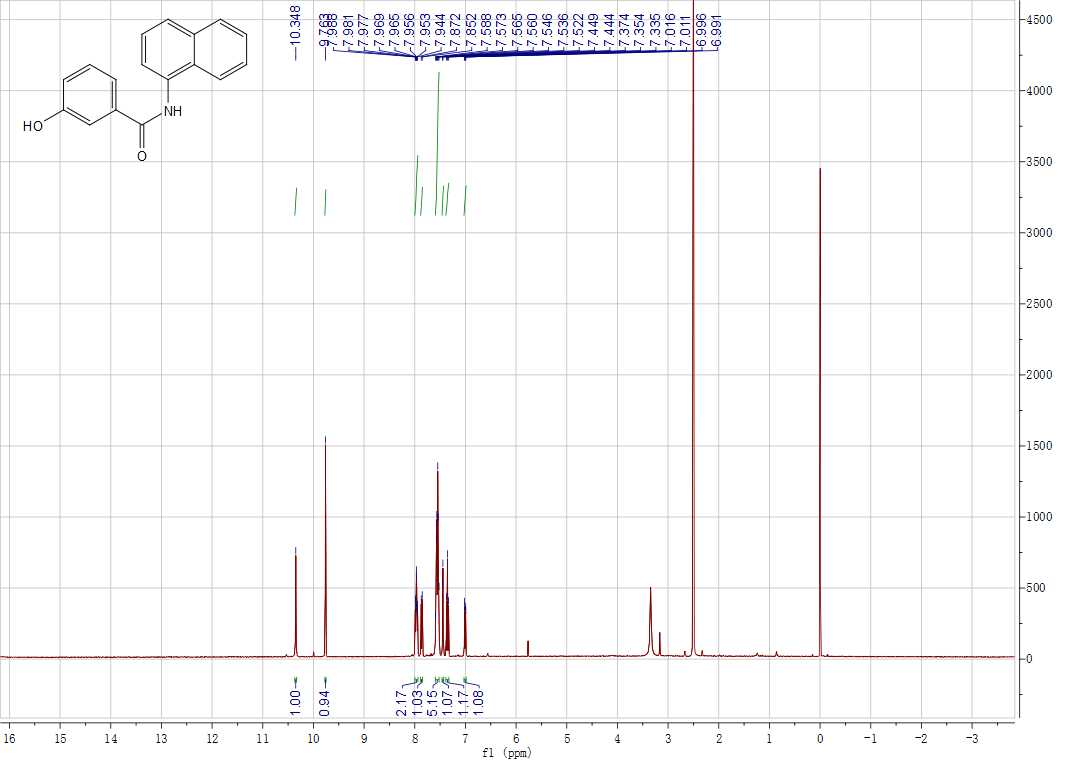


^1^H NMR spectra of compound **2p**


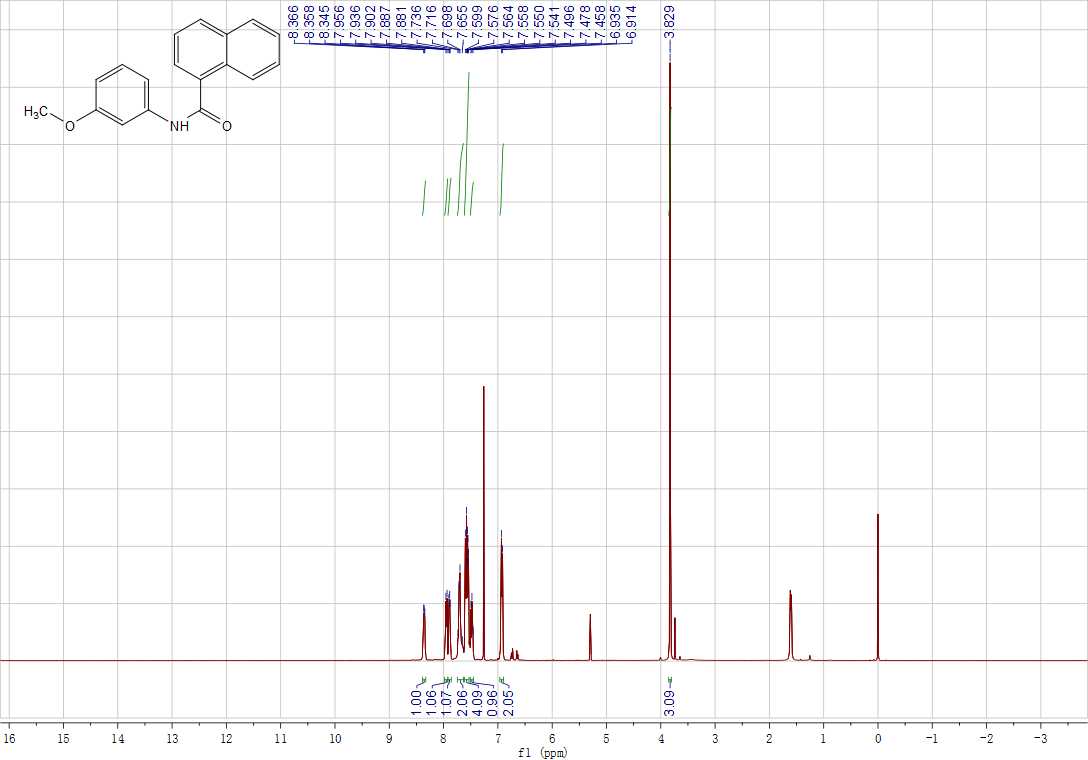


^1^H NMR spectra of compound **2q**


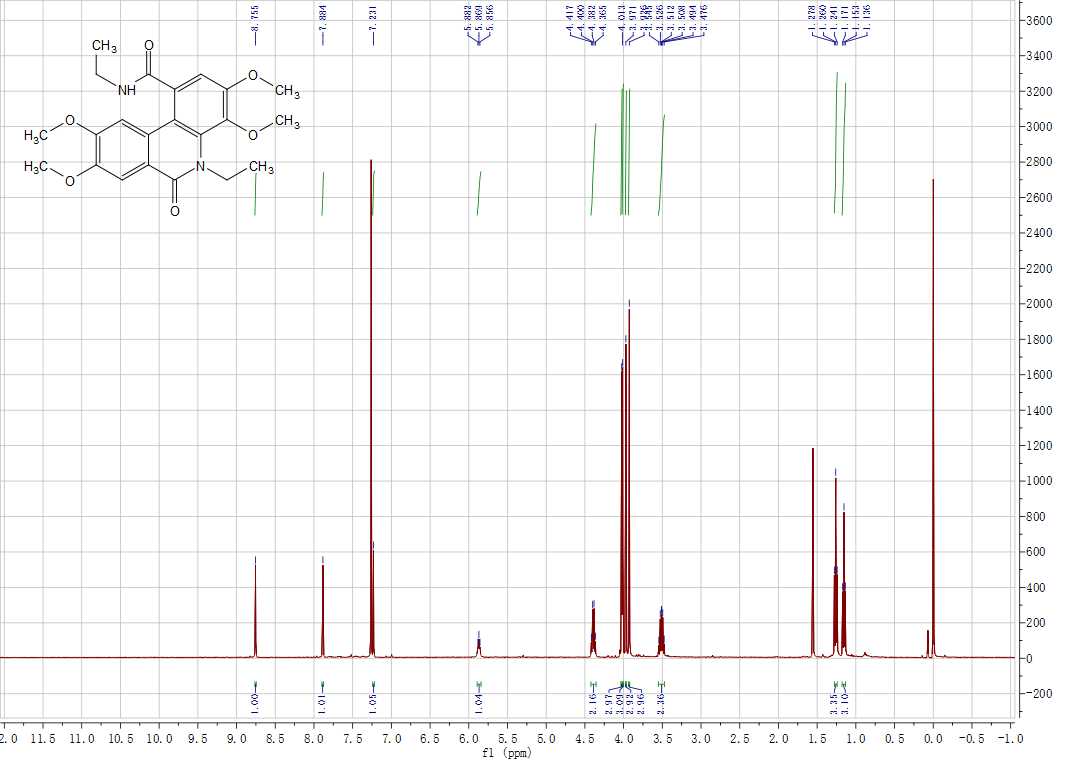

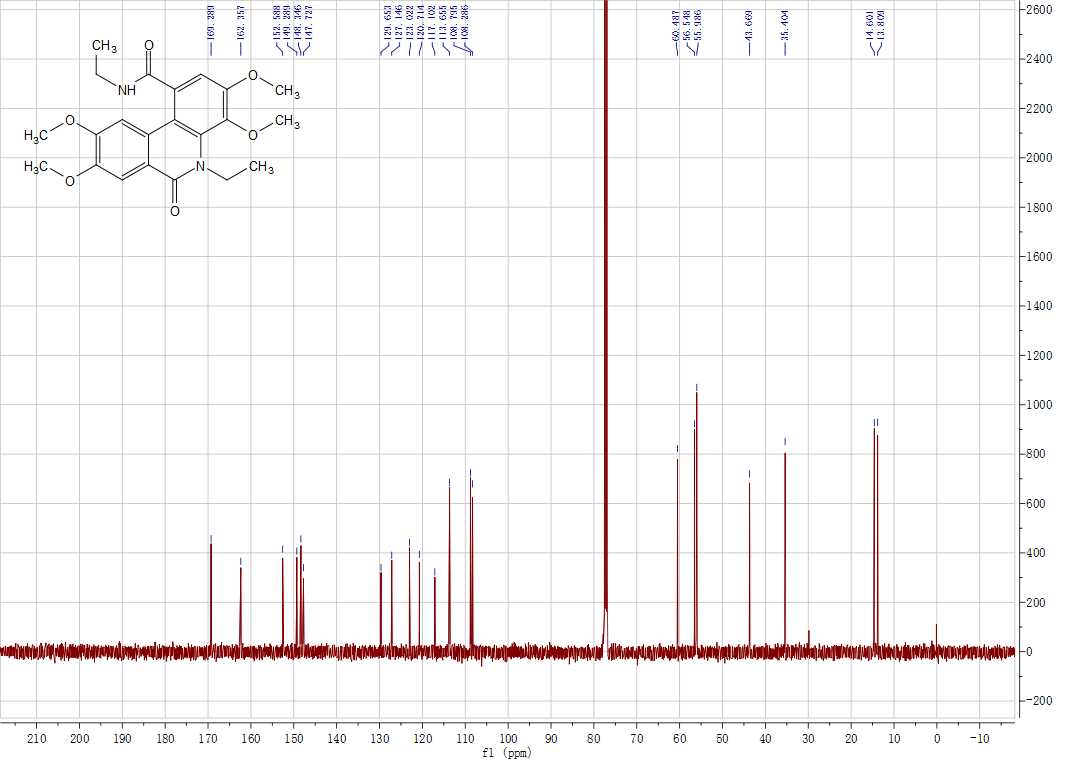


^1^H NMR and ^13^C NMR spectra of **3a**


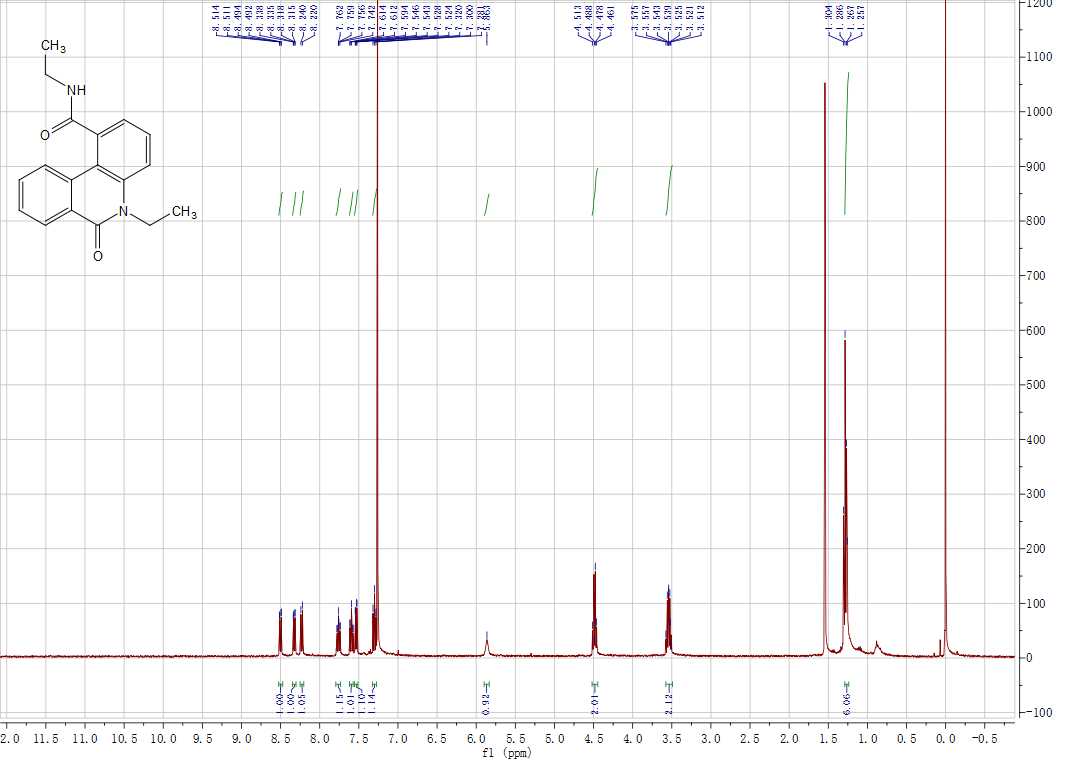

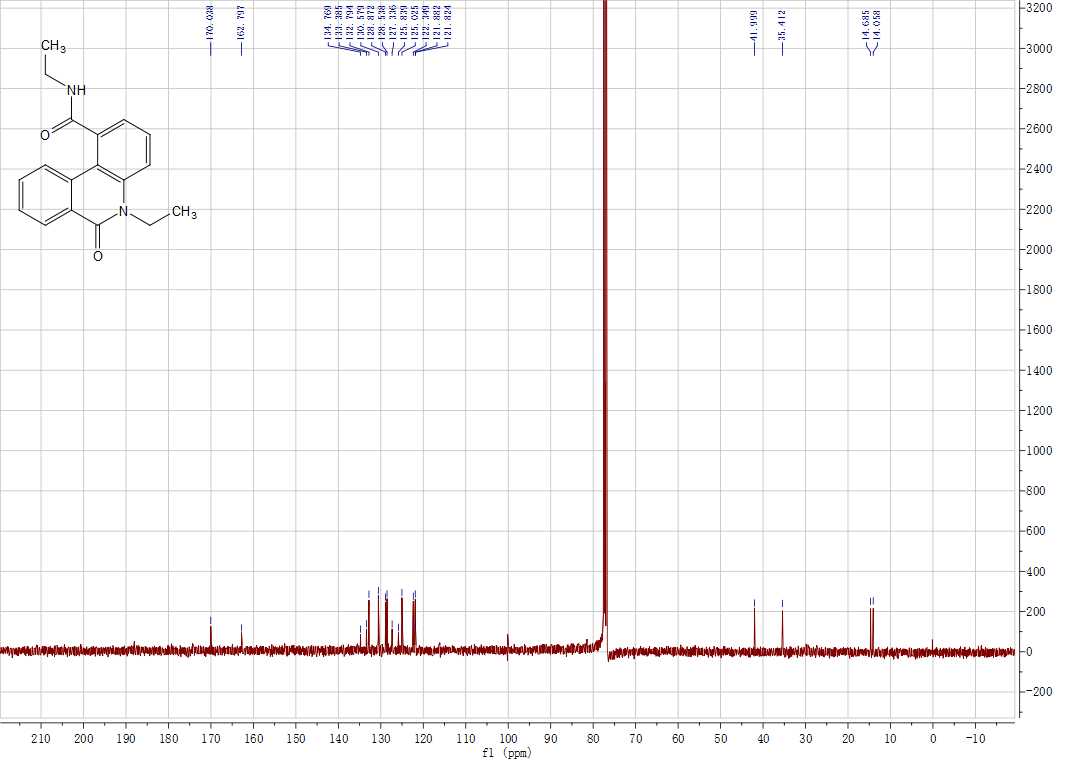


^1^H NMR and ^13^C NMR spectra of **3b**


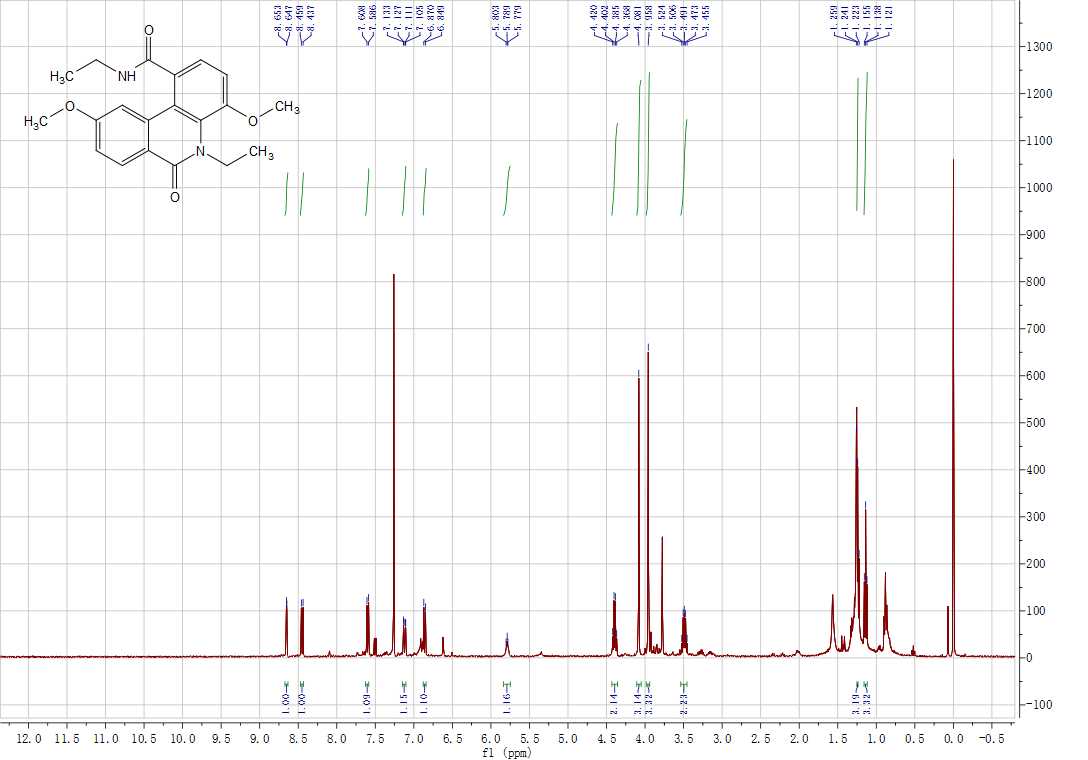

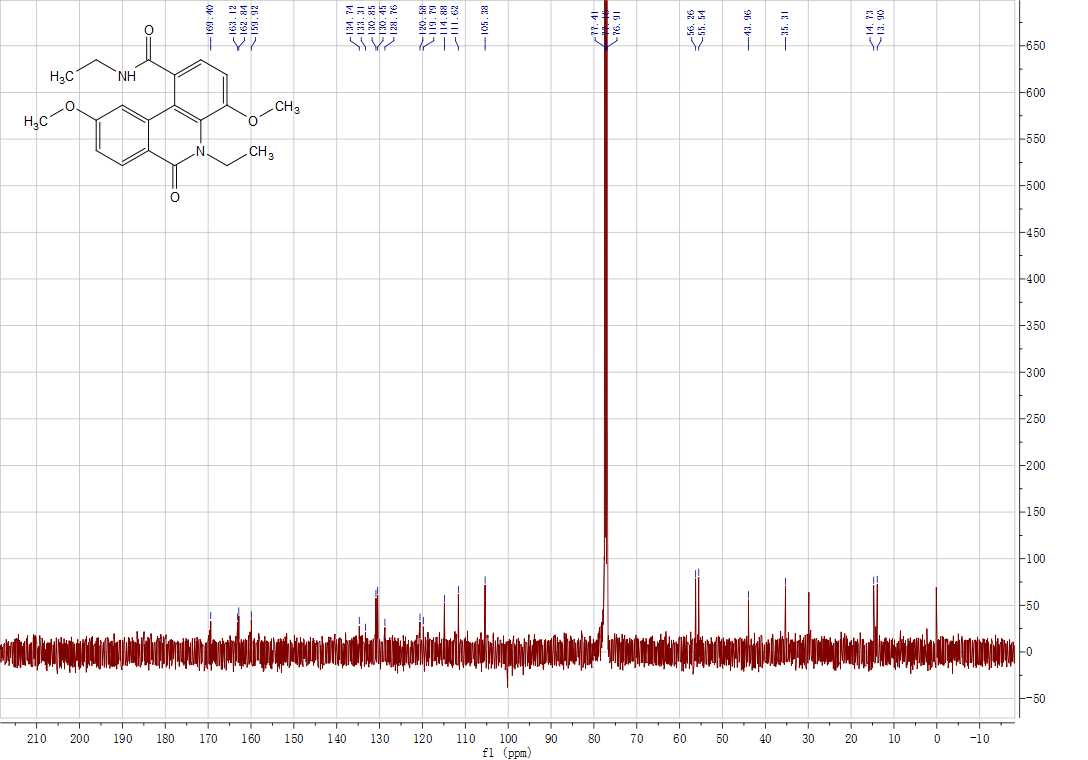


^1^H NMR and ^13^C NMR spectra of **3c**


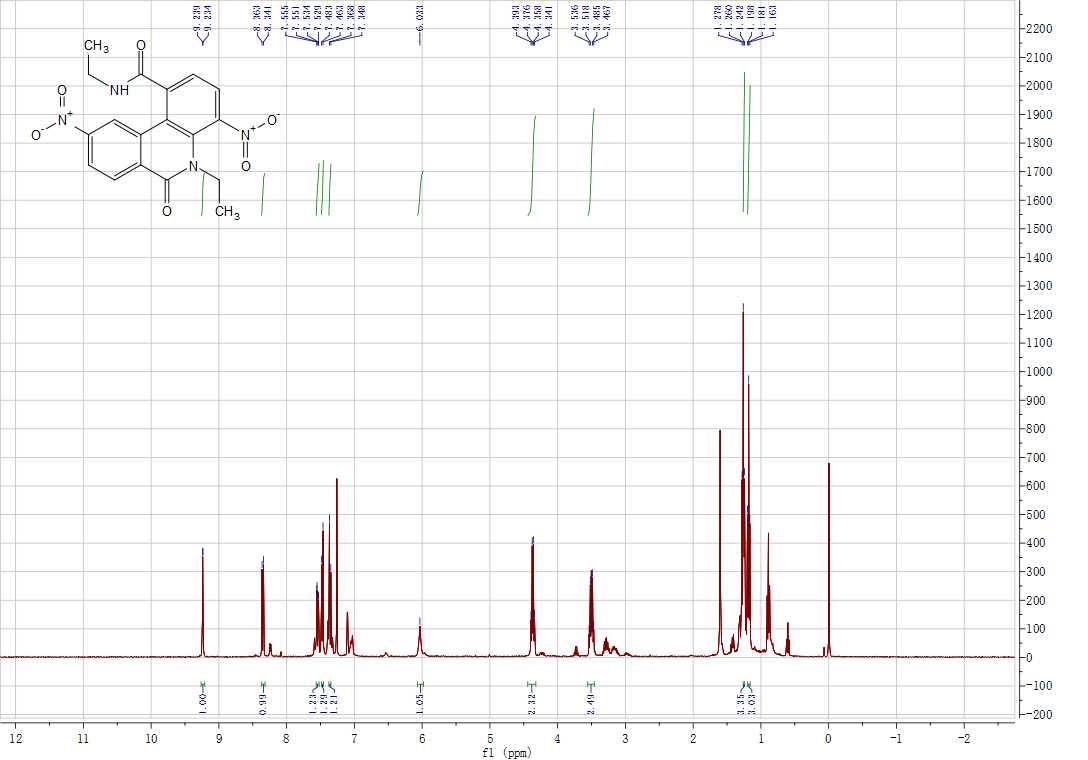


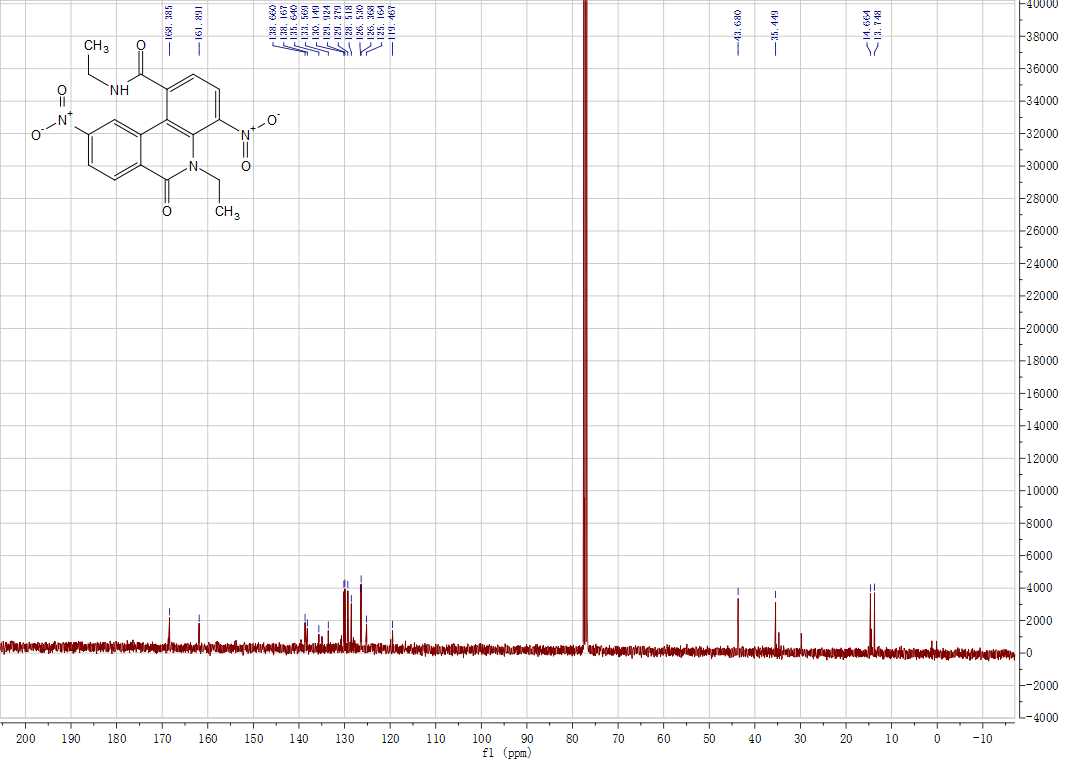


^1^H NMR and ^13^C NMR spectra of **3d**


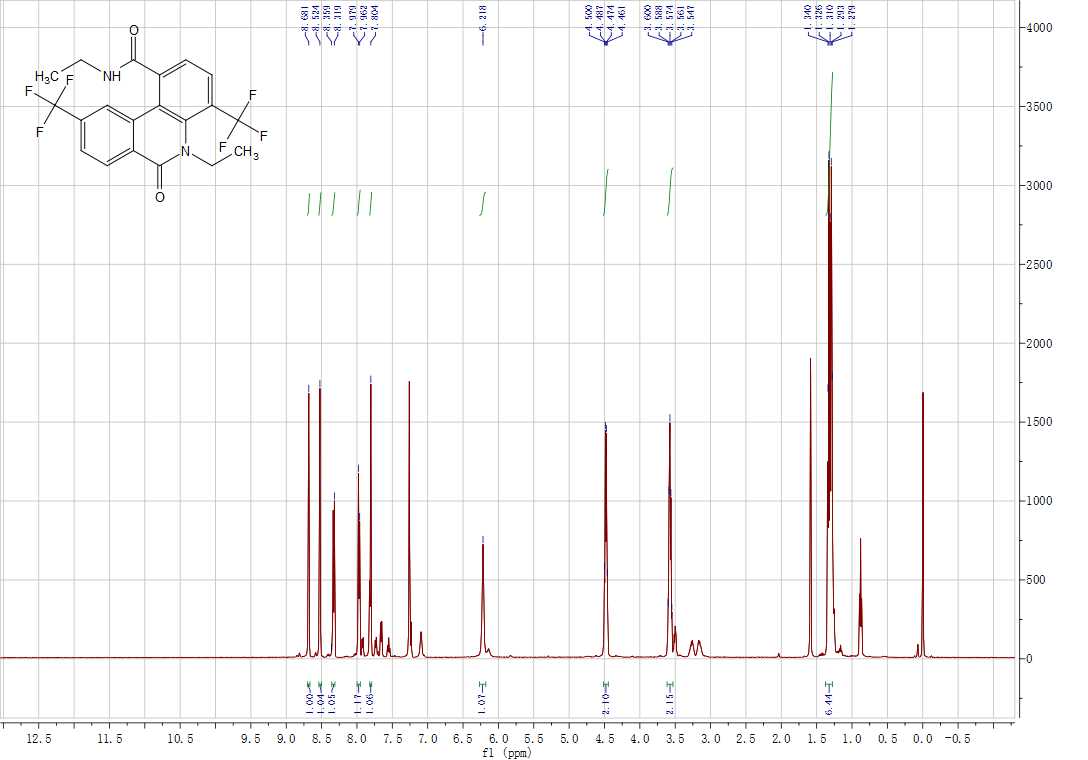

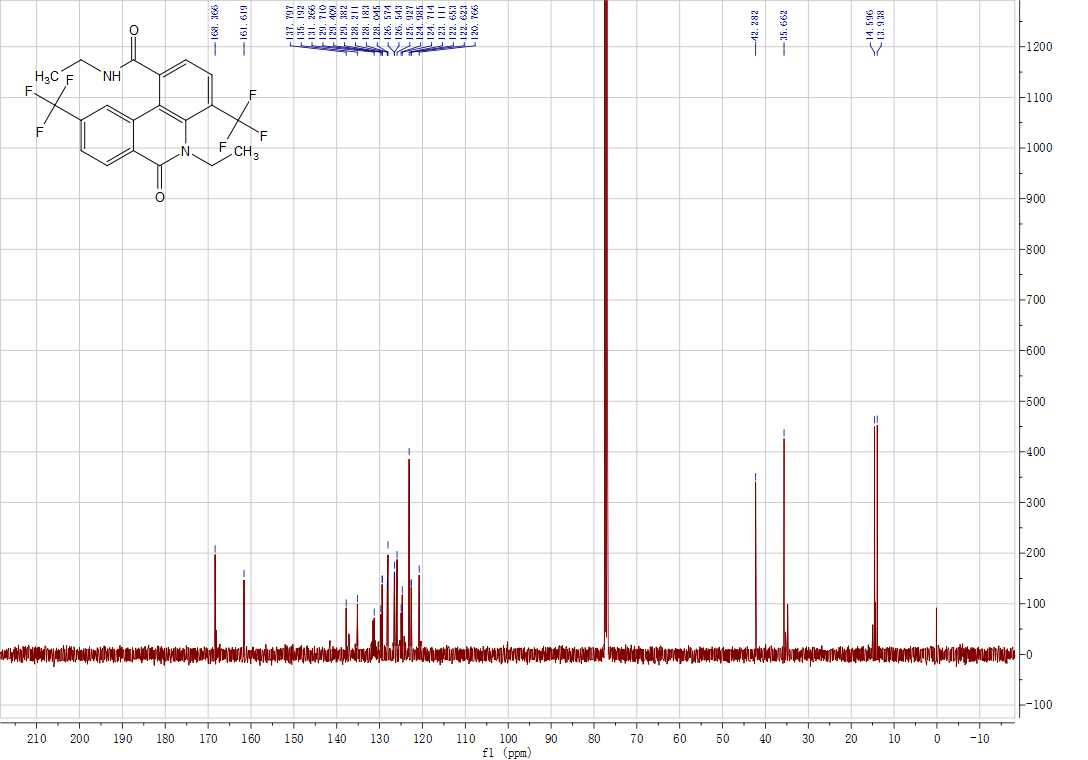


^1^H NMR and ^13^C NMR spectra of **3e**


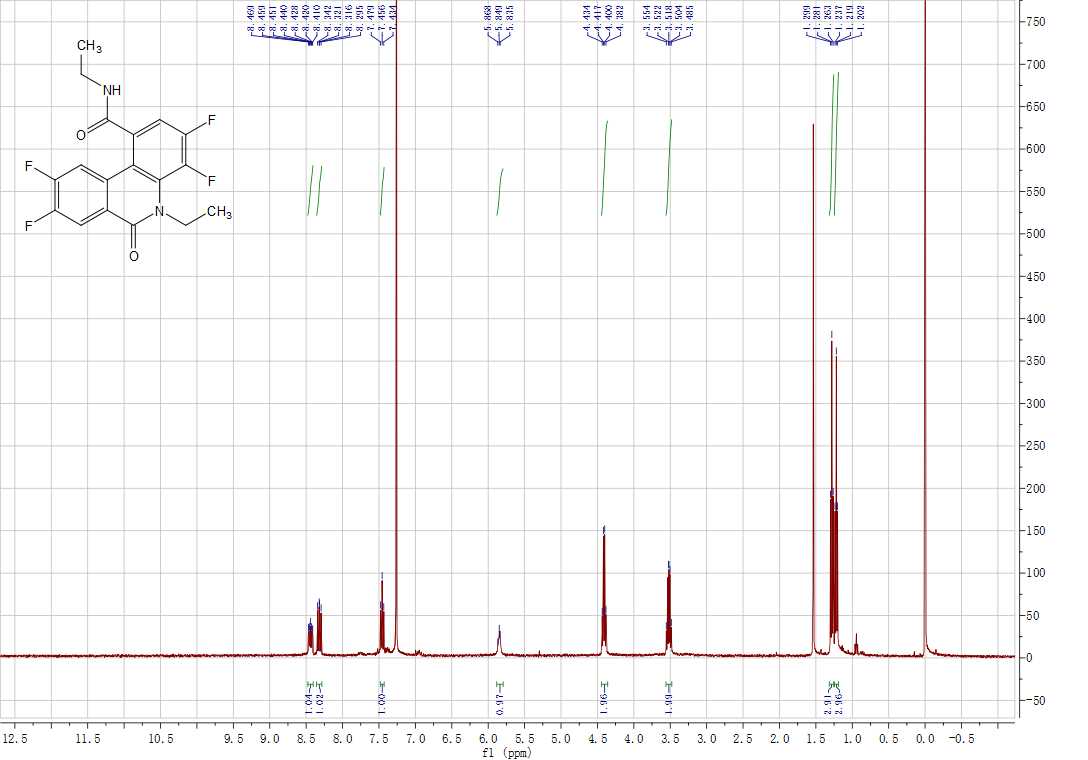

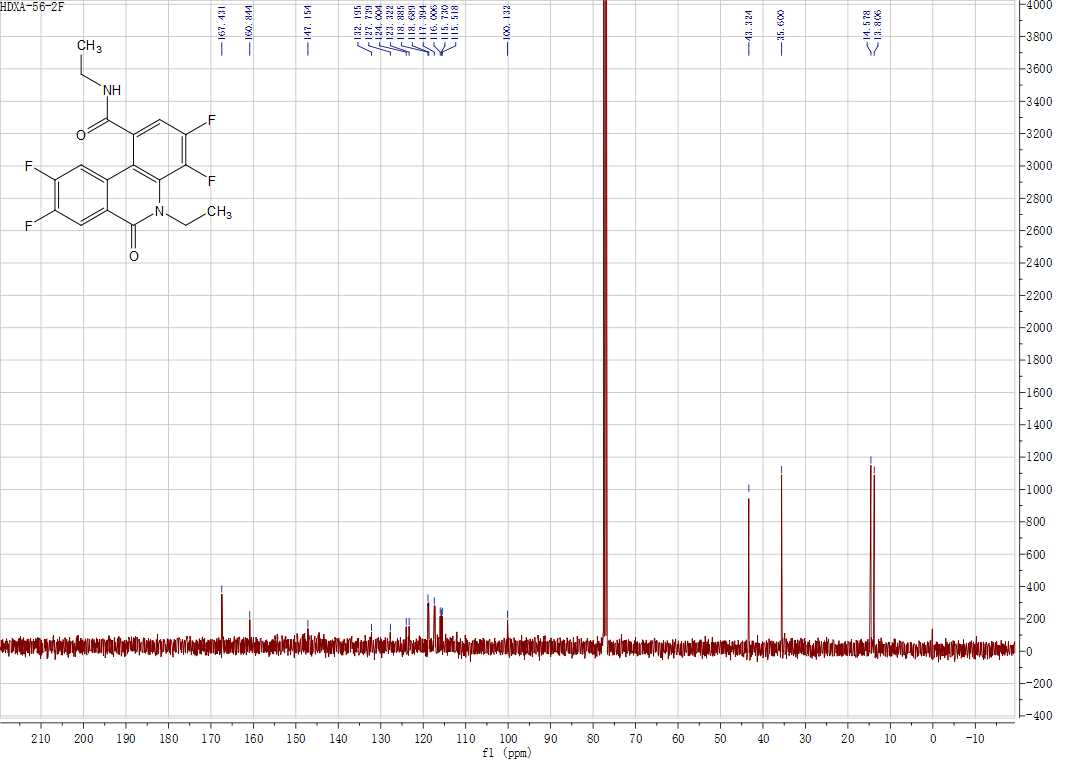


^1^H NMR and ^13^C NMR spectra of **3f**


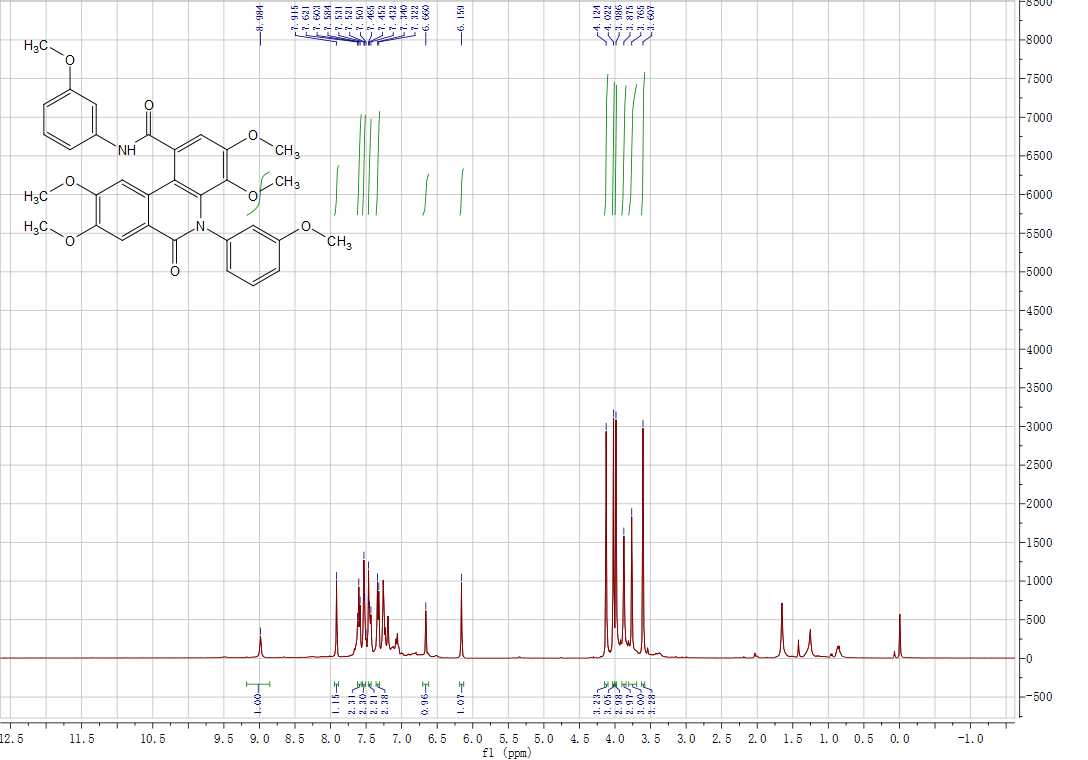

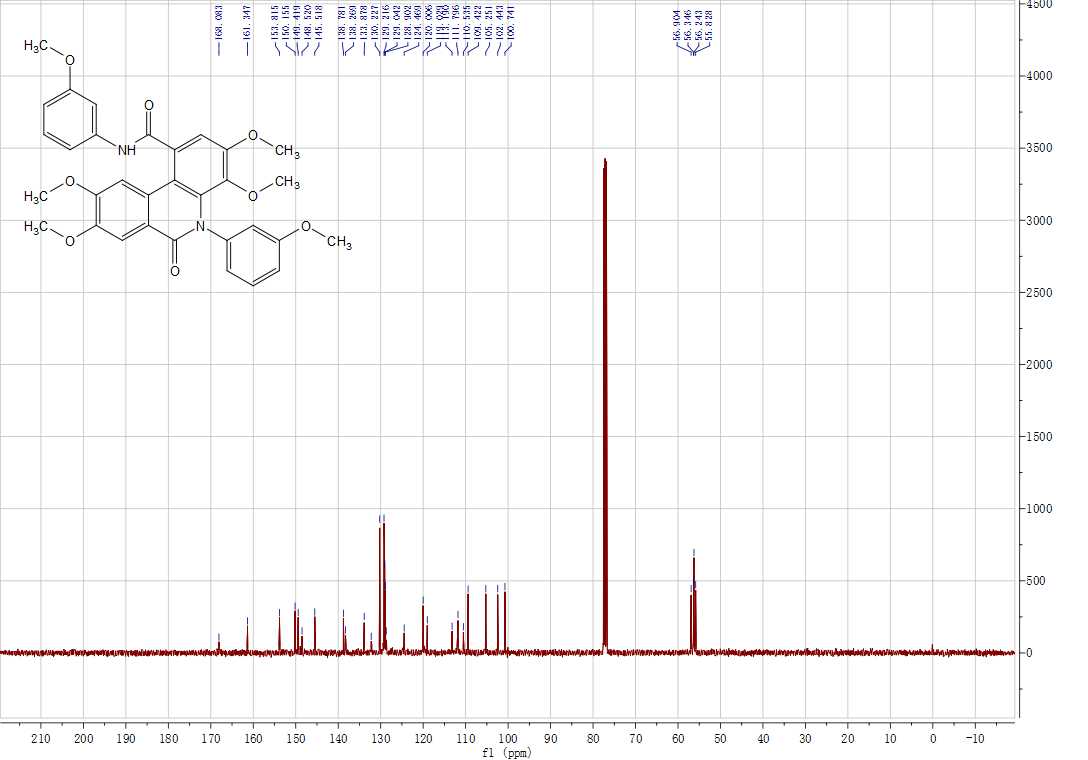


^1^H NMR and ^13^C NMR spectra of **3g**


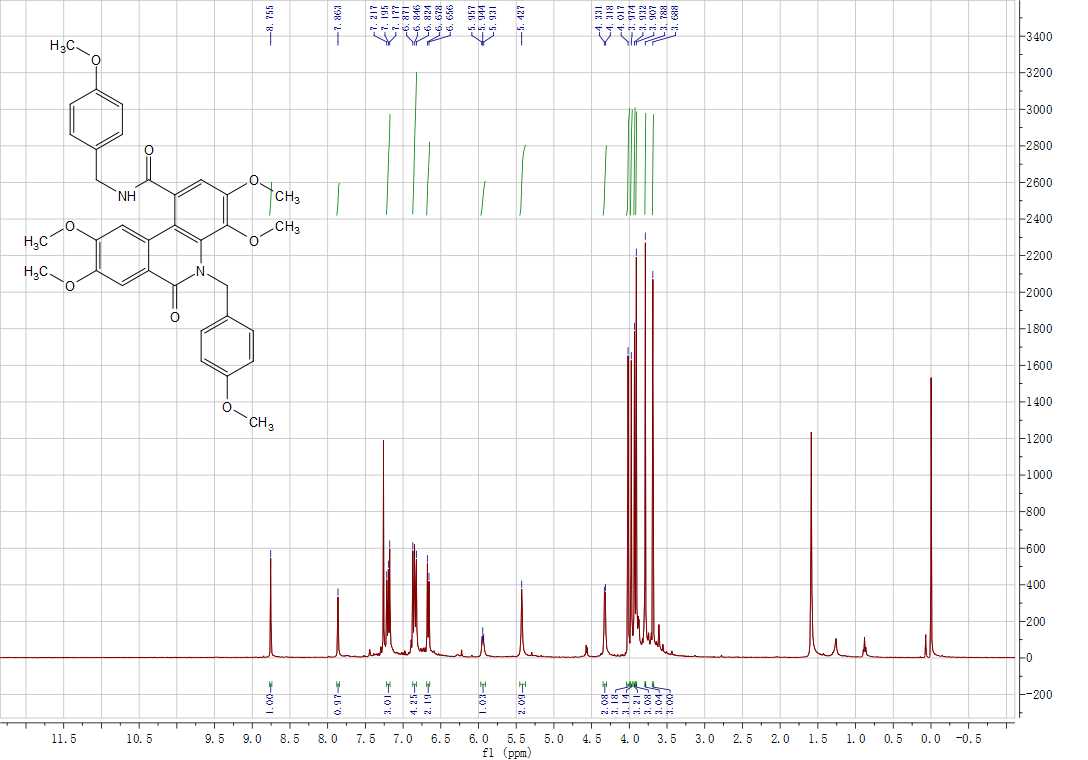

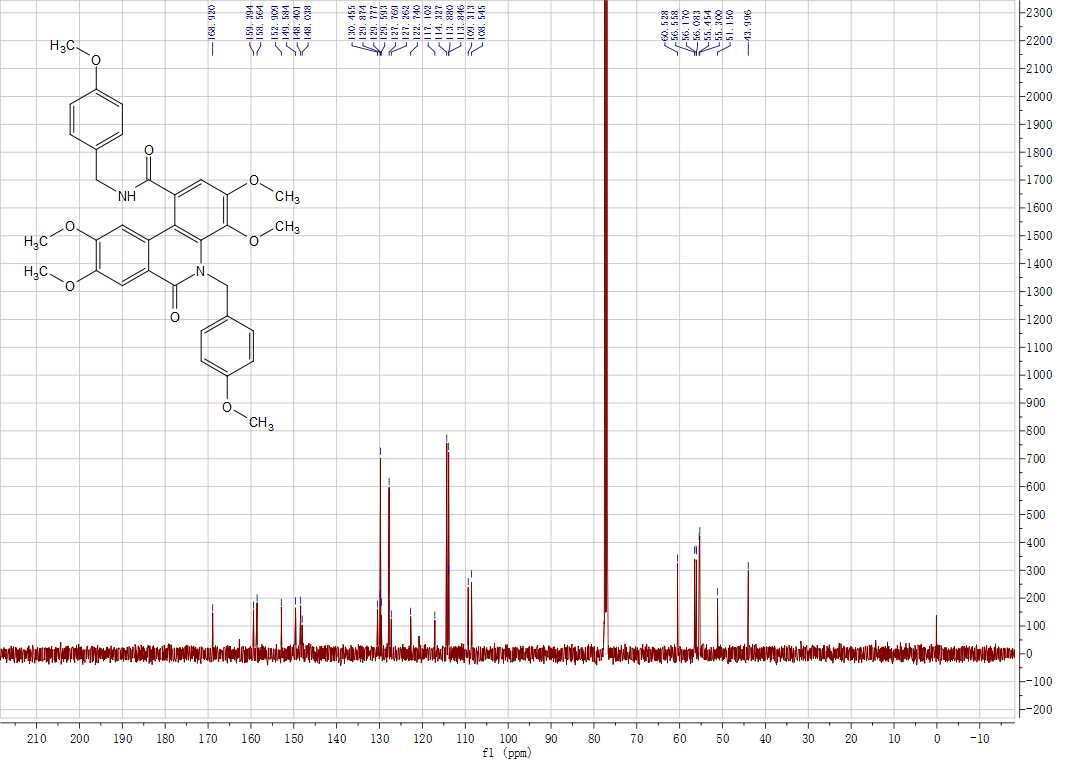


^1^H NMR and ^13^C NMR spectra of **3h**


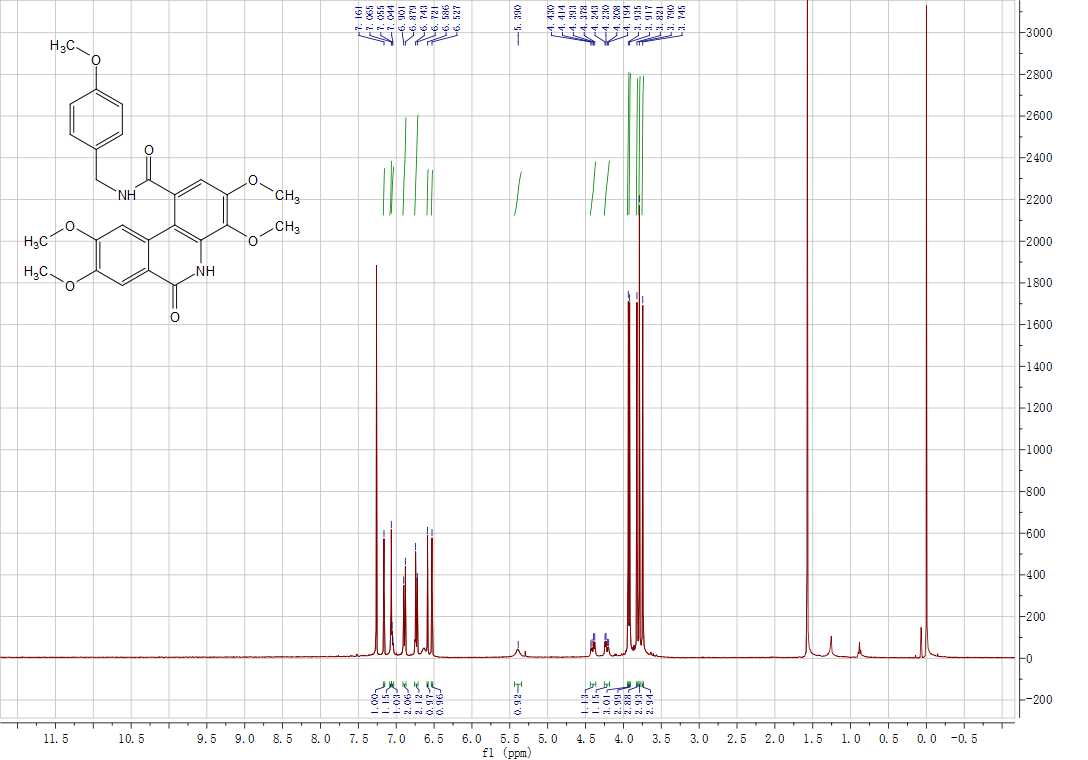

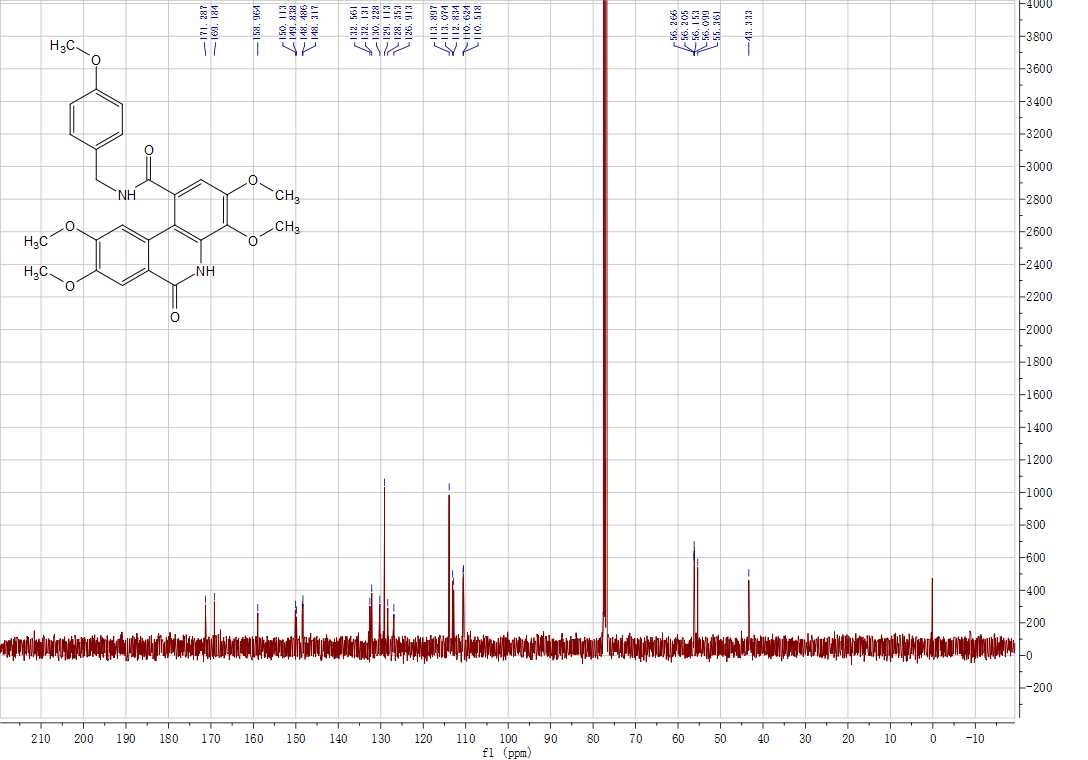


^1^H NMR and ^13^C NMR spectra of **3i**


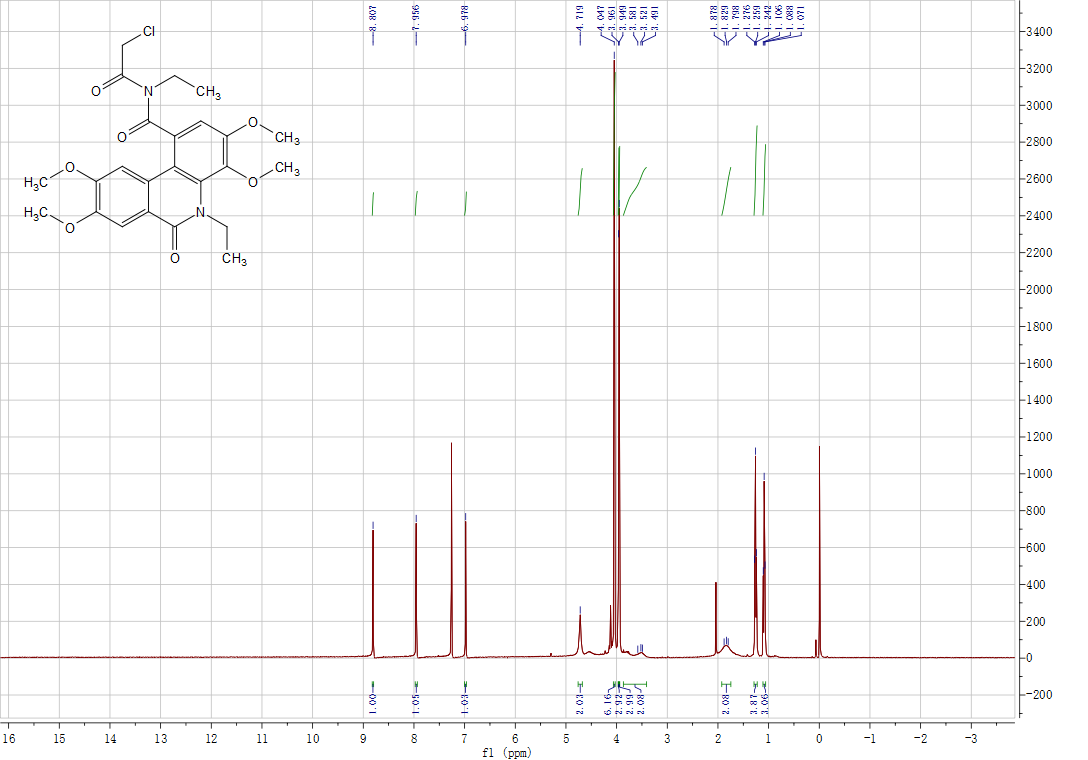

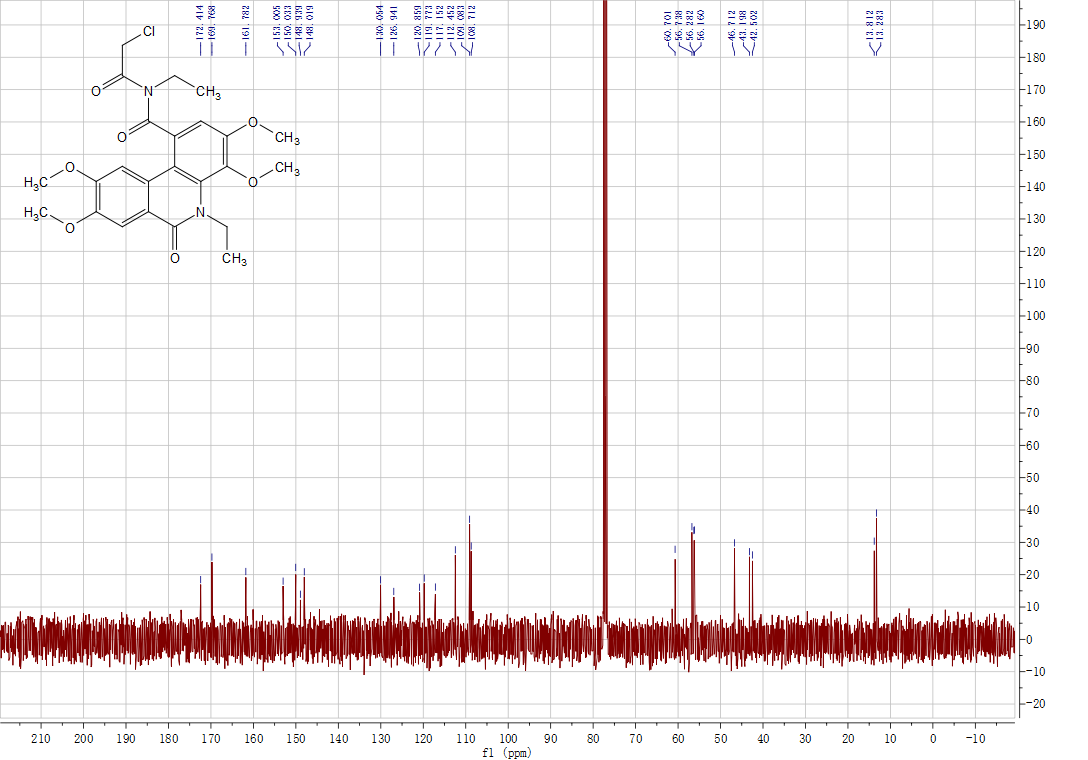


^1^H NMR and ^13^C NMR spectra of **4a**


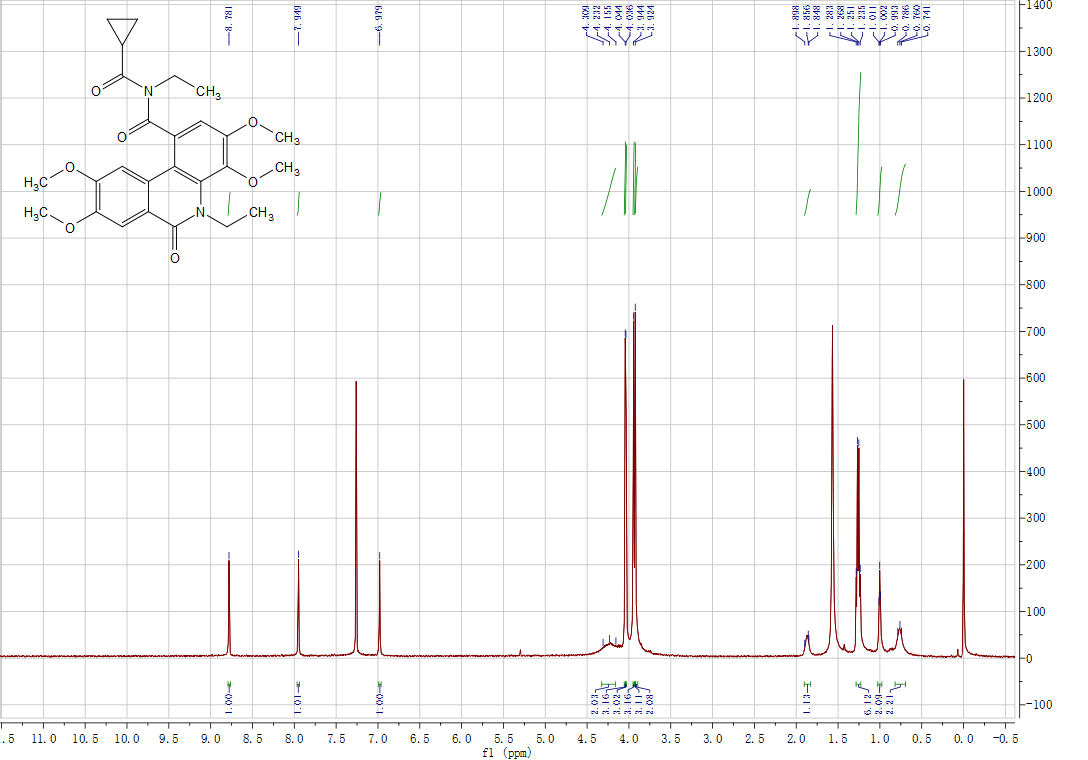


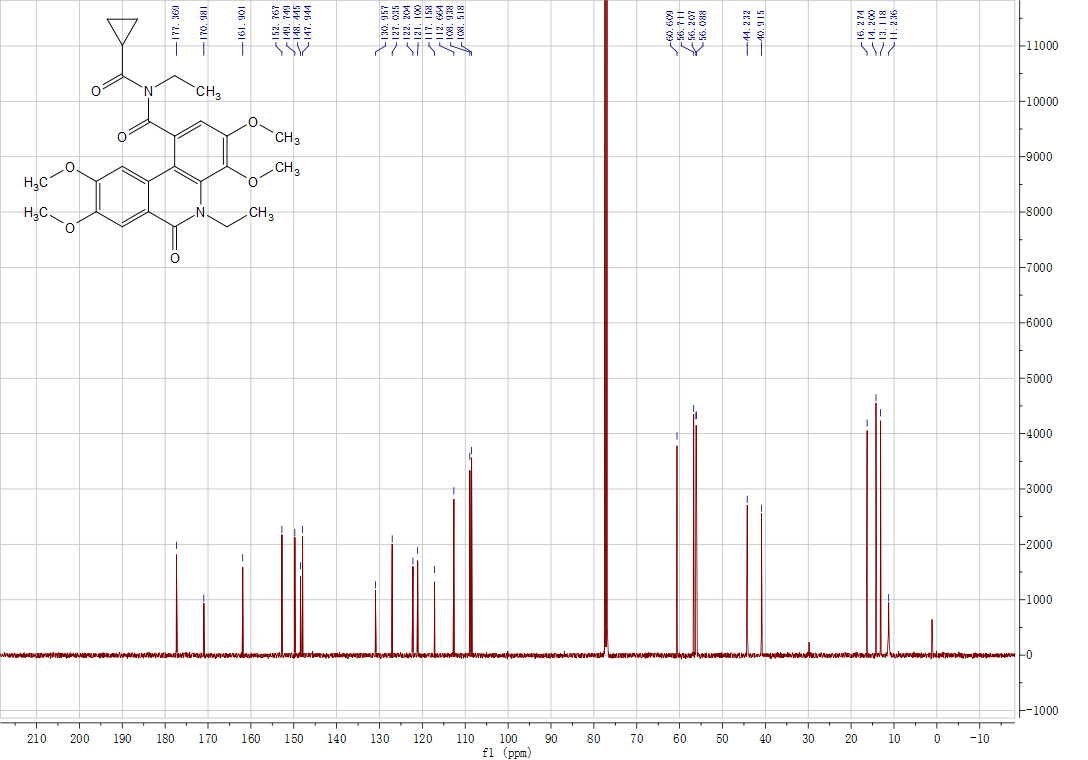


^1^H NMR and ^13^C NMR spectra of **4b**


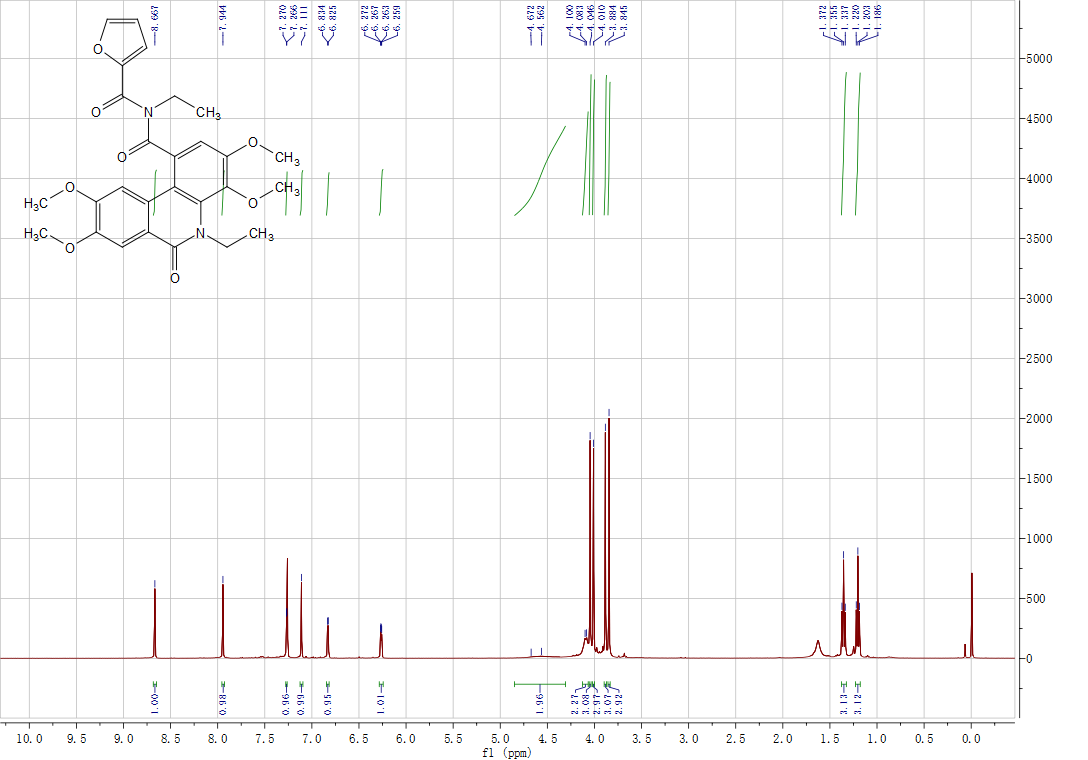


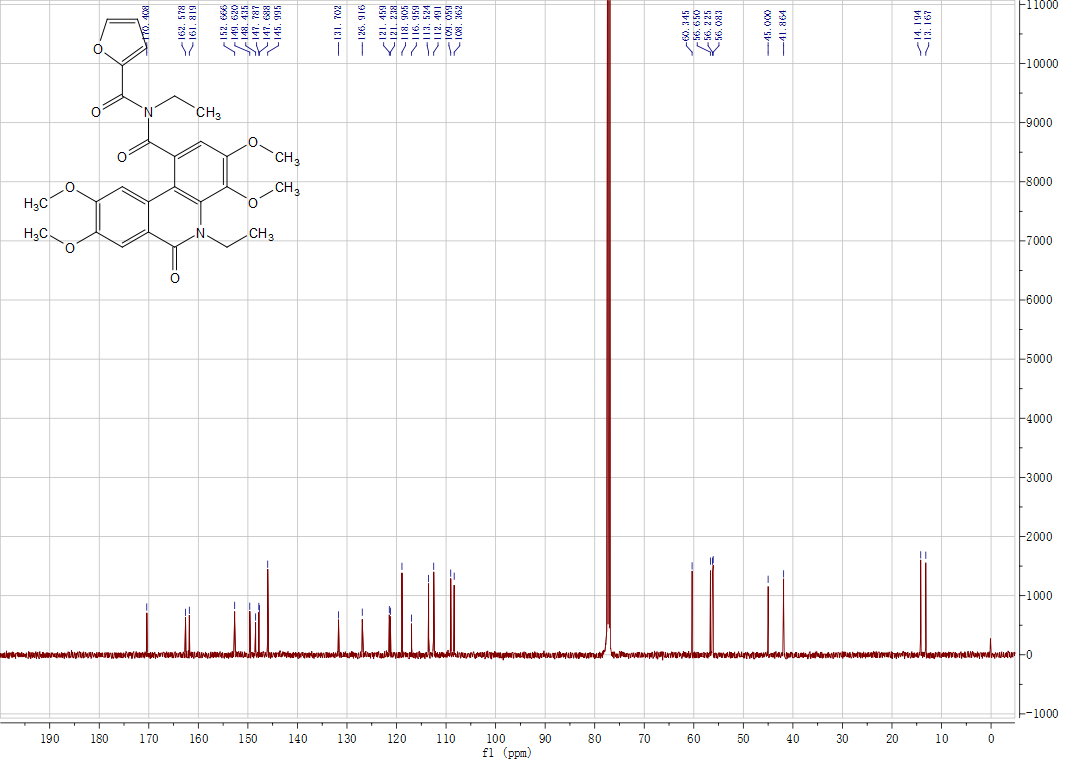


^1^H NMR and ^13^C NMR spectra of **4c**


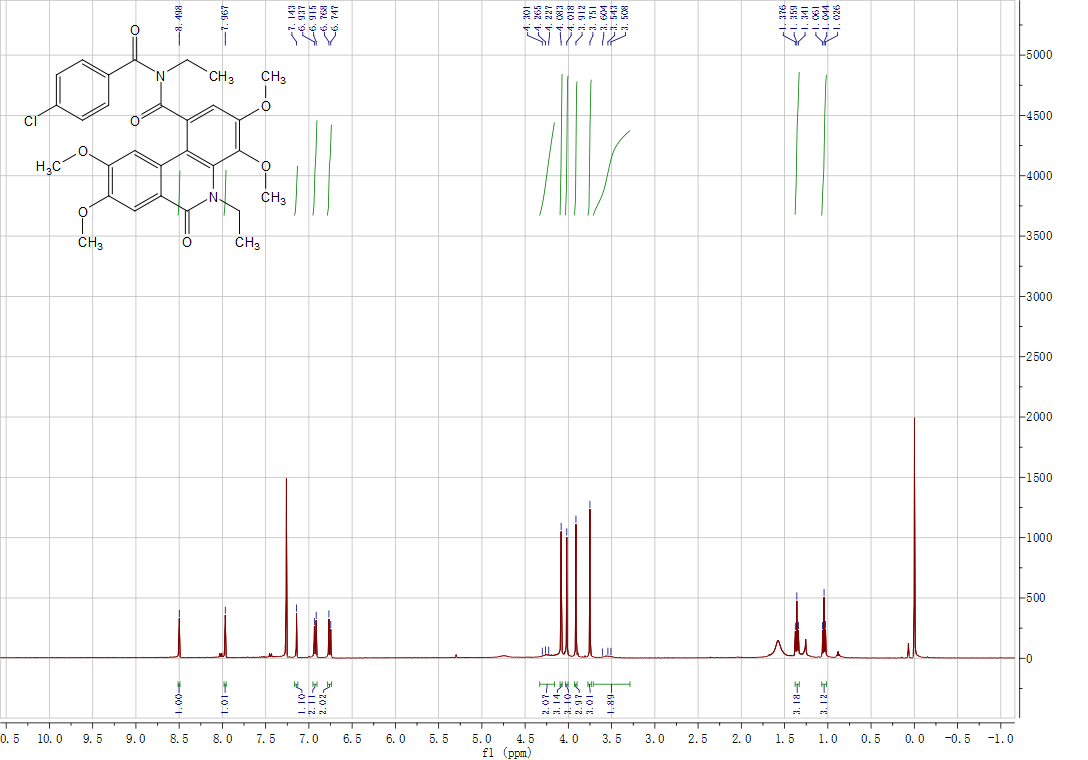


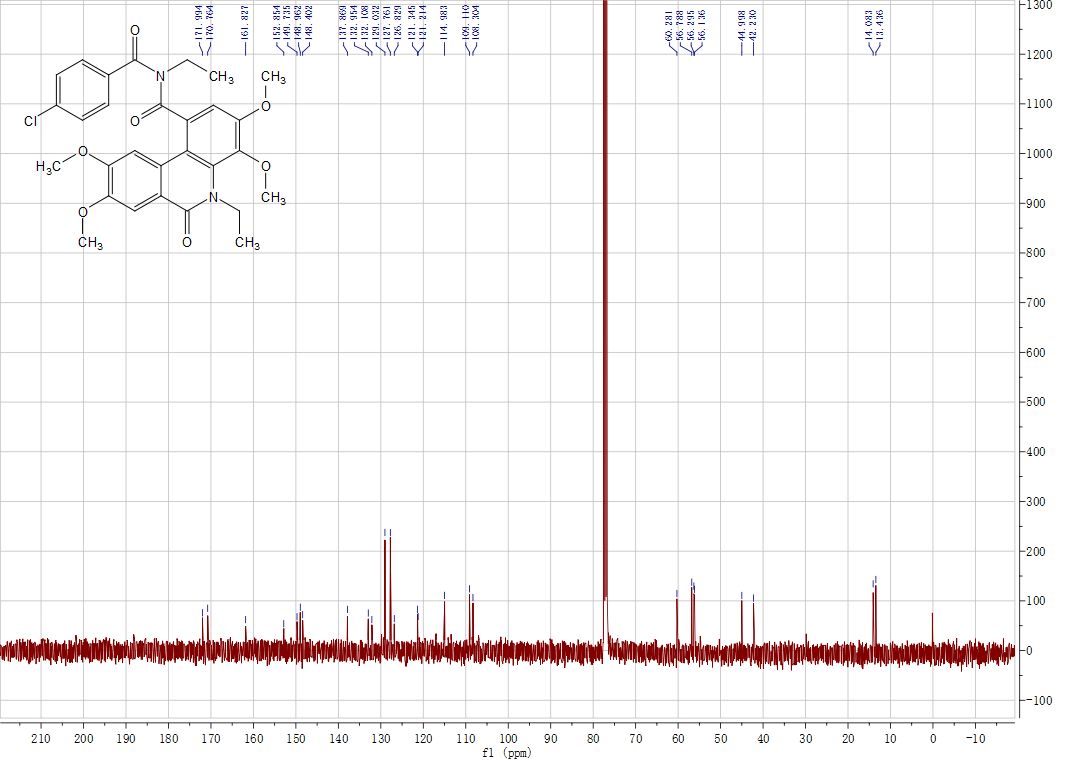


^1^H NMR and ^13^C NMR spectra of **4d**


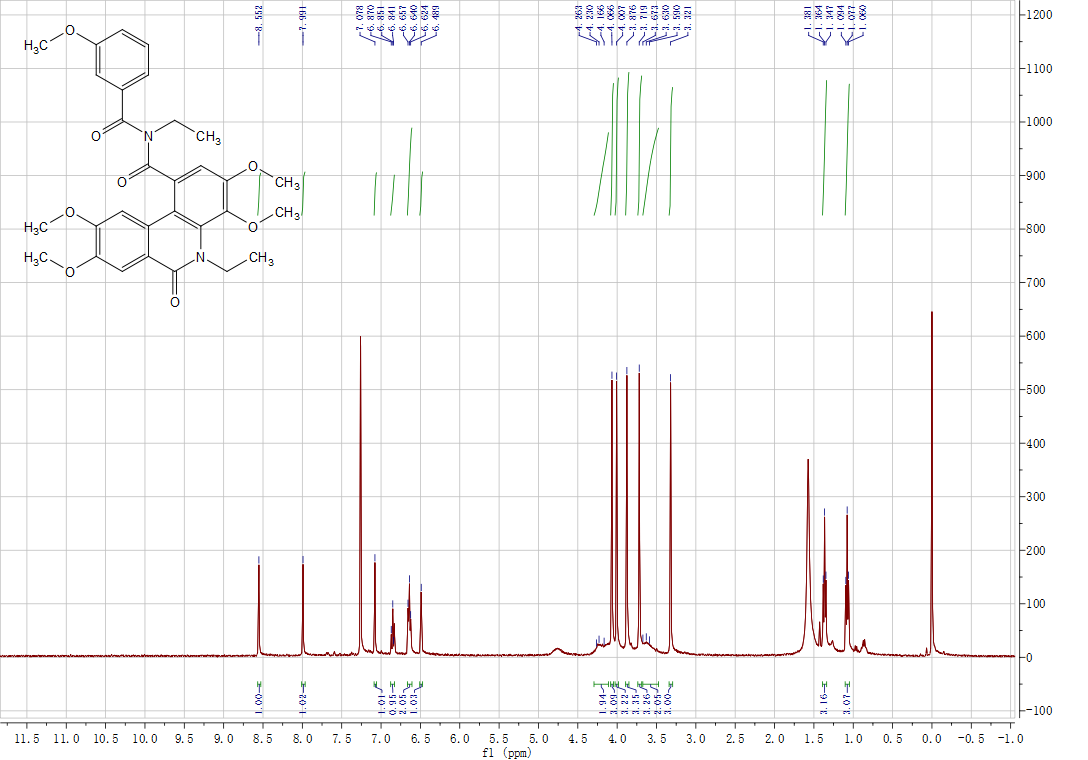


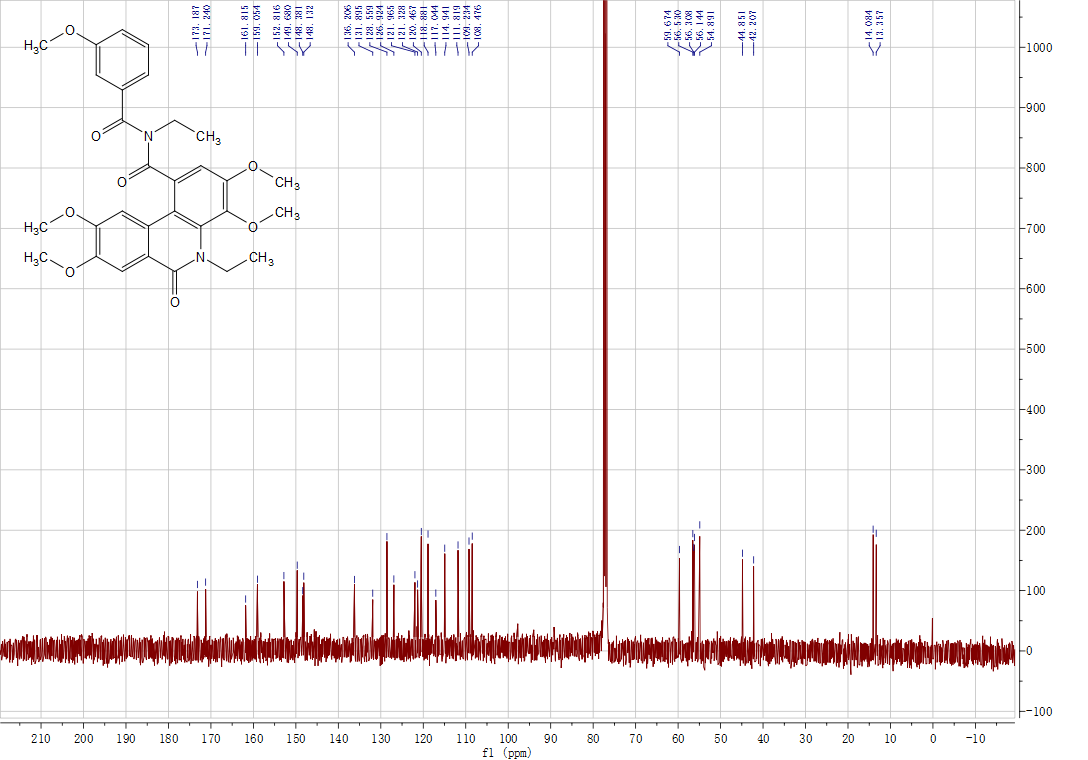


^1^H NMR and ^13^C NMR spectra of **4e**
